# Leptospiral Leucine-Rich Repeat Protein-Based Lateral Flow for Assessment of Canine Leptospiral Immunoglobulin G

**DOI:** 10.3390/tropicalmed7120427

**Published:** 2022-12-10

**Authors:** Sineenat Sripattanakul, Kanpapat Boonchuay, Teerasak Prapong, Worawidh Wajjwalku, Gerd Katzenmeier, Dietmar Haltrich, Ratchanee Hongprayoon, Siriwan Prapong

**Affiliations:** 1 The Interdisciplinary Graduate Program in Genetic Engineering, The Graduate School, Kasetsart University, Bangkok 10900, Thailand; 2 Faculty of Veterinary Medicine, Kasetsart University, Bangkok 10900, Thailand; 3 Akkhraratchakumari Veterinary College, Walailak University, Nakhon Si Thammarat 80160, Thailand; 4 One-Health Research Center, Walailak University, Nakhon Si Thammarat 80160, Thailand; 5 Department of Food Sciences and Technology, University of Natural Resources and Life Sciences, 1180 Vienna, Austria

**Keywords:** leucine-rich repeat (LRR), lateral flow strip test, canine, leptospirosis, tropical infectious diseases, emerging and re-emerging diseases, diagnosis

## Abstract

The recombinant, modified leucine-rich repeat protein rhKU_Sej_LRR_2271 has been suggested as a candidate for leptospiral vaccine development since it was predicted to be a transmembrane protein containing leucine-rich repeat motifs and immunogenic epitopes. The immunogenic epitopes showed binding affinities with lower IC_50_ values than peptides of known antigenic proteins, e.g., LipL32. Moreover, this protein was immunoreactive with hyperimmune sera against several serovars. In this study, we aimed to develop a lateral flow strip test using the rhKU_Sej_LRR_2271 protein for the detection of anti-leptospiral IgG in dogs. The lateral flow assay was performed with 184 dog plasma samples and evaluated with a culture method, 16S ribosomal RNA gene (*rss*) analysis real-time PCR, and LipL32 ELISA. The culture method failed to detect leptospires in the dog blood samples. Six of nine symptomatic dogs gave positive results with the real-time PCR assay. The lateral flow assay and LipL32 ELISA gave positive results with 59 and 50 dogs, respectively. The sensitivity, specificity, and accuracy of the rhKU_Sej_LRR_2271 lateral flow strip test were 70.00, 82.09, and 78.80%, respectively, when compared with LipL32 ELISA. There was a significant association between the LipL32 ELISA and the rhKU_Sej_LRR_2271 lateral flow assay. The rhKU_Sej_LRR_2271 lateral flow strip test has therefore demonstrated a good potential to detect anti-leptospiral IgG in dogs.

## 1. Introduction

Leptospirosis is a re-emerging zoonotic disease caused by the pathogenic spirochete of the genus *Leptospira*. Currently, 41 pathogenic species and 28 saprophytic species with more than 300 serovars have been described [1]. In general, the disease severity can range from asymptomatic to lethal, depending on the serotypes of *Leptospira* and the host [2]. Leptospirosis can affect both humans and animals. Dogs are a companion animal that are close to humans and often considered to be family members. The estimated dog population in Thailand in 2016 was around 7 million dogs, and around 11% were stray dogs [3]. The stray dog population increases annually and has become a public health concern in Thailand [4]. Moreover, stray dogs cause many problems in urban and rural communities, e.g., road accidents, dog attacks, and disease transmissions (parasites, rabies, and leptospirosis). The prevalence of leptospirosis in stray dogs in Bangkok province, Thailand was reported to be as high as 89.1% using the microscopic agglutination test (MAT) [4]. Even though there is little evidence that dogs directly transfer leptospirosis to humans. The serotypes of *Leptospira* found in dogs [4,5,6,7,8,9,10,11,12,13] are identical with the serotypes found in rodents [12,14,15], domestic animals [7,16], and humans [12,15,17]. Accordingly, dogs are considered an important carrier or reservoir of leptospirosis [13,18,19,20,21,22].

Currently, a number of methods for the diagnosis of leptospirosis are known. Polymerase chain reaction (PCR) and real-time PCR are highly sensitive molecular methods that detect and amplify specific genes of *Leptospira*. These are suitable techniques for early detection during a leptospiremia phase. Moreover, they are also beneficial for studying contaminated environments and the shedding of animals during the leptospiruria phase. The MAT is the gold standard test that identifies leptospiral serogroups and is useful for epidemiology. However, MAT sensitivity is low in the initial phase of infection. Moreover, the MAT is time-consuming and requires an expert to perform and maintain *Leptospira* live reference strains [23]. Bacterial isolation and culturing are standard techniques for demonstrating the presence of live *Leptospira* in biological samples, useful for both epidemiology and outbreaks. However, *Leptospira* culturing is not recommended for clinical tests due to the 6-8 weeks required for incubation and its low sensitivity for diagnosis [23]. An enzyme-linked immunosorbent assay (ELISA) is one of the serological tests presenting high sensitivity and specificity during the immune phase [23]. Rapid diagnostic tests, e.g., a lateral flow assay (LFA) or a immunochromatographic assay, are widespread for screening tests because they are user-friendly, low-cost, portable, and do not require special equipment [23]. Many antigens and genes of *Leptospira* were studied and used for developing diagnostic tests, e.g., LipL21, LigA, Loa22, and LipL32 [24,25,26,27,28,29]. In addition, the proper antigens for developing test kits should allow for differentiation between natural infection and vaccinal immunity.

Leucine-rich repeat (LRR) proteins can be found in every kingdom of living organisms. The functions of these LRR motifs have been predicted to occur as protein-protein or protein-ligand interactions. Interestingly, several LRR proteins of pathogens are involved in the host-cell adhesion, invasion, and stimulation of host immune responses, e.g., YopM in *Yersinia pestis*, IpaH in *Shigella flexneri*, SspH and SlrP in *Salmonella* spp., Slr in *Streptococcus* sp., LrrA in *Treponema denticola*, and LRR20 in *L. santarosai* [30,31,32]. Moreover, pathogenic strains of *Leptospira* encode higher numbers of LRR proteins than saprophytic strains [33]. In addition, several LRR proteins, e.g., LBJ_2012 and LBJ_2271 in *L. borgpetersenii* serovar hardjo-bovis strain JB197, were predicted to contain epitopes with binding affinities equal to or lower than other leptospiral antigenic proteins such as LipL32, LigA, LipL36, LipL41, and OMPL1 as judged by the IC_50_ values [34,35]. Accordingly, the *Leptospira* LRR proteins may be of interest as alternative candidates for the development of diagnostic tools for leptospirosis.

Recently, Nitipan identified and cloned orthologous LRR genes of LBJ_2271 from *L. borgpetersenii* serovar Sejroe strain M84 [34]. The recombinant plasmid encoded the protein KU_Sej_LRR_2271 which had 98.5% identity of the protein sequence of LBJ_2271. The KU_Sej_LRR_2271 was characterized using bioinformatic tools as a transmembrane protein consisting of LRR domains and a N-glycosylation site [34,35]. Sritrakul et al. cloned and expressed the recombinant hybrid (rh) KU_Sej_LRR_2271 protein, which was fused to the signal sequence of LBJ_2271 [36]. The protein rhKU_Sej_LRR_2271 had IC_50_ values which were equal to or lower than those of LigA, LipL32, OMP1, and LipL36. Moreover, the protein rhKU_Sej_LRR_2271 showed immunoreactivity to hyperimmune sera against the serovars Sejroe, Bullum, Bratislava, and Icterohaemorrhagiae and it was proved to have the ability to induce both humoral and cell-mediated immune responses in a rabbit model [36,37].

The aim of this study was to use the protein rhKU_Sej_LRR_2271 for the development of a lateral flow test kit to analyze canine antisera against *Leptospira* and compare it with other techniques such as culture, real-time PCR, and ELISA. Rabbit hyperimmune sera against nine serovars of *Leptospira* spp. were used to analyze the antigenic specificity of our recombinant protein.

## 2. Materials and Methods

### 2.1. Biological Samples

Whole blood samples of dogs (*Canis lupus familiaris*) were collected from 91 stray dogs and 93 family dogs from the areas of Bangkok (BKK) and Ubon Ratchathani (UBR) province in Thailand during October to December 2019. The dog blood samples were collected during sterilization and the rabies vaccination program was organized by the Soi Dog Foundation (Bangkok) using EDTA tubes (BD Vacutainer K2EDTA; Becton, Dickinson and Company). Furthermore, 20 dog sera were received from the sera bank of the Veterinary Teaching Animal Hospital of Walailak University. These 20 dogs were all vaccinated twice by commercial vaccines against 4 serovars of *Leptospira*, and had no history of leptospirosis infection. These 20 dog sera were assigned as sera of “Vaccinated-dogs”. After sterilization and/or rabies vaccination, the family dogs were returned to their owner and the stray dogs were released to their places. Rabbit hyperimmune sera against *Leptospira* spp. were purchased from the OIE Reference Laboratory for Leptospirosis, Academic Medical Center, Department of Medical Microbiology, University of Amsterdam. The non-immunized rabbit serum was obtained from the Center for Agricultural Biotechnology with the standard immunization protocol. All protocols for both dog and rabbit sample collections were approved by the Kasetsart University Institutional Animal Care and Use Committee (Kasetsart University-IACUC).

### 2.2. Bacteria Used in This Study

In total, 24 serovars of *Leptospira* spp. were obtained from the National Institute of Animal Health, Department of Livestock Development, Ministry of Agriculture and Cooperatives, Thailand. The data are shown in Supplementary Table S1. *Aerococcus viridans*, *Escherichia coli* strain DH5α, *Salmonella* sp., and *Streptococcus aureus* were obtained from the Department of Microbiology and Immunology, Faculty of Veterinary Medicine, Kasetsart University, Thailand.

### 2.3. Recombinant Proteins

The protein rhKU_Sej_LRR_2271 was produced by *E. coli* BL21 StarTM (DE3), which contained pET160_hKU_R21_2271 plasmids. Recombinant gene expression was performed as previously reported [36,37]. Briefly, gene expression was induced with 1 mM isopropyl β-D-1-thiogalactopyranoside (IPTG; Sigma–Aldrich, St. Louis, MO, USA) and cells were incubated at 37 °C overnight. Cells were harvested and resuspended in lysis-equilibration-wash buffer (LEW buffer; 50 mM NaH_2_PO_4_, 300 mM NaCl, pH 8.0) and then disrupted by a Misonix XL2020 sonicator (Woburn, MA, USA) with a processing time of 15 min (15 s cooling period for each 15 s bursts). The crude lysate was centrifuged at 12,000× *g* for 20 min at 4 °C. The pellet was washed twice with LEW buffer and resuspended in denaturing solubilization buffer (DS buffer; 50 mM NaH_2_PO_4_, 300 mM NaCl, 8 M urea, pH 8.0). The solubilized protein was purified under denaturing conditions using immobilized metal ion affinity chromatography (IMAC) with the Protino^®^ Ni-TED Resin (Macherey-Nagel, Düren, Germany). The purified protein was concentrated and desalted using Amicon^®^ Ultra-15 centrifugal filters, 30 kDa (Merck Millipore, Billerica, MA, USA). The 6x His-Lumio-TEV tag at the N-terminal of the recombinant protein was eliminated using TEV protease (NEB#P8112, NEB, Ipswich, MA, USA). The protein concentration was determined using a Nanodrop spectrophotometer (Thermo Scientific, Waltham, MA, USA) and the recombinant protein was analyzed by 12% SDS-PAGE stained with Coomassie Blue. The recombinant antigen LipL32 was purchased from Rekom Biotech (Granada, Spain).

### 2.4. Line Blot Immunodetection

The rhKU_Sej_LRR_2271 and LipL32 (50 µg/mL) were applied to nitrocellulose membranes (Bio-Rad, Hercules, CA, USA) using a paintbrush. The membranes were dried at room temperature for 30 min, blocked with 3% BSA in phosphate-buffered saline, pH 7.4 (PBS) for 30 min, and washed twice with PBS (pH 7.4) containing 0.05% Tween 20 (PBST). Then, the membranes were incubated with each rabbit hyperimmune sera against *Leptospira* spp. (Table 1) and the rabbit control serum at a dilution of 1:100 for 1.5 h and washed with PBST. The membranes were incubated with goat anti-rabbit IgG (Abcam, Cambridge, UK) conjugated with gold nanoparticles (Kestrel Bioscience, Pathumthani, TH) at a dilution of 1:20 for 30 min and washed with distilled water before observing the red lines on the membranes. The rhKU_Sej_LRR_2271 was also preliminarily tested with dog plasma samples (6 positive and 2 negative samples, confirmed by the MAT, LipL32 ELISA, and *rrs* real-time PCR assay). The protocol was modified from the rabbit hyperimmune serum assay using goat anti-dog IgG (Abcam, Cambridge, UK) conjugated with gold nanoparticles instead of goat anti-rabbit IgG. The antibody ratio to the volume of colloidal gold was 0.01 mg of antibody to 1 mL of colloidal gold.

### 2.5. Design and Development of Lateral Flow Assay to Detect Dog IgG against Leptospira

The lateral flow test strip was designed to contain 5 parts, the nitrocellulose membrane (CN95, Unisart, Goettingen, Germany), the conjugated pad (Grade 8964 Cytosep, Ahlstrom, Helsinki, Finland), the sample pad (C048, Millipore, Bedford, MA, USA), the wicking pad (Grade 470, Whatman-GE Healthcare, Uppsala, Sweden), and the backing card. Each part was mounted on the backing card in sequence with a 1 mm overlap. The LRR recombinant protein rhKU_Sej_LRR_2271 was used as a specific antigen for dog anti-leptospiral IgG detection. The rhKU_Sej_LRR_2271 was immobilized on the test line (T-line), and the rabbit anti-goat IgG (Abcam) antibody on the control line (C-line). Both lines were immobilized on a nitrocellulose membrane using paintbrushes (Seikai, China). The concentration of rhKU_Sej_LRR_2271 on the test line was tested at 200, 400, and 800 µg/mL, and the concentration of rabbit anti-goat IgG on the control line at 50, 100, and 200 µg/mL. The membrane was blocked with 1% BSA in PBS for 30 min, dried at 37 °C for 1 h, kept at 4 °C, and dried until used. Goat anti-dog IgG antibody (Abcam, Cambridge, UK) was immobilized on gold nanoparticles (40 nm) by non-covalent interaction. The antibody-to-gold nanoparticle ratio was 0.01 mg of antibody to 1 mL of colloidal gold. The colloidal gold-labeled goat anti-dog IgG in gold diluent buffer (0.02 M Na_2_HPO_4_, 1% BSA, 0.01% NaN_3_, 20% sucrose) was coated on the conjugate pad at 5 µL/cm. The running buffer kept the sample flowing through the conjugate pad, test line, and control line. A wicking pad helped to absorb excess buffer and sample, and allowed the sample to flow in one direction. Five running buffers were tested using ultrapure water, phosphate-buffered saline, pH 7.4 (PBS), 0.1 M sodium borate buffer, pH 8.5 (SBB), 0.2 M SBB, and 0.2 M SBB with 1 % Tween 20 and 0.02 % NaN_3_. For testing with dog plasma samples, strips were developed using the optimum antibody concentration, the recombinant protein, and the running buffer. Each plasma sample (5 µL) was dropped on a sample pad of strips. Then, the sample pad was immersed in the running buffer for 15 min and a result was read. A test strip that presented two red lines of control and test was interpreted as a positive result. A test strip showing only one red control line was interpreted as negative. All samples were tested in triplicate.

### 2.6. Evaluation and Validation Methods

The dog samples were also tested with serological and molecular assays to confirm the results using culture, real-time PCR, ELISA, and MAT.

#### 2.6.1. Identification of Leptospires in Dog Blood Samples by Culture

Three drops of dog blood samples were cultured in 5 mL of Ellinghausen and McCullough medium, modified by Johnson and Harris (EMJH) medium (Difco, MD, USA) in the dark at room temperature for 30–35 days. Leptospires were then detected using a darkfield microscope Olympus BH2 (Shinjuku, Tokyo, Japan) with 400× magnification.

#### 2.6.2. Molecular Analysis of rrs Real-Time PCR in Plasma Samples

The DNA of dog plasma samples was extracted by phenol: chloroform: isoamyl alcohol (PCI) assay, adapted from a previous study [34]. The plasma samples (300 µL each) were added to 9 µL of 10% formalin and the solution was kept at 4 °C for 24 h. Plasma samples were centrifuged at 15,000× *g* for 20 min at 4 °C, and pellets were kept at −20 °C until used. A total of 170 µL of lysis buffer (0.05 M EDTA, 0.05 M Tris-HCl, 0.1 M NaCl, 0.5% SDS, 50 ug/mL proteinase K) was added to the pellets. The pellets and solutions were then mixed with a handheld homogenizer and incubated at 37 °C overnight. The SDS concentration was adjusted to 1%. DNA samples were extracted by phenol: chloroform: isoamyl alcohol (25:24:1) and precipitated by absolute ethanol. The DNA pellets were resuspended in 90 µL of TE buffer and kept at −20 °C until used. Primers Lep F (GGCGGCGCGTCTTAAACATG) and Lep R (TCCCCCCATTGAGCAAGATT) specific to the 16S rRNA gene (rrs) of *Leptospira* from previous studies [38,39] were used for evaluation in this study. A total of 10 µL reaction mix volume contained 1 × iTaq Universal SYBR Green Supermix (Bio-Rad, Hercules, CA, USA), 300 nM of each primer, and 4.5 µL of a DNA sample. The real-time PCR assay was performed in a real-time PCR machine (CFX96 Touch Deep Well Real-Time PCR Detection System; Bio-Rad, Hercules, CA, USA) with initial denaturation at 95 °C for 3 min followed by 40 cycles of denaturation at 95 °C for 30 s, annealing/extension at 60 °C for 30 s. The melting curve was analyzed at 60–95 °C, at 0.5 °C increments, and at 5 s/step. The detection limits and sensitivities of Lep primers were determined using a serial dilution of leptospires of spiked plasma samples (1.16 to 1.16 × 10^7^ cells/reaction). The specificity of primers was evaluated with 24 serovars of *Leptospira* spp. and 4 strains of other bacteria, *A. viridans*, *E. coli* strain DH5α, *Salmonella* sp., and *S. aureus*.

#### 2.6.3. Detection of Anti-Leptospiral IgG in Dog Using LipL32 ELISA

The detection of anti-leptospiral IgG in dog plasma using LipL32 ELISA followed a previously published protocol [40]. Briefly, the recombinant antigen LipL32 was diluted with carbonate-bicarbonate buffer (pH 9.6), to a final concentration of 1 µg/mL, and coated on 96-well polystyrene microplates (NuncMaxiSorp, Thermo Fisher Scientific, Waltham, MD, USA) by adding 100 µL of this dilution to each well and leaving it at room temperature for 2 h. The wells were washed three times with PBST and blocked with 200 µL of 2% BSA in PBST at room temperature for 1 h. After discarding the blocking solution, dog plasma samples at dilution 1:100 in PBS with 2% BSA were added and incubated at 37 °C for 1 h. Wells were washed three times with PBST and incubated with 100 µL/well of goat anti-dog IgG-HRP (Abcam, Cambridge, UK) at a dilution of 1:10,000 at 37 °C for 1 h. The 1-StepTM Ultra TMB substrate solution (Thermo Scientific, Waltham, MA, USA) was added at 100 µL/well after being washed 3 times with PBST, and incubated for 25 min in the dark. The reactions were stopped by adding 2 M sulfuric acid (100 µL/well). Absorbance of each sample was measured at 450 nm using an ELISA reader (BioTek Synergy H1, BioTek, Winooski, VT, USA). The optimal cutoff value of LipL32 ELISA was determined using a receiver–operator curve (ROC). Samples were considered positive when the absorbance was greater than 1.700. All samples were tested in triplicate.

#### 2.6.4. Microscopic Agglutination Test (MAT)

The MAT was performed at the Veterinary College Diagnostic Center of Walailak University with the standard method. Briefly, the plasma or sera of 6 positives, as analyzed by real-time PCR, 5 positives and 5 negatives of LipL32 ELISA, and 20 vaccinated dogs were 2-fold serial diluted starting at 1:25 and incubated with 24 reference serovars of living leptospires (1 × 10^8^ to 2 × 10^8^ organisms/mL). The reactions were analyzed after 3 h incubation at room temperature by darkfield microscopy. The highest dilution of the serum sample at 50% agglutination ≥ 1:100 was considered as a positive sample. The 24 reference strains of *Leptospira* are shown in Supplementary Table S1.

### 2.7. Statistical Analysis

The relative sensitivity, specificity, and accuracy of the lateral flow assay (LFA) for the detection of anti-leptospiral IgG in dog plasma were determined in comparison to LipL32 ELISA as follows, sensitivity = a/(a + b) × 100; specificity = d/(c + d) × 100; and accuracy = [(a + d)/(a + b + c + d)] × 100, where a is the number of positive samples by both LFA and ELISA; b is the number of positive samples by ELISA but negative by LFA; c is the number of negative samples by ELISA but positive by LFA; and d is the number of negative samples by both ELISA and LFA. The associations between LFA and ELISA were determined using the chi-square test. The associations between types of dogs and the presence of leptospirosis of each detection method were also determined using the Fisher’s exact test when sample sizes were smaller than 5 [41] or the chi-square test [42]. Significance levels were observed at *p*-value < 0.05.

## 3. Results

### 3.1. Line Blot with Rabbit Hyperimmune Sera and Dog Plasma Samples

**Figure 1:**
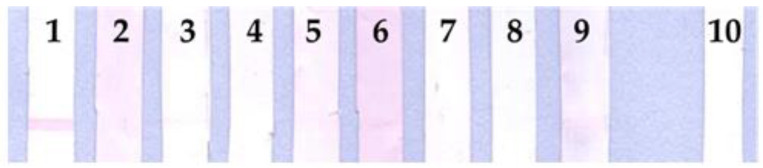
Immunoreactivities as indicated by line blot assays between the recombinant protein rhKU_Sej_LRR_2271 and rabbit hyperimmune sera. rhKU_Sej_LRR_2271 at 50 µg/mL were immobilized on nitrocellulose membrane and tasted with 9 different rabbit hyperimmune sera against *Leptospira* serovar Australis (1), Bataviae (2), Canicola (3), Hebdomadis (4), Icterohaemorrhagiae (5), Mini (6), Patoc (7), Pomona (8), Tarassovi (9), and control or non-immunized rabbit serum (10) at dilution of 1:100. Goat anti-rabbit IgG conjugated with gold nanoparticle at a dilution of 1:20 was used as the immunoreactivity color marker.

**Figure 2:**
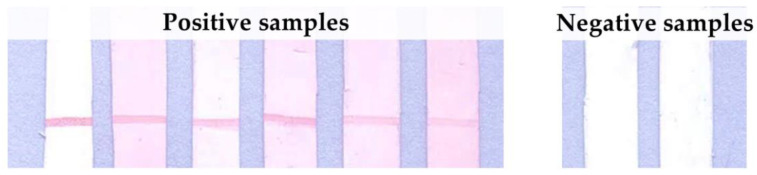
Immunoreactivities on line blot assay between rhKU_Sej_LRR_2271 and dog plasma samples. The protein rhKU_Sej_LRR_2271 at 50 µg/mL was immobilized on nitrocellulose membrane and tested with 6 plasma samples of sick dogs and 2 plasma samples of healthy dogs at dilution of 1:100. Goat anti-dog IgG conjugated with gold nanoparticle at a dilution of 1:20 was used as the immunoreactivity color marker.

The recombinant proteins were tested with nine rabbit hyperimmune sera, which are summarized in Table 1. The protein rhKU_Sej_LRR_2271 was recognized by the rabbit hyperimmune serum against *L. interrogans* serovar Australis (Figure 1), while LipL32 was recognized by eight rabbit hyperimmune sera against *Leptospira* serovar Australis, Bataviae, Canicola, Hebdomadis, Iceteroheamorrhagiae, Mini, Pomona, and Tarassovi (The data was shown in Figure 1B of a previous study [40]). Both rhKU_Sej_LRR_2271 and LipL32 showed negative results with the control rabbit serum. The rhKU_Sej_LRR_2271 also demonstrated positive results with the six positive plasma samples of dogs which had signs and symptoms of leptospirosis and negative results with the plasma samples of healthy dogs (Figure 2). All dog plasma samples were confirmed as either positive or negative for *Leptospira* by the real-time PCR assay, LipL32 ELISA, and MAT.

### 3.2. Leptospires Detection in Blood and Plasma Samples by Culture Method and Real-Time PCR

**Figure 3:**
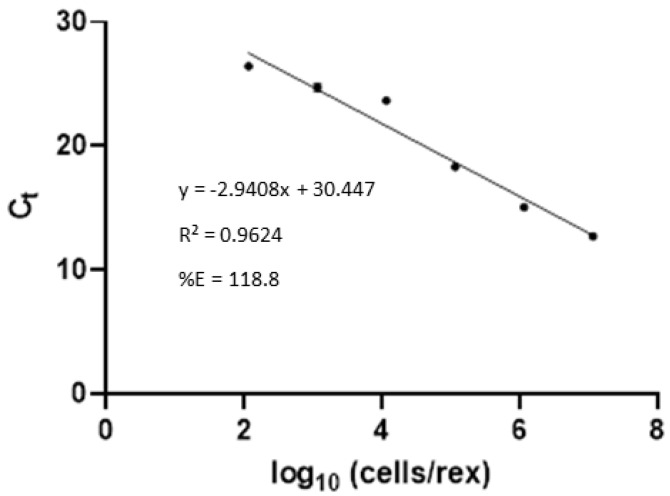
Real-time PCR standard curve of Lep primers with spike plasma samples extracted by PCI assay was used for analytical sensitivity of the real-time PCR assay. The sensitivity of PCI extracted spike plasma sample were approximately 116 cells/reaction.

All dog blood samples produced negative results by the culture assay. For the real-time PCR assay, the standard curve of PCI extracted spiked plasma samples with Lep primers are shown in Figure 3. The amplification sensitivity was determined to 116 cells/reaction, and the amplification efficiency was 118.8%. The Lep primers had high specificity to *Leptospira* spp. and gave positive results with all 24 serovars of *Leptospira* spp. (Supplementary Table S1) and gave negative results with *A. viridans*, *E. coli* strain DH5α, *Salmonella* sp., and *S. aureus*. Any sample with a Ct value below 30 cycles was considered positive. Only six of nine symptomatic dogs showed positive results with the real-time PCR assay.

### 3.3. Anti-Leptospiral IgG Detection Using LipL32 ELISA

The dog plasma samples detected the anti-leptospiral IgG using LipL32-base IgG ELISA. In a total of 184 dogs, 50 dogs presented positive results, whereas 134 dogs showed negative results with the LipL32 ELISA (Table 2). A total of six of the nine symptomatic dogs which showed positive results with the real-time PCR assay also gave positive results with LipL32 ELISA. The results of the ELISA assay were used for evaluation of the rhKU_Sej_LRR_2271-based LFA.

### 3.4. Lateral Flow Optimization

**Figure 4:**
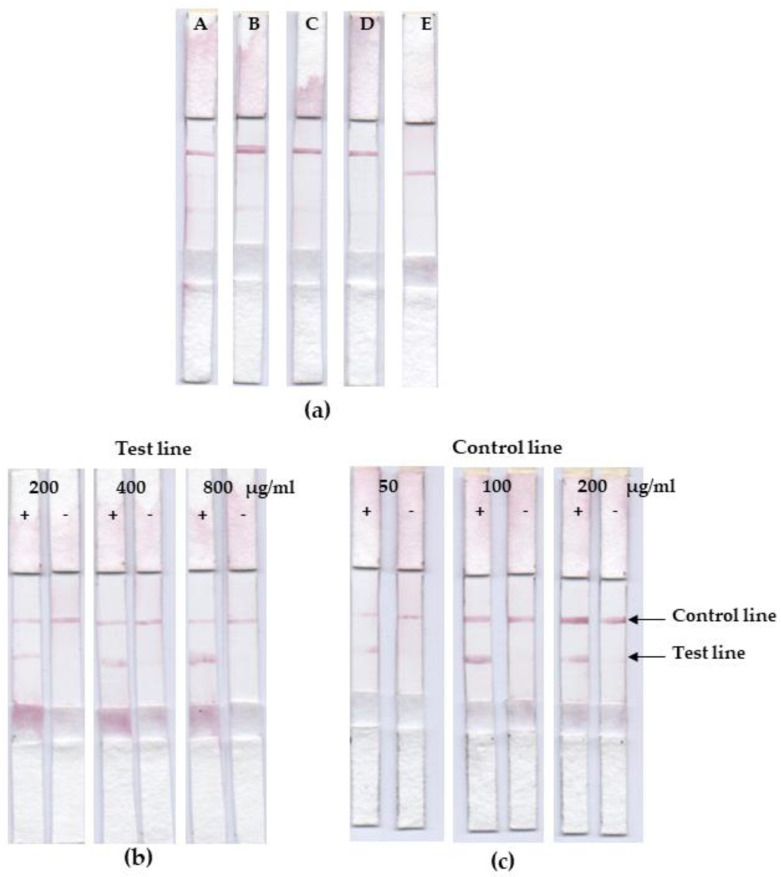
Optimization of running buffers (**a**), concentration of rhKU_Sej_LRR_2271 on the test line (**b**), and rabbit anti-goat IgG antibody on the control line (**c**). Strips with 400 µg/mL of rhKU_Sej_LRR_2271 and 100 µg/mL of rabbit anti-goat IgG antibody were tested with ultrapure water (A), ultrapure water, PBS (B), 0.1 M SBB (C), 0.2 M SBB (D), and 0.2 M SBB with 1% Tween20 and 0.02% NaN_3_ (E). The concentration of rhKU_Sej_LRR_2271 on the test line was varied at 200, 400, and 800 µg/mL, whereas the concentration of rabbit anti-goat IgG antibody was 100 µg/mL (**b**). The concentration of rabbit anti-goat IgG antibody on the control line was varied at 50, 100, and 200 µg/mL, whereas the concentration of rhKU_Sej_LRR_2271 on the test line was 800 µg/mL (**c**). The strips were tested with positive and negative samples. The running buffer was 0.2 M SBB with 1% Tween20 and 0.02% NaN_3_.

The strips were developed for dog anti-leptospiral IgG detection using the LRR recombinant protein, rhKU_Sej_LRR_2271 on the test line, rabbit anti-goat IgG on the control line, and gold nanoparticles conjugated goat anti-dog IgG on the conjugate pad. The running buffer flowed through a sample on the sample pad, and goat anti-dog IgG conjugated gold nanoparticles flowed through the conjugated pad, test and control line on the membrane, and the wicking pad, respectively. A positive result was represented by the presence of red lines at the test and control line positions. A negative result was represented with only a red line at the control line position (Figure S1). In this study, we optimized the running buffer types, the rabbit anti-goat IgG antibody concentration on the control line, and the rhKU_Sej_LRR_2271 concentration on the test line. The optimal running buffer without giving false positives was 0.2 M SBB with 1 % Tween20 and 0.02 % NaN_3_ (Figure 4a). The optimum concentration of the rhKU_Sej_LRR_2271 and rabbit anti-goat IgG antibody were 800 and 100 µg/mL, respectively (Figure 4b,c).

### 3.5. Assessment on Leptospiral IgG in Dog’s Plasma Samples

Out of 184 dogs, 59 dogs presented positive results, whereas 125 dogs exhibited negative results with the lateral flow assay. In total, seven of nine symptomatic dogs showed a positive result with the lateral flow assay. Table 2 shows the number of positive and negative dogs with the rhKU_Sej_LRR_2271 lateral flow assay (LFA) compared with LipL32 ELISA. The sensitivity, specificity, and accuracy of the rhKU_Sej_LRR_2271 lateral flow assay (LFA) for the detection of anti-leptospiral IgG in dog plasma in comparison with LipL32 ELISA were 70.0, 82.1, and 78.8 %, respectively. The *p*-value of the chi-square test was less than 0.00001 demonstrating that there was a significant association between rhKU_Sej_LRR_2271-based LFA and LipL32 ELISA.

### 3.6. Validation with MAT

The six symptomatic dogs that were analyzed as positive by the real-time PCR were also identified as positive by the MAT, the LipL32 ELISA, and the rhKU_Sej_LRR_2271-based lateral flow assay (Table 3). In addition, 19 vaccinated dog sera were also shown to be positive with the MAT and the LipL32 ELISA. All the vaccinated dogs tested as not immunopositive with the rhKU_Sej_LRR_2271-based LFA. A total of five of the positive samples by the LipL32 ELISA also presented positive results with the MAT and the LFA (Table 3), whereas five of the negative samples by LipL32 ELISA also indicated negative with the MAT (The data was shown in Table 5 of a previous study [40]) and LFA.

### 3.7. Association between Dog Types and Leptospirosis

The number of positive and negative samples of each type of dog analyzed by each method are shown in Table 4. All six positive symptomatic dogs that were positive by the real-time PCR assay, ELISA, and LFA were family dogs. The association between dog types (family and stray dogs) and leptospirosis was analyzed using the Fisher’s exact or chi-square tests. There was no significant association between dog types and the presence of leptospirosis when using culture, LipL32 ELISA, and rhKU_Sej_LRR_2271-based LFA with *p*-values 1.000, 0.217, and 0.102, respectively. Interestingly, only the real-time PCR assay demonstrated a significant correlation between dog types and the presence of leptospirosis with *p*-values < 0.05. The data are displayed in Table 4.

## 4. Discussion

This study aimed to develop a lateral flow test strip using the recombinant LRR protein rhKU_Sej_LRR_2271. The samples were distinguished between positive and negative results using the bacteria culture and the real-time PCR assay to detect leptospires, and the LipL32-based IgG ELISA for immunoreactive detection. The lateral flow results were evaluated with LipL32 ELISA. The MAT was used as a validation method. The specificity of the protein rhKU_Sej_LRR_2271 was performed by using rabbit antisera against *Leptospira* spp.

The recombinant protein rhKU_Sej_LRR_2271 was obtained from the cloned LRR gene of *L. borgpetersenii* serovar Sejroe, an ortholog with the gene encoding LBJ_2271 of *L. borgpetersenii* serovar hardjo-bovis strain JB197 [36]. The in silico protein characterization revealed that the LBJ_2271 protein consists of four LRR motifs, a signal peptide, an N-glycosylation site, and two transmembrane domains [35]. The rhKU_Sej_LRR_2271 protein was 98.6% identical to LBJ_2271. Moreover, LBJ_2271 and rhKU_Sej_LRR_2271 contained many immunogenic epitopes with IC_50_ scores equal to or lower than those of well-known antigens such as LipL32 [35,36]. In addition, the rhKU_Sej_LRR_2271 protein contained T-cell epitopes and was proven to induce both humoral and cell-mediated immune responses in a rabbit model [37]. Accordingly, we used rhKU_Sej_LRR_2271 to develop a lateral flow test strip for the detection of anti-leptospiral IgG in canines.

For the line blot assay, the rhKU_Sej_LRR_2271 protein was specifically detected only with rabbit hyperimmune serum against *L. interrogans* serovar Australis, and failed to detect rabbit hyperimmune sera against *L. interrogans* serovar Icterohaemorrhagiae, Canicola, Bataviae, Pomona, and *L. borgpetersenii* serovar Mini. However, it demonstrated immunoreactivity to the plasma of six symptomatic dogs, which were confirmed as *Leptospira*-positive by the real-time PCR, ELISA, and MAT. The positive MAT samples were against the serovars Autumnalis, Canicola, Icterohaemorrhagiae, Sejroe, Tarassovi, and Patoc. Moreover, previous study has shown that the rhKU_Sej_LRR_2271 protein was immunoreactive to rabbit hyperimmune serum against the *L. borgpetersenii* serovar, Sejroe and Ballum, and mouse hyperimmune sera against the *L. borgpetersenii* serovar Sejroe, and the *L. interrogans* serovars, Bratislava and Icterohaemorrhagiae [36]. The amino sequence alignment result of rhKU_Sej_LRR_2271 (Genbank: AFV46188.1) displayed a high similarity (over 77%) to several serovars of *Leptospira* spp. Such as Ballum, Javanica, Mini, Sejroe, Pomona, Grippotyphosa, Autumnalis, Canicola, Bataviae, Panama, Icterohaemorrhagiae, Australis, Pyrogenes, and Bratislava. The data of alignment are shown in Supplementary Data S1. The rhKU_Sej_LRR_2271 had no immunoreactivity to rabbit hyperimmune sera of the serovars Hebdomadis, Tarassovi, and Patoc. This might be because the sequence of those serovars has no resemblance to rhKU_Sej_LRR_2271 (Supplementary Data S1). As is known, the severity of signs and symptoms after infection depends on the serotypes of *Leptospira* and its hosts [2,43]. The specific pattern recognition receptors (PRRs) such as Toll-like and NOD-like receptors, the innate immune response of hosts, molecular mimicry, and the escape of recognition of leptospires might be important factors in the differences of their immune responses [32,43,44].

Unsurprisingly, LipL32 was detected by all rabbit hyperimmune sera against *Leptospira* spp., except serovar Patoc, which is a saprophytic strain. LipL32 is the most abundant outer membrane protein (OMP) of leptospiral pathogenic strains. Therefore, LipL32 has been used for the diagnosis of leptospirosis in many studies [24,25,26,27,28,29] and is also used in commercial test kits [45]. In addition, the LipL32-based IgG ELISA has been proven to have sensitivity, specificity, and accuracy comparable to those of the gold standard MAT [28,45,46,47]. Accordingly, the LipL32-based IgG ELISA was used to evaluate the rhKU_Sej_LRR_2271 lateral flow assay in this study.

For the detection of leptospires in blood or plasma samples, no dog gave a positive result with the culture method, and only six dogs (3.26%) were positive when analyzed by real-time PCR. In comparison, the methods for detecting anti-leptospiral IgG, ELISA and LFA, gave positive results for 50 (27.17%) and 59 (32.07%) dogs, respectively. The results of this study are therefore consistent with several previous studies that demonstrated that the methods for the detection of leptospiral antibodies (MAT, LAT, ELISA) give more positive results than molecular diagnostic assays, and the culture method, respectively [5,6,48,49,50]. The prevalence of Thai dogs that presented antibodies against *Leptospira* by the microscopic agglutination test (MAT), latex agglutination test (LAT), and ELISA varied from 4.3 to 89.1% [4,5,7,8,9,10,51]. Whereas the prevalence of the culture method and molecular diagnosis was 0.37–6.89%, and 0.54–10.3%, respectively [5,6,48]. Direct culture of leptospires from blood or urine is highly specific, but it is technically difficult and the sensitivity level is quite low [52,53]. Molecular diagnostic tools, such as PCR and real-time PCR assays present a high sensitivity in the early stages of the infection. The leptospires can be discovered in unvaccinated-dog blood after 1–10 days of infection with *L. interrogans* serovar Canicola and after 1-6 days of infection with *L. interrogans* serovar Icterohaemorrhagiae [54]. It is recommended that the whole blood sample is submitted for PCR within the first 10 days of illness, or the leptospiremia phase, while the urine sample is recommended for PCR test after the first week of illness, or the leptospiruria phase [53]. One study showed that the PCR test with blood samples gave the sensitivity up to 86% in the first 6 days of infection, but the sensitivity was significantly decreased to 34% after 7 days of infection [53,55]. ELISA can be designed to detect IgM and IgG in dogs. IgG is produced after 1 to 3 weeks of infection, while IgM can be detected during the first week of infection [53]. The MAT is the gold standard method for the detection of leptospirosis. The MAT has low sensitivity in the initial phase of infection, its results improve after 1 week, peak at 3–4 weeks, and then remain positive for many months [53]. However, serological tests, such as the MAT cannot differentiate between antibodies from natural infection and antibodies from vaccination [53,56]. Therefore, the healthy dogs that were showing as positive with the LipL32-based IgG ELISA and the rhKU_Sej_LRR_2271 lateral flow assay may have recovered from infection or may have been vaccinated.

The false negative results of the rhKU_Sej_LRR_2271 LFA might result from the fact that the rhKU_Sej_LRR_2271 protein had less cross immunoreactive than LipL32 or those vaccinated dogs gave a positive result to the LipL32 ELISA. However, the chi-square statistical analysis indicated that there is a significant correlation between the LipL32-based IgG ELISA and the rhKU_Sej_LRR_2271 lateral flow assay. For further research, the rhKU_Sej_LRR_2271 protein should be tested for specificity with antisera against other species of *Leptospira* because testing with only nine prevalent serovars in the Thai dog population cannot indicate cross immunoreactivities of the protein to all 300 serovars.

## 5. Conclusions

The rhKU_Sej_LRR_2271 protein and the rhKU_Sej_LRR_2271 lateral flow test strips both have the potential to detect anti-leptospiral IgG when compared with the LipL32 ELISA using Thai canine plasma samples. The protein should be tested with additional serovars of antisera for a better understanding of the specificity of this protein.

## Figures and Tables

**Table 1:** Rabbit hyperimmune sera against *Leptospira* spp. in this study.

Species	Serogroup	Serovar	Strain
*L. interrogans*	Australis	Australis	Ballico
	Bataviae	Bataviae	Swart
	Canicola	Canicola	Hond Utrecht lV
	Hebdomadis	Hebdomadis	Hebdomadis
	Icterohaemorrhagiae	Icterohaemorrhagiae	Ictero l
	Pomona	Pomona	Pomona
*L. biflexa*	Semaranga	Patoc	Patoc l
*L. borgpetersenii*	Mini	Mini	Sari
	Tarassovi	Tarassovi	Perepelitsin

**Table 2:** rhKU_Sej_LRR_2271 lateral flow assay (LFA) compared with LipL32 ELISA.

	ELISA Positive (Dogs)	ELISA Negative (Dogs)	Total (Dogs)
rhKU_Sej_LRR_2271 LFA positive	35	24	59
rhKU_Sej_LRR_2271 LFA negative	15	110	125
Total (dogs)	50	134	184

The *p*-value was < 0.00001, analyzed by the chi-square test (significant at *p*-value <0.05).

**Table 3:** The validation of rhKU_Sej_LRR_2271-based LFA, real-time PCR, and LipL32 ELISA with MAT results from known true positive dog sera.

Methods		Results (Number of Dogs with Positive Results)		
		Real-Time PCR Positive (6)	LipL32 ELISA Positive (5)	Vaccinated Dogs (20)
MAT (serovar with titer ≥1:100)	Autumnalis	1	-	-
	Bratislava	-	1	8
	Canicola	4	2	11
	Icterohaemorrhagiae	3	1	13 *
	Pomona	-	-	6
	Ballum	-	1	-
	Sejroe	5	4	-
	Tarassovi	4	1	-
	Grippotyphosa	-	-	3
	Patoc	3	3	-
LipL32 ELISA		6	5	19
Culture method		0	0	0
rhKU_Sej_LRR_2271-based LFA		6	5	0

* 3 dogs with MAT titer ≥1:400 to serovar Icterohaemorrhagiae.

**Table 4:** The number of positive and negative samples of each type of dogs analyzed by culture, real-time PCR, ELISA, and lateral flow assay.

Method	Dog Type	Result (Dogs)		*p*-Value
		Positive	Negative	
Culture	stray dog	0	91	1.000 *
	family dog	0	93	
Real-time PCR	stray dog	0	91	0.029 *
	family dog	6	87	
ELISA	stray dog	21	70	0.217
	family dog	29	64	
Lateral flow	stray dog	24	67	0.102
	family dog	35	58	

* *p*-value was analyzed by the Fisher’s exact test.

## Data Availability

The datasets generated during and/or analyzed during the current study are available from the corresponding author upon request.

## Supplementary Materials

The following supporting information can be downloaded at: https://www.mdpi.com/article/10.3390/tropicalmed7120427/s1; Supplementary Table S1: The reference serovars and strains of *Leptospira* spp. used in the real-time PCR and MAT.; Supplementary Figure S1: Schematic representation of the different parts of the rhKU_Sej_LRR_2271 lateral flow strip and representation of the results after testing with positive and negative dog plasma samples.; Supplementary Data S1: Amino sequence alignment of rhKU_Sej_LRR_2271.
