# Supplementary material for: Leptospiral Leucine-Rich Repeat Protein-Based Lateral Flow for Assessment of Canine Leptospiral Immunoglobulin G
Source: Trop Med Infect Dis. 2022 Dec 10;7(12):427. doi: 10.3390/tropicalmed7120427 (PMC9787837; doi:10.3390/tropicalmed7120427)
Supplement: Supplementary file 1 [file tropicalmed-07-00427-s001.zip › tropicalmed-2073363-Supplementary.pdf]

**Supplementary data**

# Evaluation of a Leucine-rich-repeat protein-based lateral flow assay for detection of anti-leptospiral IgG in Thai canine

**Sineenat Sripattanakul [1,2,†], Kanpapat Boonchuay [3,4,†], Teerasak Prapong [2], Worawidh Wajjwalku [3,4], Gerd Katzenmeier [3,4], Dietmar Haltrich [5], Ratchanee Hongprayoon [1] and Siriwan Prapong [1,2,*]**

**Supplementary Table S1:** The reference serovars and strains of *Leptospira* spp. utilized in real-time PCR and MAT.

| **Genospecies** | **Serovars[a]** | **Strain[a]** |
|---|---|---|
| *L. interrogans* | Autumnalis | Akiyami |
| | Bataviae | A Swart |
| | Bratislava | Jez Bratislava |
| | Canicola | Houd UtrechtIV |
| | Djasiman | Djasiman |
| | Hebdomadis | Hebdomadis |
| | Icterohaemorrhagiae | RGA |
| | Pomona | Pomona |
| | Pyrogenes | Salinem |
| *L. noguchii* | Louisiana | LSU 1945 |
| | Panama | CZ 214 K |
| *L. borgpetersenii* | Ballum | MUS 127 |
| | Javanica | Veldrat Batavia 46 |
| | Mini | Sari |
| | Sejroe | M 84 |
| | Tarassovi | Perepelitsin |
| *L. santarosai* | Shermani | 1342 K |
| *L. weilii* | Celledoni | Celledoni |
| | Manhao | L 105 |
| | Sarmin | Sarmin |
| *L. kirschneri* | Cynopteri | 3522 C |
| | Grippotyphosa | Moskva V |
| *L. meyeri* | Ranarum | ICF |
| *L. biflexa* | Patoc | Patoc 1 |

[a] The serovars and strains of *Leptospira* were kindly provided by the National Institute of Animal Health, department of Livestock Department, Thailand.

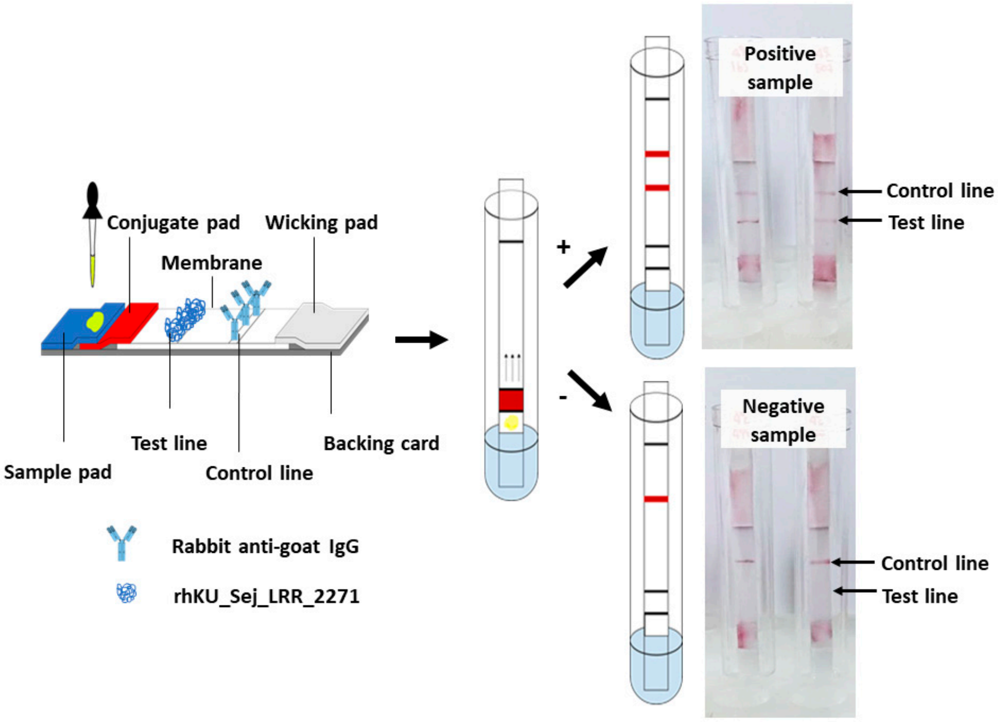


**Supplementary Figure S1:** Schematic representation of the different parts of the rhKU_Sej_LRR_2271 lateral flow strip and representation of the results after testing with positive and negative dog plasma samples. The test line contained 800 µg/ml of rhKU_Sej_LRR_2271 and the control line 100 µg/ml of rabbit anti-goat IgG. The conjugate pad contained of gold nanoparticle conjugated goat anti-dog IgG. The running buffer was 0.2 M SBB with 1 % Tween20 and 0.02 % $NaN_3$.

**Supplementary data S1:** Amino sequence alignment of rhKU_Sej_LRR_2271

BLASTP 2.13.0+

Reference: Stephen F. Altschul, Thomas L. Madden, Alejandro A. Schaffer, Jinghui Zhang, Zheng Zhang, Webb Miller, and David J. Lipman (1997), "Gapped BLAST and PSI-BLAST: a new generation of protein database search programs", Nucleic Acids Res. 25:3389-3402.

Reference for compositional score matrix adjustment: Stephen F. Altschul, John C. Wootton, E. Michael Gertz, Richa Agarwala, Aleksandr Morgulis, Alejandro A. Schaffer, and Yi-Kuo Yu (2005) "Protein database searches using compositionally adjusted substitution matrices", FEBS J. 272:5101-5109.

RID: PN3VPV8W013

Database: All non-redundant GenBank CDS translations+PDB+SwissProt+PIR+PRF excluding environmental samples from WGS projects

512,909,985 sequences; 200,317,139,153 total letters

Query= AFV46188.1 leucine-rich repeat protein, partial [Leptospira borgpetersenii serovar Sejroe]

Length=203

| Description | Max Score | Total Score | Query Cover | E value | Per. Ident | Accession |
|---|---|---|---|---|---|---|
| leucine-rich repeat protein [Leptospira borgpetersenii serovar Ballum] | 358 | 358 | 100% | 6.00E-124 | 100.00% | AFV46186.1 |
| leucine rich repeat protein [Leptospira borgpetersenii serovar Ballum] | 359 | 359 | 100% | 8.00E-124 | 100.00% | ALO26640.1 |
| leucine-rich repeat domain-containing protein [Leptospira] | 358 | 358 | 100% | 1.00E-123 | 100.00% | WP_002731979.1 |
| leucine-rich repeat protein [Leptospira borgpetersenii serovar Javanica] | 354 | 354 | 100% | 3.00E-122 | 99.01% | AFV46185.1 |
| leucine rich repeat protein [Leptospira borgpetersenii serovar Pomona str. 200901868] | 354 | 354 | 100% | 7.00E-122 | 99.01% | EMO62774.1 |
| leucine-rich repeat domain-containing protein [Leptospira borgpetersenii] | 352 | 352 | 100% | 4.00E-121 | 98.52% | WP_011669694.1 |
| hypothetical protein [Leptospira mayottensis] | 331 | 331 | 100% | 8.00E-113 | 91.63% | WP_002745429.1 |
| hypothetical protein [Leptospira weilii] | 330 | 330 | 100% | 1.00E-112 | 90.15% | WP_235596156.1 |
| leucine rich repeat protein [Leptospira sp. P2653] | 328 | 328 | 100% | 1.00E-111 | 91.13% | EMJ66028.1 |
| leucine-rich repeat domain-containing protein [Leptospira] | 328 | 328 | 100% | 1.00E-111 | 91.13% | WP_004496751.1 |
| leucine-rich repeat domain-containing protein [Leptospira weilii] | 326 | 326 | 100% | 8.00E-111 | 90.64% | WP_061219130.1 |
| leucine rich repeat protein [Leptospira weilii str. UI 13098] | 325 | 325 | 100% | 1.00E-110 | 90.64% | EMN91031.1 |
| leucine-rich repeat domain-containing protein [Leptospira weilii] | 325 | 325 | 100% | 2.00E-110 | 90.64% | WP_026054684.1 |
| leucine-rich repeat domain-containing protein [Leptospira weilii] | 322 | 322 | 99% | 2.00E-109 | 90.59% | WP_061217745.1 |
| leucine rich repeat protein [Leptospira alexanderi serovar Manhao 3 str. L 60] | 312 | 312 | 100% | 2.00E-105 | 86.70% | EQA64549.1 |
| hypothetical protein [Leptospira alexanderi] | 311 | 311 | 100% | 3.00E-105 | 86.70% | WP_039940928.1 |
| hypothetical protein [Leptospira alexanderi] | 309 | 309 | 100% | 4.00E-104 | 86.21% | WP_078124592.1 |
| leucine-rich repeat domain-containing protein [Leptospira santarosai] | 307 | 307 | 100% | 2.00E-103 | 86.34% | WP_004466481.1 |
| leucine-rich repeat domain-containing protein [Leptospira santarosai] | 306 | 306 | 100% | 4.00E-103 | 85.85% | WP_046692451.1 |

| Description | Max Score | Total Score | Query Cover | E value | Per. Ident | Accession |
|---|---|---|---|---|---|---|
| hypothetical protein LSS_15741 [Leptospira santarosai serovar Shermani str. LT 821] | 305 | 305 | 100% | 1.00E-102 | 85.85% | EKT85788.2 |
| leucine-rich repeat domain-containing protein [Leptospira] | 305 | 305 | 100% | 2.00E-102 | 85.85% | WP_004471631.1 |
| leucine-rich repeat domain-containing protein [Leptospira santarosai] | 305 | 305 | 100% | 2.00E-102 | 85.85% | WP_061216203.1 |
| leucine-rich repeat domain-containing protein [Leptospira santarosai] | 304 | 304 | 100% | 3.00E-102 | 85.85% | WP_004492778.1 |
| leucine rich repeat protein [Leptospira santarosai str. CBC1416] | 305 | 305 | 100% | 3.00E-102 | 85.37% | EMO58507.1 |
| leucine-rich repeat domain-containing protein [Leptospira santarosai] | 304 | 304 | 100% | 4.00E-102 | 85.37% | WP_004468285.1 |
| leucine-rich repeat domain-containing protein [Leptospira santarosai] | 303 | 303 | 100% | 6.00E-102 | 84.88% | WP_004486481.1 |
| leucine-rich repeat domain-containing protein [Leptospira santarosai] | 303 | 303 | 100% | 8.00E-102 | 85.37% | WP_004473752.1 |
| leucine-rich repeat domain-containing protein [Leptospira santarosai] | 302 | 302 | 100% | 2.00E-101 | 84.88% | WP_004476114.1 |
| leucine rich repeat protein [Leptospira weilii serovar Topaz str. LT2116] | 294 | 294 | 91% | 1.00E-98 | 90.27% | EMF79993.1 |
| leucine rich repeat protein [Leptospira weilii str. Ecochallenge] | 290 | 290 | 91% | 4.00E-97 | 90.27% | EMY13797.1 |
| leucine-rich repeat domain-containing protein [Leptospira barantonii] | 289 | 289 | 96% | 4.00E-96 | 84.62% | WP_100761136.1 |
| leucine-rich repeat domain-containing protein [Leptospira kmetyi] | 280 | 280 | 96% | 1.00E-92 | 82.05% | WP_123179347.1 |
| leucine-rich repeat domain-containing protein [Leptospira kmetyi] | 280 | 280 | 96% | 1.00E-92 | 82.56% | WP_100755326.1 |
| leucine-rich repeat domain-containing protein [Leptospira kmetyi] | 279 | 279 | 96% | 3.00E-92 | 82.05% | WP_135607718.1 |
| leucine-rich repeat domain-containing protein [Leptospira alstonii] | 278 | 278 | 96% | 6.00E-92 | 83.59% | WP_020775386.1 |
| leucine-rich repeat domain-containing protein [Leptospira kmetyi] | 278 | 278 | 96% | 6.00E-92 | 82.05% | WP_010574016.1 |

| Description | Max Score | Total Score | Query Cover | E value | Per. Ident | Accession |
|---|---|---|---|---|---|---|
| leucine-rich repeat domain-containing protein [Leptospira kmetyi] | 278 | 278 | 96% | 7.00E-92 | 81.54% | WP_135653950.1 |
| leucine-rich repeat domain-containing protein [Leptospira kmetyi] | 278 | 278 | 96% | 7.00E-92 | 81.54% | WP_100738364.1 |
| hypothetical protein [Leptospira yasudae] | 277 | 277 | 95% | 1.00E-91 | 81.87% | MBW0432169.1 |
| hypothetical protein EHR10_00815 [Leptospira yasudae] | 277 | 277 | 95% | 2.00E-91 | 81.87% | TGN02502.1 |
| hypothetical protein [Leptospira yasudae] | 276 | 276 | 95% | 2.00E-91 | 82.38% | WP_118964127.1 |
| leucine-rich repeat domain-containing protein [Leptospira alstonii] | 276 | 276 | 96% | 3.00E-91 | 83.08% | WP_061249228.1 |
| leucine rich repeat protein [Leptospira weilii serovar Ranarum str. ICFT] | 277 | 277 | 100% | 4.00E-91 | 78.05% | EMY77189.1 |
| hypothetical protein [Leptospira sanjuanensis] | 276 | 276 | 95% | 4.00E-91 | 81.87% | WP_238730679.1 |
| hypothetical protein [Leptospira sanjuanensis] | 276 | 276 | 95% | 6.00E-91 | 81.87% | WP_238747060.1 |
| hypothetical protein [Leptospira yasudae] | 275 | 275 | 99% | 9.00E-91 | 79.70% | WP_135574399.1 |
| hypothetical protein [Leptospira yasudae] | 273 | 273 | 95% | 7.00E-90 | 81.35% | WP_135581985.1 |
| hypothetical protein [Leptospira yasudae] | 271 | 271 | 95% | 2.00E-89 | 80.83% | WP_118956310.1 |
| hypothetical protein [Leptospira kirschneri] | 270 | 270 | 95% | 2.00E-88 | 81.44% | WP_004767299.1 |
| hypothetical protein [Leptospira kirschneri] | 270 | 270 | 95% | 2.00E-88 | 81.44% | WP_004759233.1 |
| hypothetical protein [Leptospira] | 269 | 269 | 95% | 3.00E-88 | 80.93% | WP_004750770.1 |
| hypothetical protein [Leptospira kirschneri] | 269 | 269 | 95% | 3.00E-88 | 80.93% | WP_004779378.1 |
| hypothetical protein [Leptospira kirschneri] | 268 | 268 | 95% | 4.00E-88 | 80.93% | WP_016752856.1 |
| hypothetical protein [Leptospira kirschneri] | 268 | 268 | 95% | 7.00E-88 | 80.93% | WP_020778543.1 |
| hypothetical protein [Leptospira kirschneri] | 268 | 268 | 95% | 1.00E-87 | 80.41% | WP_082292870.1 |
| hypothetical protein [Leptospira kirschneri] | 267 | 267 | 95% | 2.00E-87 | 80.41% | WP_004768364.1 |
| hypothetical protein [Leptospira kirschneri] | 266 | 266 | 95% | 2.00E-87 | 80.93% | WP_004760926.1 |
| hypothetical protein [Leptospira tipperaryensis] | 261 | 261 | 95% | 4.00E-85 | 78.24% | WP_069608131.1 |
| hypothetical protein [Leptospira ainlahdjerensis] | 261 | 261 | 95% | 4.00E-85 | 77.72% | WP_205279762.1 |
| hypothetical protein [Leptospira ainazelensis] | 258 | 258 | 95% | 5.00E-84 | 77.20% | WP_232371773.1 |
| hypothetical protein [Leptospira ainazelensis] | 258 | 258 | 95% | 6.00E-84 | 77.20% | MBM9502464.1 |
| hypothetical protein [Leptospira noguchii] | 255 | 255 | 99% | 7.00E-83 | 78.22% | WP_004443975.1 |

| Description | Max Score | Total Score | Query Cover | E value | Per. Ident | Accession |
|---|---|---|---|---|---|---|
| hypothetical protein [Leptospira noguchii] | 254 | 254 | 95% | 1.00E-82 | 80.93% | WP_004449517.1 |
| hypothetical protein [Leptospira noguchii] | 254 | 254 | 95% | 2.00E-82 | 80.93% | WP_004422093.1 |
| hypothetical protein [Leptospira noguchii] | 254 | 254 | 95% | 2.00E-82 | 80.93% | WP_004455400.1 |
| hypothetical protein [Leptospira noguchii] | 254 | 254 | 95% | 3.00E-82 | 80.41% | WP_243817925.1 |
| hypothetical protein [Leptospira noguchii] | 254 | 254 | 95% | 3.00E-82 | 80.41% | WP_053522720.1 |
| hypothetical protein [Leptospira noguchii] | 253 | 253 | 99% | 4.00E-82 | 77.72% | WP_004452014.1 |
| hypothetical protein [Leptospira noguchii] | 253 | 253 | 99% | 5.00E-82 | 77.72% | WP_243827912.1 |
| hypothetical protein [Leptospira noguchii] | 253 | 253 | 99% | 8.00E-82 | 77.72% | WP_061233070.1 |
| hypothetical protein [Leptospira interrogans] | 252 | 252 | 95% | 1.00E-81 | 80.93% | WP_017852233.1 |
| hypothetical protein [Leptospira interrogans] | 251 | 251 | 95% | 3.00E-81 | 80.93% | WP_061241709.1 |
| hypothetical protein [Leptospira interrogans] | 251 | 251 | 95% | 3.00E-81 | 80.93% | WP_001010180.1 |
| hypothetical protein [Leptospira] | 251 | 251 | 95% | 3.00E-81 | 80.93% | WP_001011126.1 |
| hypothetical protein [Leptospira stimsonii] | 251 | 251 | 96% | 4.00E-81 | 74.36% | WP_118981207.1 |
| hypothetical protein [Leptospira noguchii] | 251 | 251 | 95% | 4.00E-81 | 79.38% | WP_004427427.1 |
| hypothetical protein [Leptospira noguchii] | 251 | 251 | 99% | 5.00E-81 | 77.23% | WP_004438578.1 |
| leucine rich repeat protein [Leptospira interrogans str. L1207] | 250 | 250 | 95% | 6.00E-81 | 80.41% | EMN50877.1 |
| hypothetical protein [Leptospira] | 250 | 250 | 95% | 7.00E-81 | 80.41% | WP_001010181.1 |
| hypothetical protein [Leptospira interrogans] | 250 | 250 | 95% | 8.00E-81 | 80.41% | WP_001010177.1 |
| hypothetical protein [Leptospira noguchii] | 250 | 250 | 95% | 1.00E-80 | 79.90% | WP_002177142.1 |
| hypothetical protein [Leptospira adleri] | 249 | 249 | 95% | 1.00E-80 | 74.61% | WP_100785841.1 |
| hypothetical protein [Leptospira adleri] | 249 | 249 | 95% | 2.00E-80 | 74.61% | WP_135691680.1 |
| hypothetical protein CH379_21015 [Leptospira ellisii] | 248 | 248 | 98% | 4.00E-80 | 71.50% | PJZ91020.1 |
| hypothetical protein [Leptospira interrogans] | 248 | 248 | 95% | 5.00E-80 | 79.90% | WP_002188945.1 |
| hypothetical protein [Leptospira stimsonii] | 248 | 248 | 95% | 6.00E-80 | 75.13% | WP_118970865.1 |
| hypothetical protein [Leptospira ellisii] | 246 | 246 | 95% | 2.00E-79 | 73.20% | WP_243399589.1 |
| leucine-rich repeat domain-containing protein [Leptospira gomenensis] | 244 | 244 | 95% | 2.00E-78 | 70.47% | WP_135589926.1 |
| hypothetical protein [Leptospira interrogans] | 232 | 232 | 95% | 1.00E-73 | 80.41% | WP_250180949.1 |
| hypothetical protein [Leptospira interrogans] | 231 | 231 | 95% | 2.00E-73 | 80.41% | WP_061272825.1 |

| Description | Max Score | Total Score | Query Cover | E value | Per. Ident | Accession |
|---|---|---|---|---|---|---|
| hypothetical protein [Leptospira interrogans] | 231 | 231 | 95% | 2.00E-73 | 80.41% | WP_002127204.1 |
| hypothetical protein [Leptospira interrogans] | 231 | 231 | 95% | 2.00E-73 | 79.38% | WP_061286495.1 |
| hypothetical protein [Leptospira interrogans] | 231 | 231 | 95% | 3.00E-73 | 79.90% | WP_001010185.1 |
| hypothetical protein [Leptospira] | 231 | 231 | 95% | 3.00E-73 | 79.90% | WP_001010184.1 |
| hypothetical protein [Leptospira interrogans] | 231 | 231 | 95% | 3.00E-73 | 79.38% | WP_001010182.1 |
| hypothetical protein [Leptospira interrogans] | 231 | 231 | 95% | 3.00E-73 | 79.90% | WP_001010178.1 |
| hypothetical protein [Leptospira interrogans] | 231 | 231 | 95% | 4.00E-73 | 79.90% | WP_001010183.1 |
| hypothetical protein [Leptospira interrogans] | 229 | 229 | 95% | 1.00E-72 | 79.38% | WP_192505591.1 |
| leucine rich repeat protein [Leptospira interrogans serovar Zanoni str. LT2156] | 218 | 218 | 92% | 1.00E-68 | 78.61% | EMM97254.1 |
| hypothetical protein [Leptospira interrogans] | 216 | 216 | 91% | 5.00E-68 | 79.03% | MBW9228790.1 |

```
ALIGNMENTS
>AFV46186.1 leucine-rich repeat protein, partial [Leptospira borgpetersenii
serovar Ballum]
 AFV46187.1 leucine-rich repeat protein, partial [Leptospira borgpetersenii
serovar Mini]
 AFV46188.1 leucine-rich repeat protein, partial [Leptospira borgpetersenii
serovar Sejroe]
Length=203

 Score = 358 bits (920),  Expect = 6e-124, Method: Compositional matrix adjust.
 Identities = 203/203 (100%), Positives = 203/203 (100%), Gaps = 0/203 (0%)

Query  1    GFVCFTASFDCKKNAEEILGEAKAKPELVQTLDFGMQKLSTVPEEVCGFPNLTKLDLRLN  60
            GFVCFTASFDCKKNAEEILGEAKAKPELVQTLDFGMQKLSTVPEEVCGFPNLTKLDLRLN
Sbjct  1    GFVCFTASFDCKKNAEEILGEAKAKPELVQTLDFGMQKLSTVPEEVCGFPNLTKLDLRLN  60

Query  61   SLTFLPEFIGECKRLEQLNLFGNDLTTFPSTFSKLKNLKVLLAGNNDFTILPSELLFLPL  120
            SLTFLPEFIGECKRLEQLNLFGNDLTTFPSTFSKLKNLKVLLAGNNDFTILPSELLFLPL
Sbjct  61   SLTFLPEFIGECKRLEQLNLFGNDLTTFPSTFSKLKNLKVLLAGNNDFTILPSELLFLPL  120

Query  121  IKILYVDRNKLTLTETDVeilaslssleeldlslnsGIKALPFNYEKLVNLINLKRLNIK  180
            IKILYVDRNKLTLTETDVEILASLSSLEELDLSLNSGIKALPFNYEKLVNLINLKRLNIK
Sbjct  121  IKILYVDRNKLTLTETDVEILASLSSLEELDLSLNSGIKALPFNYEKLVNLINLKRLNIK  180

Query  181  KTSLKGEDADKLQAILPNTKIDY  203
            KTSLKGEDADKLQAILPNTKIDY
Sbjct  181  KTSLKGEDADKLQAILPNTKIDY  203
```

```
>ALO26640.1 leucine rich repeat protein [Leptospira borgpetersenii serovar
Ballum]
 ANH01207.1 Leucine rich repeat protein [Leptospira borgpetersenii str. 4E]
 EMN15214.1 leucine rich repeat protein [Leptospira borgpetersenii str. Brem
307]
 EMN18108.1 leucine rich repeat protein [Leptospira borgpetersenii str. Brem
328]
 PTM44326.1 leucine rich repeat (LRR) protein [Leptospira borgpetersenii
serovar Javanica]
 GIM19917.1 hypothetical protein KHM09_23680 [Leptospira borgpetersenii]
Length=222

 Score = 359 bits (921),  Expect = 8e-124, Method: Compositional matrix adjust.
 Identities = 203/203 (100%), Positives = 203/203 (100%), Gaps = 0/203 (0%)

Query  1    GFVCFTASFDCKKNAEEILGEAKAKPELVQTLDFGMQKLSTVPEEVCGFPNLTKLDLRLN  60
            GFVCFTASFDCKKNAEEILGEAKAKPELVQTLDFGMQKLSTVPEEVCGFPNLTKLDLRLN
Sbjct  20   GFVCFTASFDCKKNAEEILGEAKAKPELVQTLDFGMQKLSTVPEEVCGFPNLTKLDLRLN  79

Query  61   SLTFLPEFIGECKRLEQLNLFGNDLTTFPSTFSKLKNLKVLLAGNNDFTILPSELLFLPL  120
            SLTFLPEFIGECKRLEQLNLFGNDLTTFPSTFSKLKNLKVLLAGNNDFTILPSELLFLPL
Sbjct  80   SLTFLPEFIGECKRLEQLNLFGNDLTTFPSTFSKLKNLKVLLAGNNDFTILPSELLFLPL  139

Query  121  IKILYVDRNKLTLTETDVeilaslssleeldlslnsGIKALPFNYEKLVNLINLKRLNIK  180
            IKILYVDRNKLTLTETDVEILASLSSLEELDLSLNSGIKALPFNYEKLVNLINLKRLNIK
Sbjct  140  IKILYVDRNKLTLTETDVEILASLSSLEELDLSLNSGIKALPFNYEKLVNLINLKRLNIK  199

Query  181  KTSLKGEDADKLQAILPNTKIDY  203
            KTSLKGEDADKLQAILPNTKIDY
Sbjct  200  KTSLKGEDADKLQAILPNTKIDY  222


>WP_002731979.1 MULTISPECIES: leucine-rich repeat domain-containing protein
[Leptospira]
 EMG00720.1 leucine rich repeat protein [Leptospira borgpetersenii str. 200701203]
 EMO09583.1 leucine rich repeat protein [Leptospira borgpetersenii str. Noumea
25]
 AXX17321.1 hypothetical protein C4Q31_14790 [Leptospira borgpetersenii serovar
Ceylonica]
 EKP15606.1 leucine rich repeat protein [Leptospira borgpetersenii str. 200801926]
 EKQ91706.1 leucine rich repeat protein [Leptospira borgpetersenii str. UI
09149]
Length=215

 Score = 358 bits (920),  Expect = 1e-123, Method: Compositional matrix adjust.
 Identities = 203/203 (100%), Positives = 203/203 (100%), Gaps = 0/203 (0%)

Query  1    GFVCFTASFDCKKNAEEILGEAKAKPELVQTLDFGMQKLSTVPEEVCGFPNLTKLDLRLN  60
            GFVCFTASFDCKKNAEEILGEAKAKPELVQTLDFGMQKLSTVPEEVCGFPNLTKLDLRLN
Sbjct  13   GFVCFTASFDCKKNAEEILGEAKAKPELVQTLDFGMQKLSTVPEEVCGFPNLTKLDLRLN  72

Query  61   SLTFLPEFIGECKRLEQLNLFGNDLTTFPSTFSKLKNLKVLLAGNNDFTILPSELLFLPL  120
            SLTFLPEFIGECKRLEQLNLFGNDLTTFPSTFSKLKNLKVLLAGNNDFTILPSELLFLPL
Sbjct  73   SLTFLPEFIGECKRLEQLNLFGNDLTTFPSTFSKLKNLKVLLAGNNDFTILPSELLFLPL  132
```

```
Query  121  IKILYVDRNKLTLTETDVeilaslssleeldlslnsGIKALPFNYEKLVNLINLKRLNIK  180
            IKILYVDRNKLTLTETDVEILASLSSLEELDLSLNSGIKALPFNYEKLVNLINLKRLNIK
Sbjct  133  IKILYVDRNKLTLTETDVEILASLSSLEELDLSLNSGIKALPFNYEKLVNLINLKRLNIK  192

Query  181  KTSLKGEDADKLQAILPNTKIDY  203
            KTSLKGEDADKLQAILPNTKIDY
Sbjct  193  KTSLKGEDADKLQAILPNTKIDY  215


>AFV46185.1 leucine-rich repeat protein, partial [Leptospira borgpetersenii
serovar Javanica]
Length=203
 Score = 354 bits (909),  Expect = 3e-122, Method: Compositional matrix adjust.
 Identities = 201/203 (99%), Positives = 202/203 (99%), Gaps = 0/203 (0%)

Query  1    GFVCFTASFDCKKNAEEILGEAKAKPELVQTLDFGMQKLSTVPEEVCGFPNLTKLDLRLN  60
            GF+CFTASFDCKKNAEEILGEAKAKPELVQTLDFGMQKLSTVPEEVCGFPNLTKLDLRLN
Sbjct  1    GFLCFTASFDCKKNAEEILGEAKAKPELVQTLDFGMQKLSTVPEEVCGFPNLTKLDLRLN  60

Query  61   SLTFLPEFIGECKRLEQLNLFGNDLTTFPSTFSKLKNLKVLLAGNNDFTILPSELLFLPL  120
            SLTFLPEFIGECKRLEQLNLFGNDLTTFPSTFSKLKNLKVLLAGNNDFTILPSELLFLPL
Sbjct  61   SLTFLPEFIGECKRLEQLNLFGNDLTTFPSTFSKLKNLKVLLAGNNDFTILPSELLFLPL  120

Query  121  IKILYVDRNKLTLTETDVeilaslssleeldlslnsGIKALPFNYEKLVNLINLKRLNIK  180
            IKILYVDRNKLTLTETDVEILASLSSLEELDLSLNSGIKALPFNYEKLVNLINLKRLNIK
Sbjct  121  IKILYVDRNKLTLTETDVEILASLSSLEELDLSLNSGIKALPFNYEKLVNLINLKRLNIK  180

Query  181  KTSLKGEDADKLQAILPNTKIDY  203
            KTSLKGEDADKLQAIL NTKIDY
Sbjct  181  KTSLKGEDADKLQAILRNTKIDY  203


>EMO62774.1 leucine rich repeat protein [Leptospira borgpetersenii serovar
Pomona str. 200901868]
Length=215

 Score = 354 bits (908),  Expect = 7e-122, Method: Compositional matrix adjust.
 Identities = 201/203 (99%), Positives = 202/203 (99%), Gaps = 0/203 (0%)

Query  1    GFVCFTASFDCKKNAEEILGEAKAKPELVQTLDFGMQKLSTVPEEVCGFPNLTKLDLRLN  60
            GFVCFTASF+CKKNAEEILGEAKAKPELVQTLDFGMQKLSTVPE VCGFPNLTKLDLRLN
Sbjct  13   GFVCFTASFNCKKNAEEILGEAKAKPELVQTLDFGMQKLSTVPEGVCGFPNLTKLDLRLN  72

Query  61   SLTFLPEFIGECKRLEQLNLFGNDLTTFPSTFSKLKNLKVLLAGNNDFTILPSELLFLPL  120
            SLTFLPEFIGECKRLEQLNLFGNDLTTFPSTFSKLKNLKVLLAGNNDFTILPSELLFLPL
Sbjct  73   SLTFLPEFIGECKRLEQLNLFGNDLTTFPSTFSKLKNLKVLLAGNNDFTILPSELLFLPL  132

Query  121  IKILYVDRNKLTLTETDVeilaslssleeldlslnsGIKALPFNYEKLVNLINLKRLNIK  180
            IKILYVDRNKLTLTETDVEILASLSSLEELDLSLNSGIKALPFNYEKLVNLINLKRLNIK
Sbjct  133  IKILYVDRNKLTLTETDVEILASLSSLEELDLSLNSGIKALPFNYEKLVNLINLKRLNIK  192

Query  181  KTSLKGEDADKLQAILPNTKIDY  203
            KTSLKGEDADKLQAILPNTKIDY
Sbjct  193  KTSLKGEDADKLQAILPNTKIDY  215
```

```
>WP_011669694.1 leucine-rich repeat domain-containing protein [Leptospira
borgpetersenii]
 ABJ76745.1 Leucine-rich-repeat protein [Leptospira borgpetersenii serovar
Hardjo-bovis str. JB197]
 ABJ78391.1 Leucine-rich-repeat protein [Leptospira borgpetersenii serovar
Hardjo-bovis str. L550]
 AMX57620.1 hypothetical protein LBK6_04345 [Leptospira borgpetersenii serovar
Hardjo]
 AWV71582.1 hypothetical protein B9T54_04730 [Leptospira borgpetersenii serovar
Hardjo-bovis]
 EMJ80931.1 leucine rich repeat protein [Leptospira borgpetersenii serovar
Hardjo-bovis str. Sponselee]
Length=215

 Score = 352 bits (903),  Expect = 4e-121, Method: Compositional matrix adjust.
 Identities = 200/203 (99%), Positives = 201/203 (99%), Gaps = 0/203 (0%)

Query  1    GFVCFTASFDCKKNAEEILGEAKAKPELVQTLDFGMQKLSTVPEEVCGFPNLTKLDLRLN  60
            GFVCFTASF+CKKNAEEILGEAKAKPELVQTLDFGMQKLSTVPE VCGFPNLTKLDLRLN
Sbjct  13   GFVCFTASFNCKKNAEEILGEAKAKPELVQTLDFGMQKLSTVPEGVCGFPNLTKLDLRLN  72

Query  61   SLTFLPEFIGECKRLEQLNLFGNDLTTFPSTFSKLKNLKVLLAGNNDFTILPSELLFLPL  120
            SLTFLPEFIGECKRLEQLNLFGNDLTTFPSTFSKLKNLKVLLAGNNDFTILPSELLFLPL
Sbjct  73   SLTFLPEFIGECKRLEQLNLFGNDLTTFPSTFSKLKNLKVLLAGNNDFTILPSELLFLPL  132

Query  121  IKILYVDRNKLTLTETDVeilaslssleeldlslnsGIKALPFNYEKLVNLINLKRLNIK  180
            IKILYVDRNKL LTETDVEILASLSSLEELDLSLNSGIKALPFNYEKLVNLINLKRLNIK
Sbjct  133  IKILYVDRNKLILTETDVEILASLSSLEELDLSLNSGIKALPFNYEKLVNLINLKRLNIK  192

Query  181  KTSLKGEDADKLQAILPNTKIDY  203
            KTSLKGEDADKLQAILPNTKIDY
Sbjct  193  KTSLKGEDADKLQAILPNTKIDY  215
>WP_002745429.1 hypothetical protein [Leptospira mayottensis]
 AXR62112.1 hypothetical protein DQM68_17050 [Leptospira mayottensis]
 AXR62988.1 hypothetical protein DQM28_00680 [Leptospira mayottensis]
 AXR66734.1 hypothetical protein DPV73_00510 [Leptospira mayottensis]
 AZQ01438.1 hypothetical protein LEP1GSC190_04660 [Leptospira mayottensis
200901116]
 EKS00741.1 leucine rich repeat protein [Leptospira mayottensis 200901122]
Length=219

 Score = 331 bits (849),  Expect = 8e-113, Method: Compositional matrix adjust.
 Identities = 186/203 (92%), Positives = 191/203 (94%), Gaps = 0/203 (0%)

Query  1    GFVCFTASFDCKKNAEEILGEAKAKPELVQTLDFGMQKLSTVPEEVCGFPNLTKLDLRLN  60
            GF+CFTASFDCKKNAEEILGEAKAKPELVQ LDFGMQKLSTVPE VCGFPNLTKLDLRLN
Sbjct  17   GFICFTASFDCKKNAEEILGEAKAKPELVQILDFGMQKLSTVPEAVCGFPNLTKLDLRLN  76

Query  61   SLTFLPEFIGECKRLEQLNLFGNDLTTFPSTFSKLKNLKVLLAGNNDFTILPSELLFLPL  120
            SLTFLPE IGEC+RLEQLNLFGNDLT  PSTFSKLKNLKVLLAG NDFTILPSELLFLPL
Sbjct  77   SLTFLPESIGECRRLEQLNLFGNDLTALPSTFSKLKNLKVLLAGGNDFTILPSELLFLPL  136

Query  121  IKILYVDRNKLTLTETDVeilaslssleeldlslnsGIKALPFNYEKLVNLINLKRLNIK  180
            I+ LY D+NKLTLTETDVEILASLSSLEELDL+LN GIK LPFNYEKL NL NLKRLNIK
Sbjct  137  IRTLYFDQNKLTLTETDVEILASLSSLEELDLNLNLGIKTLPFNYEKLRNLTNLKRLNIK  196
```

```
Query  181  KTSLKGEDADKLQAILPNTKIDY  203
            KTSLKGEDADKLQAILPNTKIDY
Sbjct  197  KTSLKGEDADKLQAILPNTKIDY  219


>WP_235596156.1 hypothetical protein [Leptospira weilii]
Length=205

 Score = 330 bits (846),  Expect = 1e-112, Method: Compositional matrix adjust.
 Identities = 183/203 (90%), Positives = 194/203 (96%), Gaps = 0/203 (0%)

Query  1    GFVCFTASFDCKKNAEEILGEAKAKPELVQTLDFGMQKLSTVPEEVCGFPNLTKLDLRLN  60
            GF+CF ASFDCKKNAEEILGEAKAKP+ +QTLDFGMQKLST+PE VCGFPNLTKLDLRLN
Sbjct  3    GFICFAASFDCKKNAEEILGEAKAKPKSIQTLDFGMQKLSTIPEGVCGFPNLTKLDLRLN  62

Query  61   SLTFLPEFIGECKRLEQLNLFGNDLTTFPSTFSKLKNLKVLLAGNNDFTILPSELLFLPL  120
            SL  LPEFIGEC+RLEQLNLFGNDL TFPSTFSKLKNLKVLLAG+NDF+ILPSELLFLPL
Sbjct  63   SLISLPEFIGECRRLEQLNLFGNDLATFPSTFSKLKNLKVLLAGSNDFSILPSELLFLPL  122

Query  121  IKILYVDRNKLTLTETDVeilaslssleeldlslnsGIKALPFNYEKLVNLINLKRLNIK  180
            IKILYVD+NKLTLTETDVEILASLS+LEELDL+LN GIK+LPFNYEKL NL NLKRLNIK
Sbjct  123  IKILYVDQNKLTLTETDVEILASLSNLEELDLNLNLGIKSLPFNYEKLGNLTNLKRLNIK  182

Query  181  KTSLKGEDADKLQAILPNTKIDY  203
            KTSLKGEDADKLQAILPNTKIDY
Sbjct  183  KTSLKGEDADKLQAILPNTKIDY  205


>EMJ66028.1 leucine rich repeat protein [Leptospira sp. P2653]
Length=222

 Score = 328 bits (842),  Expect = 1e-111, Method: Compositional matrix adjust.
 Identities = 185/203 (91%), Positives = 193/203 (95%), Gaps = 0/203 (0%)

Query  1    GFVCFTASFDCKKNAEEILGEAKAKPELVQTLDFGMQKLSTVPEEVCGFPNLTKLDLRLN  60
            GF+CFTASFDCKKNAEEILGEAKAK E ++TLDFGMQKLSTVPE VCGFPNLTKLDLRLN
Sbjct  20   GFICFTASFDCKKNAEEILGEAKAKSESIRTLDFGMQKLSTVPEGVCGFPNLTKLDLRLN  79

Query  61   SLTFLPEFIGECKRLEQLNLFGNDLTTFPSTFSKLKNLKVLLAGNNDFTILPSELLFLPL  120
            SLT LPEFIG+CK LEQLNLFGNDL TFP TFSKLKNLKVLLAG+NDFTILPSELLFLPL
Sbjct  80   SLTSLPEFIGDCKNLEQLNLFGNDLATFPPTFSKLKNLKVLLAGSNDFTILPSELLFLPL  139

Query  121  IKILYVDRNKLTLTETDVeilaslssleeldlslnsGIKALPFNYEKLVNLINLKRLNIK  180
            IKILYVDRNKLTLTETDVEILASLS+LEELDL+LN GIK+LPFNYEKL NL NLKRLNIK
Sbjct  140  IKILYVDRNKLTLTETDVEILASLSNLEELDLNLNLGIKSLPFNYEKLGNLTNLKRLNIK  199

Query  181  KTSLKGEDADKLQAILPNTKIDY  203
            KTSLKGEDADKLQAILPNTKIDY
Sbjct  200  KTSLKGEDADKLQAILPNTKIDY  222
```

```
>WP_004496751.1 MULTISPECIES: leucine-rich repeat domain-containing protein
[Leptospira]
 EMM72486.1 leucine rich repeat protein [Leptospira weilii str. 2006001855]
 EKR63142.1 leucine rich repeat protein [Leptospira weilii str. 2006001853]
 EMN43504.1 leucine rich repeat protein [Leptospira weilii str. LNT 1234]
 MCL8266298.1 leucine-rich repeat domain-containing protein [Leptospira weilii]
 OMI17312.1 hypothetical protein BUQ74_11030 [Leptospira weilii serovar Heyan]
Length=215

 Score = 328 bits (840),  Expect = 1e-111, Method: Compositional matrix adjust.
 Identities = 185/203 (91%), Positives = 193/203 (95%), Gaps = 0/203 (0%)

Query  1    GFVCFTASFDCKKNAEEILGEAKAKPELVQTLDFGMQKLSTVPEEVCGFPNLTKLDLRLN  60
            GF+CFTASFDCKKNAEEILGEAKAK E ++TLDFGMQKLSTVPE VCGFPNLTKLDLRLN
Sbjct  13   GFICFTASFDCKKNAEEILGEAKAKSESIRTLDFGMQKLSTVPEGVCGFPNLTKLDLRLN  72

Query  61   SLTFLPEFIGECKRLEQLNLFGNDLTTFPSTFSKLKNLKVLLAGNNDFTILPSELLFLPL  120
            SLT LPEFIG+CK LEQLNLFGNDL TFP TFSKLKNLKVLLAG+NDFTILPSELLFLPL
Sbjct  73   SLTSLPEFIGDCKNLEQLNLFGNDLATFPPTFSKLKNLKVLLAGSNDFTILPSELLFLPL  132

Query  121  IKILYVDRNKLTLTETDVeilaslssleeldlslnsGIKALPFNYEKLVNLINLKRLNIK  180
            IKILYVDRNKLTLTETDVEILASLS+LEELDL+LN GIK+LPFNYEKL NL NLKRLNIK
Sbjct  133  IKILYVDRNKLTLTETDVEILASLSNLEELDLNLNLGIKSLPFNYEKLGNLTNLKRLNIK  192

Query  181  KTSLKGEDADKLQAILPNTKIDY  203
            KTSLKGEDADKLQAILPNTKIDY
Sbjct  193  KTSLKGEDADKLQAILPNTKIDY  215


>WP_061219130.1 leucine-rich repeat domain-containing protein [Leptospira weilii]
Length=215

 Score = 326 bits (835),  Expect = 8e-111, Method: Compositional matrix adjust.
 Identities = 184/203 (91%), Positives = 193/203 (95%), Gaps = 0/203 (0%)

Query  1    GFVCFTASFDCKKNAEEILGEAKAKPELVQTLDFGMQKLSTVPEEVCGFPNLTKLDLRLN  60
            GF+CFTASFDCKKNAEEILGEAKAK E ++TLDFGMQKLSTVPE VCGFPNLTKLDLRLN
Sbjct  13   GFICFTASFDCKKNAEEILGEAKAKSESIRTLDFGMQKLSTVPEGVCGFPNLTKLDLRLN  72

Query  61   SLTFLPEFIGECKRLEQLNLFGNDLTTFPSTFSKLKNLKVLLAGNNDFTILPSELLFLPL  120
            SLT LPEFIG+CK LEQLNLFGNDL TFP TFSKLKNLKVLLAG+NDFTILPSELLFLPL
Sbjct  73   SLTSLPEFIGDCKNLEQLNLFGNDLATFPPTFSKLKNLKVLLAGSNDFTILPSELLFLPL  132

Query  121  IKILYVDRNKLTLTETDVeilaslssleeldlslnsGIKALPFNYEKLVNLINLKRLNIK  180
            IKILYVD+NKLTLTETDVEILASLS+LEELDL+LN GIK+LPFNYEKL NL NLKRLNIK
Sbjct  133  IKILYVDQNKLTLTETDVEILASLSNLEELDLNLNLGIKSLPFNYEKLGNLTNLKRLNIK  192

Query  181  KTSLKGEDADKLQAILPNTKIDY  203
            KTSLKGEDADKLQAILPNTKIDY
Sbjct  193  KTSLKGEDADKLQAILPNTKIDY  215
```

```
>EMN91031.1 leucine rich repeat protein [Leptospira weilii str. UI 13098]
Length=222

 Score = 325 bits (834),  Expect = 1e-110, Method: Compositional matrix adjust.
 Identities = 184/203 (91%), Positives = 192/203 (95%), Gaps = 0/203 (0%)

Query  1    GFVCFTASFDCKKNAEEILGEAKAKPELVQTLDFGMQKLSTVPEEVCGFPNLTKLDLRLN  60
            GF+CFTASFDCKKNAEEILGEAKAK E ++TLDFGMQKLSTVPE VCGFPNLTKLDLRLN
Sbjct  20   GFICFTASFDCKKNAEEILGEAKAKSESIRTLDFGMQKLSTVPEGVCGFPNLTKLDLRLN  79

Query  61   SLTFLPEFIGECKRLEQLNLFGNDLTTFPSTFSKLKNLKVLLAGNNDFTILPSELLFLPL  120
            SLT LPEFIG+CK LEQLNLFGNDL TFP TFSKLKNLKVLLAG+NDFTILPSELLFLPL
Sbjct  80   SLTSLPEFIGDCKNLEQLNLFGNDLATFPPTFSKLKNLKVLLAGSNDFTILPSELLFLPL  139

Query  121  IKILYVDRNKLTLTETDVeilaslssleeldlslnsGIKALPFNYEKLVNLINLKRLNIK  180
            IKILYVDRNKLTLTETDVEILASLS+LEELDL+LN  IK+LPFNYEKL NL NLKRLNIK
Sbjct  140  IKILYVDRNKLTLTETDVEILASLSNLEELDLNLNLEIKSLPFNYEKLGNLTNLKRLNIK  199

Query  181  KTSLKGEDADKLQAILPNTKIDY  203
            KTSLKGEDADKLQAILPNTKIDY
Sbjct  200  KTSLKGEDADKLQAILPNTKIDY  222


>WP_026054684.1 leucine-rich repeat domain-containing protein [Leptospira weilii]
Length=215

 Score = 325 bits (832),  Expect = 2e-110, Method: Compositional matrix adjust.
 Identities = 184/203 (91%), Positives = 192/203 (95%), Gaps = 0/203 (0%)

Query  1    GFVCFTASFDCKKNAEEILGEAKAKPELVQTLDFGMQKLSTVPEEVCGFPNLTKLDLRLN  60
            GF+CFTASFDCKKNAEEILGEAKAK E ++TLDFGMQKLSTVPE VCGFPNLTKLDLRLN
Sbjct  13   GFICFTASFDCKKNAEEILGEAKAKSESIRTLDFGMQKLSTVPEGVCGFPNLTKLDLRLN  72

Query  61   SLTFLPEFIGECKRLEQLNLFGNDLTTFPSTFSKLKNLKVLLAGNNDFTILPSELLFLPL  120
            SLT LPEFIG+CK LEQLNLFGNDL TFP TFSKLKNLKVLLAG+NDFTILPSELLFLPL
Sbjct  73   SLTSLPEFIGDCKNLEQLNLFGNDLATFPPTFSKLKNLKVLLAGSNDFTILPSELLFLPL  132

Query  121  IKILYVDRNKLTLTETDVeilaslssleeldlslnsGIKALPFNYEKLVNLINLKRLNIK  180
            IKILYVDRNKLTLTETDVEILASLS+LEELDL+LN  IK+LPFNYEKL NL NLKRLNIK
Sbjct  133  IKILYVDRNKLTLTETDVEILASLSNLEELDLNLNLEIKSLPFNYEKLGNLTNLKRLNIK  192

Query  181  KTSLKGEDADKLQAILPNTKIDY  203
            KTSLKGEDADKLQAILPNTKIDY
Sbjct  193  KTSLKGEDADKLQAILPNTKIDY  215
```

```
>WP_061217745.1 leucine-rich repeat domain-containing protein [Leptospira weilii]
Length=215

 Score = 322 bits (826),  Expect = 2e-109, Method: Compositional matrix adjust.
 Identities = 183/202 (91%), Positives = 191/202 (95%), Gaps = 0/202 (0%)

Query  2    FVCFTASFDCKKNAEEILGEAKAKPELVQTLDFGMQKLSTVPEEVCGFPNLTKLDLRLNS  61
            F+CFTASFDCKKNAEEILGEAKAK E ++TLDFGMQKLSTVPE VCGFPNLTKLDLRLNS
Sbjct  14   FICFTASFDCKKNAEEILGEAKAKSESIRTLDFGMQKLSTVPEGVCGFPNLTKLDLRLNS  73

Query  62   LTFLPEFIGECKRLEQLNLFGNDLTTFPSTFSKLKNLKVLLAGNNDFTILPSELLFLPLI  121
            LT LPEFIG+CK LEQLNLFGNDL TFP TFSKLKNLKVLLAG+NDFTILPSELLFLPLI
Sbjct  74   LTSLPEFIGDCKNLEQLNLFGNDLATFPPTFSKLKNLKVLLAGSNDFTILPSELLFLPLI  133

Query  122  KILYVDRNKLTLTETDVeilaslssleeldlslnsGIKALPFNYEKLVNLINLKRLNIKK  181
            KILYVDRNKLTLTETDVEILASLS+LEELDL+LN  IK+LPFNYEKL NL NLKRLNIKK
Sbjct  134  KILYVDRNKLTLTETDVEILASLSNLEELDLNLNLEIKSLPFNYEKLGNLTNLKRLNIKK  193

Query  182  TSLKGEDADKLQAILPNTKIDY  203
            TSLKGEDADKLQAILPNTKIDY
Sbjct  194  TSLKGEDADKLQAILPNTKIDY  215


>EQA64549.1 leucine rich repeat protein [Leptospira alexanderi serovar Manhao
3 str. L 60]
Length=222

 Score = 312 bits (800),  Expect = 2e-105, Method: Compositional matrix adjust.
 Identities = 176/203 (87%), Positives = 187/203 (92%), Gaps = 0/203 (0%)

Query  1    GFVCFTASFDCKKNAEEILGEAKAKPELVQTLDFGMQKLSTVPEEVCGFPNLTKLDLRLN  60
            GF+CFTASFDCKKNAEEILGEAKAKPELVQTLDFGMQKLSTVPE VCGFPNLTKLDLRLN
Sbjct  20   GFICFTASFDCKKNAEEILGEAKAKPELVQTLDFGMQKLSTVPEGVCGFPNLTKLDLRLN  79

Query  61   SLTFLPEFIGECKRLEQLNLFGNDLTTFPSTFSKLKNLKVLLAGNNDFTILPSELLFLPL  120
            SL+ LP+ IG+CK LEQLNLFGNDLTTFP+  SKLK L++LLAGNND   LPSELLFLP
Sbjct  80   SLSSLPDEIGDCKNLEQLNLFGNDLTTFPAALSKLKKLRILLAGNNDLKFLPSELLFLPE  139

Query  121  IKILYVDRNKLTLTETDVeilaslssleeldlslnsGIKALPFNYEKLVNLINLKRLNIK  180
            IK +Y+D+NKLTLTETDVEILASLSSLEELDL+LN GIK LPFNYEKL NL NLKRLNIK
Sbjct  140  IKTIYMDQNKLTLTETDVEILASLSSLEELDLNLNLGIKTLPFNYEKLGNLTNLKRLNIK  199

Query  181  KTSLKGEDADKLQAILPNTKIDY  203
            KTSLKGEDADKLQAILPNTKIDY
Sbjct  200  KTSLKGEDADKLQAILPNTKIDY  222
```

```
>WP_039940928.1 hypothetical protein [Leptospira alexanderi]
Length=215

 Score = 311 bits (798),  Expect = 3e-105, Method: Compositional matrix adjust.
 Identities = 176/203 (87%), Positives = 187/203 (92%), Gaps = 0/203 (0%)

Query  1    GFVCFTASFDCKKNAEEILGEAKAKPELVQTLDFGMQKLSTVPEEVCGFPNLTKLDLRLN  60
            GF+CFTASFDCKKNAEEILGEAKAKPELVQTLDFGMQKLSTVPE VCGFPNLTKLDLRLN
Sbjct  13   GFICFTASFDCKKNAEEILGEAKAKPELVQTLDFGMQKLSTVPEGVCGFPNLTKLDLRLN  72

Query  61   SLTFLPEFIGECKRLEQLNLFGNDLTTFPSTFSKLKNLKVLLAGNNDFTILPSELLFLPL  120
            SL+ LP+ IG+CK LEQLNLFGNDLTTFP+  SKLK L++LLAGNND   LPSELLFLP
Sbjct  73   SLSSLPDEIGDCKNLEQLNLFGNDLTTFPAALSKLKKLRILLAGNNDLKFLPSELLFLPE  132

Query  121  IKILYVDRNKLTLTETDVeilaslssleeldlslnsGIKALPFNYEKLVNLINLKRLNIK  180
            IK +Y+D+NKLTLTETDVEILASLSSLEELDL+LN GIK LPFNYEKL NL NLKRLNIK
Sbjct  133  IKTIYMDQNKLTLTETDVEILASLSSLEELDLNLNLGIKTLPFNYEKLGNLTNLKRLNIK  192

Query  181  KTSLKGEDADKLQAILPNTKIDY  203
            KTSLKGEDADKLQAILPNTKIDY
Sbjct  193  KTSLKGEDADKLQAILPNTKIDY  215


>WP_078124592.1 hypothetical protein [Leptospira alexanderi]
Length=215

 Score = 309 bits (791),  Expect = 4e-104, Method: Compositional matrix adjust.
 Identities = 175/203 (86%), Positives = 186/203 (92%), Gaps = 0/203 (0%)

Query  1    GFVCFTASFDCKKNAEEILGEAKAKPELVQTLDFGMQKLSTVPEEVCGFPNLTKLDLRLN  60
            GF+CFTASFDCKKNAEEILGEAKAKPELVQTLDFGMQKLSTVPE VCGFPNLTKLDLRLN
Sbjct  13   GFICFTASFDCKKNAEEILGEAKAKPELVQTLDFGMQKLSTVPEGVCGFPNLTKLDLRLN  72

Query  61   SLTFLPEFIGECKRLEQLNLFGNDLTTFPSTFSKLKNLKVLLAGNNDFTILPSELLFLPL  120
            SL+ LP+ IG+CK LEQLNLFGNDLTTFP+  SKLK L++LLAGNND   LPSELLFLP
Sbjct  73   SLSSLPDEIGDCKNLEQLNLFGNDLTTFPAALSKLKKLRILLAGNNDLKFLPSELLFLPE  132

Query  121  IKILYVDRNKLTLTETDVeilaslssleeldlslnsGIKALPFNYEKLVNLINLKRLNIK  180
            IK +Y+D+NKLTLTETDVEILASLSSLEELDL+LN GIK LPFNYEKL NL NLKRLNIK
Sbjct  133  IKTIYMDQNKLTLTETDVEILASLSSLEELDLNLNLGIKTLPFNYEKLGNLTNLKRLNIK  192

Query  181  KTSLKGEDADKLQAILPNTKIDY  203
            KTSLKG DADKLQAILPNTKIDY
Sbjct  193  KTSLKGGDADKLQAILPNTKIDY  215
```

```
>WP_004466481.1 leucine-rich repeat domain-containing protein [Leptospira santarosai]
 EMM76134.1 leucine rich repeat protein [Leptospira santarosai str. 2000030832]
Length=217

 Score = 307 bits (787),  Expect = 2e-103, Method: Compositional matrix adjust.
 Identities = 177/205 (86%), Positives = 184/205 (90%), Gaps = 2/205 (1%)

Query  1    GFVCFTASF--DCKKNAEEILGEAKAKPELVQTLDFGMQKLSTVPEEVCGFPNLTKLDLR  58
            GF+CFTAS   DCKKN EEIL EAKA+PE VQTLDFGM+KLS VPE VCGFPNLTKLDLR
Sbjct  13   GFICFTASLEIDCKKNTEEILEEAKARPESVQTLDFGMRKLSAVPEGVCGFPNLTKLDLR  72

Query  59   LNSLTFLPEFIGECKRLEQLNLFGNDLTTFPSTFSKLKNLKVLLAGNNDFTILPSELLFL  118
            LNSLT LPEFIGECK LEQLNLFGNDLTT PSTFSKLKNLKVLLAG+NDFT+LPSELLFL
Sbjct  73   LNSLTSLPEFIGECKSLEQLNLFGNDLTTLPSTFSKLKNLKVLLAGSNDFTVLPSELLFL  132

Query  119  PLIKILYVDRNKLTLTETDVeilaslssleeldlslnsGIKALPFNYEKLVNLINLKRLN  178
            P IK LYVDRN+LTLTETDVEILASLSSLEELDL+ NSGI  LPFNYEKL NL  LKRLN
Sbjct  133  PSIKTLYVDRNRLTLTETDVEILASLSSLEELDLNQNSGIGTLPFNYEKLRNLTGLKRLN  192

Query  179  IKKTSLKGEDADKLQAILPNTKIDY  203
            IKKTSLKGEDA KLQAILPN KIDY
Sbjct  193  IKKTSLKGEDAGKLQAILPNAKIDY  217


>WP_046692451.1 leucine-rich repeat domain-containing protein [Leptospira santarosai]
 AVQ11281.1 Leucine rich repeat protein [Leptospira santarosai]
Length=217

 Score = 306 bits (785),  Expect = 4e-103, Method: Compositional matrix adjust.
 Identities = 176/205 (86%), Positives = 184/205 (90%), Gaps = 2/205 (1%)

Query  1    GFVCFTASF--DCKKNAEEILGEAKAKPELVQTLDFGMQKLSTVPEEVCGFPNLTKLDLR  58
            GF+CFTAS   DCKKN EEIL EAKA+PE VQTLDFGM+KLS VPE VCGFPNL KLDLR
Sbjct  13   GFICFTASLEIDCKKNTEEILEEAKARPESVQTLDFGMRKLSAVPEGVCGFPNLIKLDLR  72

Query  59   LNSLTFLPEFIGECKRLEQLNLFGNDLTTFPSTFSKLKNLKVLLAGNNDFTILPSELLFL  118
            LNSLT LPEFIGECKRLEQLNLFGNDLTT PSTFSKLKNLKVLL G+NDFT+LPSELLFL
Sbjct  73   LNSLTSLPEFIGECKRLEQLNLFGNDLTTLPSTFSKLKNLKVLLVGSNDFTVLPSELLFL  132

Query  119  PLIKILYVDRNKLTLTETDVeilaslssleeldlslnsGIKALPFNYEKLVNLINLKRLN  178
            P IK LYVDRN+LTLTETDVEILASLSSLEELDL+ NSGI+ LPFNYEKL NL  LKRLN
Sbjct  133  PSIKTLYVDRNRLTLTETDVEILASLSSLEELDLNQNSGIETLPFNYEKLRNLTGLKRLN  192

Query  179  IKKTSLKGEDADKLQAILPNTKIDY  203
            IKKTSLKGEDA KLQAILPN KIDY
Sbjct  193  IKKTSLKGEDAGKLQAILPNAKIDY  217
```

```
>EKT85788.2 hypothetical protein LSS_15741 [Leptospira santarosai serovar
Shermani str. LT 821]
Length=224

 Score = 305 bits (782),  Expect = 1e-102, Method: Compositional matrix adjust.
 Identities = 176/205 (86%), Positives = 183/205 (89%), Gaps = 2/205 (1%)

Query  1    GFVCFTASF--DCKKNAEEILGEAKAKPELVQTLDFGMQKLSTVPEEVCGFPNLTKLDLR  58
            GF+CFTAS   DCKKN EEIL EAKA+PE VQTLDFGM+KLS VPE VCGFPNL KLDLR
Sbjct  20   GFICFTASLEIDCKKNTEEILEEAKARPESVQTLDFGMRKLSAVPEGVCGFPNLIKLDLR  79

Query  59   LNSLTFLPEFIGECKRLEQLNLFGNDLTTFPSTFSKLKNLKVLLAGNNDFTILPSELLFL  118
            LNSLT LPEFIGECK LEQLNLFGNDLTT PSTFSKLKNLKVLLAG+NDFT+LPSELLFL
Sbjct  80   LNSLTSLPEFIGECKSLEQLNLFGNDLTTLPSTFSKLKNLKVLLAGSNDFTVLPSELLFL  139

Query  119  PLIKILYVDRNKLTLTETDVeilaslssleeldlslnsGIKALPFNYEKLVNLINLKRLN  178
            P IK LYVDRN+LTLTETDVEILASLSSLEELDL+ NSGI  LPFNYEKL NL  LKRLN
Sbjct  140  PSIKTLYVDRNRLTLTETDVEILASLSSLEELDLNQNSGIGTLPFNYEKLRNLTGLKRLN  199

Query  179  IKKTSLKGEDADKLQAILPNTKIDY  203
            IKKTSLKGEDA KLQAILPN KIDY
Sbjct  200  IKKTSLKGEDAGKLQAILPNAKIDY  224
```

```
>WP_004471631.1 MULTISPECIES: leucine-rich repeat domain-containing protein
[Leptospira]
 ASV13101.1 hypothetical protein B2G51_00410 [Leptospira santarosai]
 EKO79664.1 leucine rich repeat protein [Leptospira sp. Fiocruz LV3954]
 EKS08892.1 leucine rich repeat protein [Leptospira santarosai str. JET]
 EMI62768.1 leucine rich repeat protein [Leptospira sp. Fiocruz LV4135]
 EMN21750.1 leucine rich repeat protein [Leptospira santarosai serovar Arenal
str. MAVJ 401]
Length=217

 Score = 305 bits (781),  Expect = 2e-102, Method: Compositional matrix adjust.
 Identities = 176/205 (86%), Positives = 183/205 (89%), Gaps = 2/205 (1%)

Query  1    GFVCFTASF--DCKKNAEEILGEAKAKPELVQTLDFGMQKLSTVPEEVCGFPNLTKLDLR  58
            GF+CFTAS   DCKKN EEIL EAKA+PE VQTLDFGM+KLS VPE VCGFPNL KLDLR
Sbjct  13   GFICFTASLEIDCKKNTEEILEEAKARPESVQTLDFGMRKLSAVPEGVCGFPNLIKLDLR  72

Query  59   LNSLTFLPEFIGECKRLEQLNLFGNDLTTFPSTFSKLKNLKVLLAGNNDFTILPSELLFL  118
            LNSLT LPEFIGECK LEQLNLFGNDLTT PSTFSKLKNLKVLLAG+NDFT+LPSELLFL
Sbjct  73   LNSLTSLPEFIGECKSLEQLNLFGNDLTTLPSTFSKLKNLKVLLAGSNDFTVLPSELLFL  132

Query  119  PLIKILYVDRNKLTLTETDVeilaslssleeldlslnsGIKALPFNYEKLVNLINLKRLN  178
            P IK LYVDRN+LTLTETDVEILASLSSLEELDL+ NSGI  LPFNYEKL NL  LKRLN
Sbjct  133  PSIKTLYVDRNRLTLTETDVEILASLSSLEELDLNQNSGIGTLPFNYEKLRNLTGLKRLN  192

Query  179  IKKTSLKGEDADKLQAILPNTKIDY  203
            IKKTSLKGEDA KLQAILPN KIDY
Sbjct  193  IKKTSLKGEDAGKLQAILPNAKIDY  217


>WP_061216203.1 leucine-rich repeat domain-containing protein [Leptospira santarosai]
Length=217

 Score = 305 bits (781),  Expect = 2e-102, Method: Compositional matrix adjust.
 Identities = 176/205 (86%), Positives = 183/205 (89%), Gaps = 2/205 (1%)

Query  1    GFVCFTASF--DCKKNAEEILGEAKAKPELVQTLDFGMQKLSTVPEEVCGFPNLTKLDLR  58
            GF+CFTAS   DCKKN EEIL EAKA+PE VQTLDFGM+KLS VPE VCGFPNL KLDLR
Sbjct  13   GFICFTASLEIDCKKNTEEILEEAKARPESVQTLDFGMRKLSAVPEGVCGFPNLIKLDLR  72

Query  59   LNSLTFLPEFIGECKRLEQLNLFGNDLTTFPSTFSKLKNLKVLLAGNNDFTILPSELLFL  118
            LNSLT LPEFIGECK LEQLNLFGNDLTT PSTFSKLKNLKVLLAG+NDFT+LPSELLFL
Sbjct  73   LNSLTSLPEFIGECKSLEQLNLFGNDLTTLPSTFSKLKNLKVLLAGSNDFTVLPSELLFL  132

Query  119  PLIKILYVDRNKLTLTETDVeilaslssleeldlslnsGIKALPFNYEKLVNLINLKRLN  178
            P IK LYVDRN+LTLTETDVEILASLSSLEELDL+ NSGI  LPFNYEKL NL  LKRLN
Sbjct  133  PSIKTLYVDRNRLTLTETDVEILASLSSLEELDLNQNSGIGTLPFNYEKLRNLTGLKRLN  192

Query  179  IKKTSLKGEDADKLQAILPNTKIDY  203
            IKKTSLKGEDA KLQAILPN KIDY
Sbjct  193  IKKTSLKGEDAGKLQAILPNVKIDY  217
```

```
>WP_004492778.1 leucine-rich repeat domain-containing protein [Leptospira santarosai]
 EMF91397.1 leucine rich repeat protein [Leptospira santarosai str. ST188]
Length=217

 Score = 304 bits (779),  Expect = 3e-102, Method: Compositional matrix adjust.
 Identities = 176/205 (86%), Positives = 183/205 (89%), Gaps = 2/205 (1%)

Query  1    GFVCFTASF--DCKKNAEEILGEAKAKPELVQTLDFGMQKLSTVPEEVCGFPNLTKLDLR  58
            GF+CFTAS   DCKKN EEIL EAKA+PE VQTLDFGM+KLS VPE VCGFPNL KLDLR
Sbjct  13   GFICFTASLEIDCKKNTEEILEEAKARPESVQTLDFGMRKLSEVPEGVCGFPNLIKLDLR  72

Query  59   LNSLTFLPEFIGECKRLEQLNLFGNDLTTFPSTFSKLKNLKVLLAGNNDFTILPSELLFL  118
            LNSLT LPEFIGECK LEQLNLFGNDLTT PSTFSKLKNLKVLLAG+NDFT+LPSELLFL
Sbjct  73   LNSLTSLPEFIGECKSLEQLNLFGNDLTTLPSTFSKLKNLKVLLAGSNDFTVLPSELLFL  132

Query  119  PLIKILYVDRNKLTLTETDVeilaslssleeldlslnsGIKALPFNYEKLVNLINLKRLN  178
            P IK LYVDRN+LTLTETDVEILASLSSLEELDL+ NSGI  LPFNYEKL NL  LKRLN
Sbjct  133  PSIKTLYVDRNRLTLTETDVEILASLSSLEELDLNQNSGIGTLPFNYEKLRNLTGLKRLN  192

Query  179  IKKTSLKGEDADKLQAILPNTKIDY  203
            IKKTSLKGEDA KLQAILPN KIDY
Sbjct  193  IKKTSLKGEDAGKLQAILPNAKIDY  217


>EMO58507.1 leucine rich repeat protein [Leptospira santarosai str. CBC1416]
Length=224

 Score = 305 bits (780),  Expect = 3e-102, Method: Compositional matrix adjust.
 Identities = 175/205 (85%), Positives = 183/205 (89%), Gaps = 2/205 (1%)

Query  1    GFVCFTASF--DCKKNAEEILGEAKAKPELVQTLDFGMQKLSTVPEEVCGFPNLTKLDLR  58
            GF+CFTAS   DCKKN EEIL EAKA+PE VQTLDFGM+KLS VPE VCGFPNL KLDLR
Sbjct  20   GFICFTASLEIDCKKNTEEILEEAKARPESVQTLDFGMRKLSAVPEGVCGFPNLIKLDLR  79

Query  59   LNSLTFLPEFIGECKRLEQLNLFGNDLTTFPSTFSKLKNLKVLLAGNNDFTILPSELLFL  118
            LNSLT LPEFIGECK LEQLNLFGNDLTT PSTFSKLKNL+VLLAG+NDFT+LPSELLFL
Sbjct  80   LNSLTSLPEFIGECKSLEQLNLFGNDLTTLPSTFSKLKNLQVLLAGSNDFTVLPSELLFL  139

Query  119  PLIKILYVDRNKLTLTETDVeilaslssleeldlslnsGIKALPFNYEKLVNLINLKRLN  178
            P IK LYVDRN+LTLTETDVEILASLSSLEELDL+ NSGI  LPFNYEKL NL  LKRLN
Sbjct  140  PSIKTLYVDRNRLTLTETDVEILASLSSLEELDLNQNSGIGTLPFNYEKLRNLTGLKRLN  199

Query  179  IKKTSLKGEDADKLQAILPNTKIDY  203
            IKKTSLKGEDA KLQAILPN KIDY
Sbjct  200  IKKTSLKGEDAGKLQAILPNAKIDY  224
```

```
>WP_004468285.1 leucine-rich repeat domain-containing protein [Leptospira santarosai]
 EKO33314.1 leucine rich repeat protein [Leptospira santarosai str. MOR084]
 EMM87883.1 leucine rich repeat protein [Leptospira santarosai str. 2000027870]
 EMO12096.1 leucine rich repeat protein [Leptospira santarosai str. CBC523]
 EMO21776.1 leucine rich repeat protein [Leptospira santarosai str. HAI134]
Length=217

 Score = 304 bits (779),  Expect = 4e-102, Method: Compositional matrix adjust.
 Identities = 175/205 (85%), Positives = 183/205 (89%), Gaps = 2/205 (1%)

Query  1    GFVCFTASF--DCKKNAEEILGEAKAKPELVQTLDFGMQKLSTVPEEVCGFPNLTKLDLR  58
            GF+CFTAS   DCKKN EEIL EAKA+PE VQTLDFGM+KLS VPE VCGFPNL KLDLR
Sbjct  13   GFICFTASLEIDCKKNTEEILEEAKARPESVQTLDFGMRKLSAVPEGVCGFPNLIKLDLR  72

Query  59   LNSLTFLPEFIGECKRLEQLNLFGNDLTTFPSTFSKLKNLKVLLAGNNDFTILPSELLFL  118
            LNSLT LPEFIGECK LEQLNLFGNDLTT PSTFSKLKNL+VLLAG+NDFT+LPSELLFL
Sbjct  73   LNSLTSLPEFIGECKSLEQLNLFGNDLTTLPSTFSKLKNLQVLLAGSNDFTVLPSELLFL  132

Query  119  PLIKILYVDRNKLTLTETDVeilaslssleeldlslnsGIKALPFNYEKLVNLINLKRLN  178
            P IK LYVDRN+LTLTETDVEILASLSSLEELDL+ NSGI  LPFNYEKL NL  LKRLN
Sbjct  133  PSIKTLYVDRNRLTLTETDVEILASLSSLEELDLNQNSGIGTLPFNYEKLRNLTGLKRLN  192

Query  179  IKKTSLKGEDADKLQAILPNTKIDY  203
            IKKTSLKGEDA KLQAILPN KIDY
Sbjct  193  IKKTSLKGEDAGKLQAILPNAKIDY  217


>WP_004486481.1 leucine-rich repeat domain-containing protein [Leptospira santarosai]
 EMJ51727.1 leucine rich repeat protein [Leptospira santarosai str. HAI1349]
 EMO43962.1 leucine rich repeat protein [Leptospira santarosai str. ZUN179]
 EMP80142.1 leucine rich repeat protein [Leptospira santarosai str. CBC1531]
Length=217

 Score = 303 bits (777),  Expect = 6e-102, Method: Compositional matrix adjust.
 Identities = 174/205 (85%), Positives = 183/205 (89%), Gaps = 2/205 (1%)

Query  1    GFVCFTASF--DCKKNAEEILGEAKAKPELVQTLDFGMQKLSTVPEEVCGFPNLTKLDLR  58
            GF+CFTAS   DCKKN EEIL EAKA+PE +QTLDFGM+KLS VPE VCGFPNL KLDLR
Sbjct  13   GFICFTASLEIDCKKNTEEILEEAKARPESIQTLDFGMRKLSAVPEGVCGFPNLIKLDLR  72

Query  59   LNSLTFLPEFIGECKRLEQLNLFGNDLTTFPSTFSKLKNLKVLLAGNNDFTILPSELLFL  118
            LNSLT LPEFIGECK LEQLNLFGNDLTT PSTFSKLKNL+VLLAG+NDFT+LPSELLFL
Sbjct  73   LNSLTSLPEFIGECKSLEQLNLFGNDLTTLPSTFSKLKNLQVLLAGSNDFTVLPSELLFL  132

Query  119  PLIKILYVDRNKLTLTETDVeilaslssleeldlslnsGIKALPFNYEKLVNLINLKRLN  178
            P IK LYVDRN+LTLTETDVEILASLSSLEELDL+ NSGI  LPFNYEKL NL  LKRLN
Sbjct  133  PSIKTLYVDRNRLTLTETDVEILASLSSLEELDLNQNSGIGTLPFNYEKLRNLTGLKRLN  192

Query  179  IKKTSLKGEDADKLQAILPNTKIDY  203
            IKKTSLKGEDA KLQAILPN KIDY
Sbjct  193  IKKTSLKGEDAGKLQAILPNAKIDY  217
```

```
>WP_004473752.1 leucine-rich repeat domain-containing protein [Leptospira santarosai]
 AVV50234.1 Leucine rich repeat protein [Leptospira santarosai]
 AVV80614.1 Leucine rich repeat protein [Leptospira santarosai]
 EMO70761.1 leucine rich repeat protein [Leptospira santarosai str. 200403458]
 EMO99203.1 leucine rich repeat protein [Leptospira santarosai str. 200702252]
 KXZ30505.1 hypothetical protein AYB33_17160 [Leptospira santarosai]
Length=217

 Score = 303 bits (776),  Expect = 8e-102, Method: Compositional matrix adjust.
 Identities = 175/205 (85%), Positives = 182/205 (89%), Gaps = 2/205 (1%)

Query  1    GFVCFTASF--DCKKNAEEILGEAKAKPELVQTLDFGMQKLSTVPEEVCGFPNLTKLDLR  58
            GF+CFTA    DCKKN EEIL EAKA+PE VQTLDFGM+KLS VPE VCGFPNL KLDLR
Sbjct  13   GFICFTAGLEIDCKKNTEEILEEAKARPESVQTLDFGMRKLSAVPEGVCGFPNLIKLDLR  72

Query  59   LNSLTFLPEFIGECKRLEQLNLFGNDLTTFPSTFSKLKNLKVLLAGNNDFTILPSELLFL  118
            LNSLT LPEFIGECK LEQLNLFGNDLTT PSTFSKLKNLKVLLAG+NDFT+LPSELLFL
Sbjct  73   LNSLTSLPEFIGECKSLEQLNLFGNDLTTLPSTFSKLKNLKVLLAGSNDFTVLPSELLFL  132

Query  119  PLIKILYVDRNKLTLTETDVeilaslssleeldlslnsGIKALPFNYEKLVNLINLKRLN  178
            P IK LYVDRN+LTLTETDVEILASLSSLEELDL+ NSGI  LPFNYEKL NL  LKRLN
Sbjct  133  PSIKTLYVDRNRLTLTETDVEILASLSSLEELDLNQNSGIGTLPFNYEKLRNLTGLKRLN  192

Query  179  IKKTSLKGEDADKLQAILPNTKIDY  203
            IKKTSLKGEDA KLQAILPN KIDY
Sbjct  193  IKKTSLKGEDAGKLQAILPNAKIDY  217


>WP_004476114.1 leucine-rich repeat domain-containing protein [Leptospira santarosai]
 EKR92365.1 leucine rich repeat protein [Leptospira santarosai str. CBC379]
 EMO32349.1 leucine rich repeat protein [Leptospira santarosai str. HAI821]
 EMP02889.1 leucine rich repeat protein [Leptospira santarosai str. HAI1380]
Length=217

 Score = 302 bits (774),  Expect = 2e-101, Method: Compositional matrix adjust.
 Identities = 174/205 (85%), Positives = 182/205 (89%), Gaps = 2/205 (1%)

Query  1    GFVCFTASF--DCKKNAEEILGEAKAKPELVQTLDFGMQKLSTVPEEVCGFPNLTKLDLR  58
            GF+CFTA    DCKKN EEIL EAKA+PE VQTLDFGM+KLS VPE VCGFPNL KLDLR
Sbjct  13   GFICFTAGLEIDCKKNTEEILEEAKARPESVQTLDFGMRKLSAVPEGVCGFPNLIKLDLR  72

Query  59   LNSLTFLPEFIGECKRLEQLNLFGNDLTTFPSTFSKLKNLKVLLAGNNDFTILPSELLFL  118
            LNSLT LPEFIGECK LEQLNLFGNDLTT PSTFSKLKNL+VLLAG+NDFT+LPSELLFL
Sbjct  73   LNSLTSLPEFIGECKSLEQLNLFGNDLTTLPSTFSKLKNLQVLLAGSNDFTVLPSELLFL  132

Query  119  PLIKILYVDRNKLTLTETDVeilaslssleeldlslnsGIKALPFNYEKLVNLINLKRLN  178
            P IK LYVDRN+LTLTETDVEILASLSSLEELDL+ NSGI  LPFNYEKL NL  LKRLN
Sbjct  133  PSIKTLYVDRNRLTLTETDVEILASLSSLEELDLNQNSGIGTLPFNYEKLRNLTGLKRLN  192

Query  179  IKKTSLKGEDADKLQAILPNTKIDY  203
            IKKTSLKGEDA KLQAILPN KIDY
Sbjct  193  IKKTSLKGEDAGKLQAILPNAKIDY  217


>EMF79993.1 leucine rich repeat protein [Leptospira weilii serovar Topaz
```

```
str. LT2116]
Length=185

 Score = 294 bits (753),  Expect = 1e-98, Method: Compositional matrix adjust.
 Identities = 167/185 (90%), Positives = 177/185 (96%), Gaps = 0/185 (0%)

Query  19   LGEAKAKPELVQTLDFGMQKLSTVPEEVCGFPNLTKLDLRLNSLTFLPEFIGECKRLEQL  78
            +GEAKAKPE +QTLDFGMQKLST+PE VCGFPNLTKLDLRLNSLT LPEFIGEC+RLEQL
Sbjct  1    MGEAKAKPESIQTLDFGMQKLSTIPEGVCGFPNLTKLDLRLNSLTSLPEFIGECRRLEQL  60

Query  79   NLFGNDLTTFPSTFSKLKNLKVLLAGNNDFTILPSELLFLPLIKILYVDRNKLTLTETDV  138
            NLFGNDL TFPSTFSKLKNLKVLLAG+NDF+ILPSELLFLPLIKILYVD+NKLTLTETDV
Sbjct  61   NLFGNDLATFPSTFSKLKNLKVLLAGSNDFSILPSELLFLPLIKILYVDQNKLTLTETDV  120

Query  139  eilaslssleeldlslnsGIKALPFNYEKLVNLINLKRLNIKKTSLKGEDADKLQAILPN  198
            EILASLS+LEELDL+LN GIK+LPFNYEKL NL NLKRLNIKKTSLKGEDA KLQAILPN
Sbjct  121  EILASLSNLEELDLNLNLGIKSLPFNYEKLGNLTNLKRLNIKKTSLKGEDAYKLQAILPN  180

Query  199  TKIDY  203
            TKIDY
Sbjct  181  TKIDY  185


>EMY13797.1 leucine rich repeat protein [Leptospira weilii str. Ecochallenge]
Length=185

 Score = 290 bits (742),  Expect = 4e-97, Method: Compositional matrix adjust.
 Identities = 167/185 (90%), Positives = 175/185 (95%), Gaps = 0/185 (0%)

Query  19   LGEAKAKPELVQTLDFGMQKLSTVPEEVCGFPNLTKLDLRLNSLTFLPEFIGECKRLEQL  78
            +GEAKAK E ++TLDFGMQKLSTVPE VCGFPNLTKLDLRLNSLT LPEFIG+CK LEQL
Sbjct  1    MGEAKAKSESIRTLDFGMQKLSTVPEGVCGFPNLTKLDLRLNSLTSLPEFIGDCKNLEQL  60

Query  79   NLFGNDLTTFPSTFSKLKNLKVLLAGNNDFTILPSELLFLPLIKILYVDRNKLTLTETDV  138
            NLFGNDL TFP TFSKLKNLKVLLAG+NDFTILPSELLFLPLIKILYVDRNKLTLTETDV
Sbjct  61   NLFGNDLATFPPTFSKLKNLKVLLAGSNDFTILPSELLFLPLIKILYVDRNKLTLTETDV  120

Query  139  eilaslssleeldlslnsGIKALPFNYEKLVNLINLKRLNIKKTSLKGEDADKLQAILPN  198
            EILASLS+LEELDL+LN GIK+LPFNYEKL NL NLKRLNIKKTSLKGEDADKLQAILPN
Sbjct  121  EILASLSNLEELDLNLNLGIKSLPFNYEKLGNLTNLKRLNIKKTSLKGEDADKLQAILPN  180

Query  199  TKIDY  203
            TKIDY
Sbjct  181  TKIDY  185


>WP_100761136.1 leucine-rich repeat domain-containing protein [Leptospira barantonii]
 PJZ59167.1 hypothetical protein CH367_03875 [Leptospira barantonii]
Length=217

 Score = 289 bits (739),  Expect = 4e-96, Method: Compositional matrix adjust.
 Identities = 165/195 (85%), Positives = 176/195 (90%), Gaps = 0/195 (0%)

Query  9    FDCKKNAEEILGEAKAKPELVQTLDFGMQKLSTVPEEVCGFPNLTKLDLRLNSLTFLPEF  68
             DCKKNAEEIL EAKAKPE VQ LD GMQKL+ VPE VCGFP+LTKLDLRLNSLT LPEF
```

```
Sbjct  23   IDCKKNAEEILEEAKAKPESVQVLDLGMQKLTAVPESVCGFPSLTKLDLRLNSLTSLPEF  82

Query  69   IGECKRLEQLNLFGNDLTTFPSTFSKLKNLKVLLAGNNDFTILPSELLFLPLIKILYVDR  128
            IGECK LEQLNLFGNDLT+ PS+FSKLKNLKVLLAG+ND T+LPSELLFLPLIK LY+D+
Sbjct  83   IGECKNLEQLNLFGNDLTSIPSSFSKLKNLKVLLAGSNDLTVLPSELLFLPLIKTLYLDQ  142

Query  129  NKLTLTETDVeilaslssleeldlslnsGIKALPFNYEKLVNLINLKRLNIKKTSLKGED  188
            NKL L ETDVEILASLS LEELDL+LNSGIKALP NYEKL +L  LKRLNIKKTSLKGED
Sbjct  143  NKLVLNETDVEILASLSGLEELDLNLNSGIKALPSNYEKLKSLTRLKRLNIKKTSLKGED  202

Query  189  ADKLQAILPNTKIDY  203
            ADKLQA+LPNTKIDY
Sbjct  203  ADKLQALLPNTKIDY  217


>WP_123179347.1 leucine-rich repeat domain-containing protein [Leptospira kmetyi]
 AYV55126.1 hypothetical protein EFP84_06105 [Leptospira kmetyi]
Length=217

 Score = 280 bits (716),  Expect = 1e-92, Method: Compositional matrix adjust.
 Identities = 160/195 (82%), Positives = 172/195 (88%), Gaps = 0/195 (0%)

Query  9    FDCKKNAEEILGEAKAKPELVQTLDFGMQKLSTVPEEVCGFPNLTKLDLRLNSLTFLPEF  68
             DCKKNA E+L EAKAKPE VQTLD GMQKL+ VPE VC FPNLTKLDLRLNSLT LP+
Sbjct  23   IDCKKNAGEVLEEAKAKPESVQTLDLGMQKLTAVPEGVCAFPNLTKLDLRLNSLTVLPDS  82

Query  69   IGECKRLEQLNLFGNDLTTFPSTFSKLKNLKVLLAGNNDFTILPSELLFLPLIKILYVDR  128
            IGECK LEQLNLFGNDLT+ PS+FSKLKNLKVLLAG+ND T+LPSELLFLP I+ LY+D+
Sbjct  83   IGECKNLEQLNLFGNDLTSIPSSFSKLKNLKVLLAGSNDLTVLPSELLFLPSIRTLYLDQ  142

Query  129  NKLTLTETDVeilaslssleeldlslnsGIKALPFNYEKLVNLINLKRLNIKKTSLKGED  188
            NKL L ETDVEILASL  LEELDL+LNSGIKALP NYEKL +L  LKRLNIKKTSLKGED
Sbjct  143  NKLVLNETDVEILASLPGLEELDLNLNSGIKALPSNYEKLKSLTRLKRLNIKKTSLKGED  202

Query  189  ADKLQAILPNTKIDY  203
            ADKLQAILPNTKIDY
Sbjct  203  ADKLQAILPNTKIDY  217


>WP_100755326.1 leucine-rich repeat domain-containing protein [Leptospira kmetyi]
 PJZ30273.1 hypothetical protein CH378_08580 [Leptospira kmetyi]
Length=217

 Score = 280 bits (715),  Expect = 1e-92, Method: Compositional matrix adjust.
 Identities = 161/195 (83%), Positives = 172/195 (88%), Gaps = 0/195 (0%)

Query  9    FDCKKNAEEILGEAKAKPELVQTLDFGMQKLSTVPEEVCGFPNLTKLDLRLNSLTFLPEF  68
             DCKKNA E+L EAKAKPE VQTLD GMQKL+ VPE VC FPNLTKLDLRLNSLT LP+
Sbjct  23   IDCKKNAGEVLEEAKAKPESVQTLDLGMQKLTAVPEGVCAFPNLTKLDLRLNSLTVLPDS  82

Query  69   IGECKRLEQLNLFGNDLTTFPSTFSKLKNLKVLLAGNNDFTILPSELLFLPLIKILYVDR  128
            IGECK LEQLNLFGNDLT+ PS+FSKLKNLKVLLAG+ND T+LPSELLFLP IK LY+D+
Sbjct  83   IGECKNLEQLNLFGNDLTSIPSSFSKLKNLKVLLAGSNDLTVLPSELLFLPSIKTLYLDQ  142

Query  129  NKLTLTETDVeilaslssleeldlslnsGIKALPFNYEKLVNLINLKRLNIKKTSLKGED  188
```

```
             NKL L ETDVEILASLS LEELDL+LNSGIK LP NYEKL +L  LKRLNIKKTSLKGED
Sbjct  143  NKLALNETDVEILASLSGLEELDLNLNSGIKVLPSNYEKLKSLTRLKRLNIKKTSLKGED  202

Query  189  ADKLQAILPNTKIDY  203
             ADKLQAILPNTKIDY
Sbjct  203  ADKLQAILPNTKIDY  217
```

>WP_135607718.1 leucine-rich repeat domain-containing protein [Leptospira kmetyi]
 TGK19466.1 hypothetical protein EHO62_05620 [Leptospira kmetyi]
 TGK32832.1 hypothetical protein EHO66_03605 [Leptospira kmetyi]
Length=217

 Score = 279 bits (713),  Expect = 3e-92, Method: Compositional matrix adjust.
 Identities = 160/195 (82%), Positives = 172/195 (88%), Gaps = 0/195 (0%)

```
Query  9    FDCKKNAEEILGEAKAKPELVQTLDFGMQKLSTVPEEVCGFPNLTKLDLRLNSLTFLPEF  68
              DCKKNA E+L EAKAKPE VQTLD GMQKL+ VPE VC FPNLTKLDLRLNSLT LP+
Sbjct  23   IDCKKNAGEVLEEAKAKPESVQTLDLGMQKLTAVPEGVCAFPNLTKLDLRLNSLTVLPDS  82

Query  69   IGECKRLEQLNLFGNDLTTFPSTFSKLKNLKVLLAGNNDFTILPSELLFLPLIKILYVDR  128
             IGECK LEQLNLFGNDLT+ PS+FSKLKNLKVLLAG+ND T+LPSELLFLP I+ LY+D+
Sbjct  83   IGECKNLEQLNLFGNDLTSIPSSFSKLKNLKVLLAGSNDLTVLPSELLFLPSIRTLYLDQ  142

Query  129  NKLTLTETDVeilaslssleeldlslnsGIKALPFNYEKLVNLINLKRLNIKKTSLKGED  188
             NKL L ETDVEILASLS LEELDL+LNSGIK LP NYEKL +L  LKRLNIKKTSLKGED
Sbjct  143  NKLVLNETDVEILASLSGLEELDLNLNSGIKVLPSNYEKLKSLTRLKRLNIKKTSLKGED  202

Query  189  ADKLQAILPNTKIDY  203
             ADKLQAILPNTKIDY
Sbjct  203  ADKLQAILPNTKIDY  217
```

>WP_020775386.1 leucine-rich repeat domain-containing protein [Leptospira alstonii]
 EMJ90581.1 leucine rich repeat protein [Leptospira alstonii serovar Sichuan
str. 79601]
 EQA82096.1 leucine rich repeat protein [Leptospira alstonii serovar Pingchang
str. 80-412]
Length=217

 Score = 278 bits (712),  Expect = 6e-92, Method: Compositional matrix adjust.
 Identities = 163/195 (84%), Positives = 172/195 (88%), Gaps = 0/195 (0%)

```
Query  9    FDCKKNAEEILGEAKAKPELVQTLDFGMQKLSTVPEEVCGFPNLTKLDLRLNSLTFLPEF  68
              DCKKNAEEIL EA  KPE V+ LD GMQKLS+VP+ VCGFPNLT LDLRLNSLT LPEF
Sbjct  23   IDCKKNAEEILREANTKPESVRVLDLGMQKLSSVPDGVCGFPNLTNLDLRLNSLTSLPEF  82

Query  69   IGECKRLEQLNLFGNDLTTFPSTFSKLKNLKVLLAGNNDFTILPSELLFLPLIKILYVDR  128
             IGECK LEQLNLFGNDL TFP++ SKLKNLKVLLAGNNDF  LPSELLFLP IK LY+DR
Sbjct  83   IGECKNLEQLNLFGNDLGTFPASISKLKNLKVLLAGNNDFAALPSELLFLPSIKTLYLDR  142

Query  129  NKLTLTETDVeilaslssleeldlslnsGIKALPFNYEKLVNLINLKRLNIKKTSLKGED  188
             NKLTLTETDVEILASLS LEELDL+LNS IKALP NY+KL NL  LKRLNIKKTSLKGED
Sbjct  143  NKLTLTETDVEILASLSGLEELDLNLNSDIKALPSNYKKLKNLTRLKRLNIKKTSLKGED  202
```

```
Query  189  ADKLQAILPNTKIDY  203
            A+KLQAILPNTKIDY
Sbjct  203  AEKLQAILPNTKIDY  217
```

>WP_010574016.1 leucine-rich repeat domain-containing protein [Leptospira kmetyi]
 EQA55239.1 leucine rich repeat protein [Leptospira kmetyi serovar Malaysia
str. Bejo-Iso9]
Length=217

 Score = 278 bits (711),  Expect = 6e-92, Method: Compositional matrix adjust.
 Identities = 160/195 (82%), Positives = 172/195 (88%), Gaps = 0/195 (0%)

```
Query  9    FDCKKNAEEILGEAKAKPELVQTLDFGMQKLSTVPEEVCGFPNLTKLDLRLNSLTFLPEF  68
             DCKKNA E+L EAKAKPE VQ LD GMQKL+ VPE VC FPNLT+LDLRLNSLT LP+
Sbjct  23   IDCKKNAGEVLEEAKAKPESVQNLDLGMQKLTAVPEGVCAFPNLTQLDLRLNSLTVLPDS  82

Query  69   IGECKRLEQLNLFGNDLTTFPSTFSKLKNLKVLLAGNNDFTILPSELLFLPLIKILYVDR  128
            IGECK LEQLNLFGNDLT+ PS+FSKLKNLKVLLAG+ND T+LPSELLFLP IK LY+D+
Sbjct  83   IGECKNLEQLNLFGNDLTSIPSSFSKLKNLKVLLAGSNDLTVLPSELLFLPSIKTLYLDQ  142

Query  129  NKLTLTETDVeilaslssleeldlslnsGIKALPFNYEKLVNLINLKRLNIKKTSLKGED  188
            NKL L ETDVEILASLS LEELDL+LNSGIKALP NYEKL +L  LKRLNIKKTSLKGED
Sbjct  143  NKLVLNETDVEILASLSGLEELDLNLNSGIKALPSNYEKLKSLTRLKRLNIKKTSLKGED  202

Query  189  ADKLQAILPNTKIDY  203
            ADKLQAILPNTKIDY
Sbjct  203  ADKLQAILPNTKIDY  217
```

>WP_135653950.1 leucine-rich repeat domain-containing protein [Leptospira kmetyi]
 TGL70325.1 hypothetical protein EHQ67_05935 [Leptospira kmetyi]
Length=217

 Score = 278 bits (711),  Expect = 7e-92, Method: Compositional matrix adjust.
 Identities = 159/195 (82%), Positives = 171/195 (88%), Gaps = 0/195 (0%)

```
Query  9    FDCKKNAEEILGEAKAKPELVQTLDFGMQKLSTVPEEVCGFPNLTKLDLRLNSLTFLPEF  68
             DCKKNA E+L EAKAKPE VQTLD GMQKL+ VPE VC FPNLTKLDLRLNSLT LP+
Sbjct  23   IDCKKNAGEVLEEAKAKPESVQTLDLGMQKLTAVPEGVCAFPNLTKLDLRLNSLTVLPDS  82

Query  69   IGECKRLEQLNLFGNDLTTFPSTFSKLKNLKVLLAGNNDFTILPSELLFLPLIKILYVDR  128
            IGECK LEQLNLFGNDLT+ PS+FSKLKNLKVLLAG+ND T+LPSELLFLP I+ LY+D+
Sbjct  83   IGECKNLEQLNLFGNDLTSIPSSFSKLKNLKVLLAGSNDLTVLPSELLFLPSIRTLYLDQ  142

Query  129  NKLTLTETDVeilaslssleeldlslnsGIKALPFNYEKLVNLINLKRLNIKKTSLKGED  188
            NKL L ETDVEILASL  LEELDL+LNSGIKALP NYEKL +L  LKRLNIKKTSLKGED
Sbjct  143  NKLVLNETDVEILASLPGLEELDLNLNSGIKALPSNYEKLKSLTRLKRLNIKKTSLKGED  202

Query  189  ADKLQAILPNTKIDY  203
            ADKLQ ILPNTKIDY
Sbjct  203  ADKLQTILPNTKIDY  217
```

>WP_100738364.1 leucine-rich repeat domain-containing protein [Leptospira kmetyi]

```
 PJZ40049.1 hypothetical protein CH370_17970 [Leptospira kmetyi]
Length=217

 Score = 278 bits (711),  Expect = 7e-92, Method: Compositional matrix adjust.
 Identities = 159/195 (82%), Positives = 171/195 (88%), Gaps = 0/195 (0%)

Query  9    FDCKKNAEEILGEAKAKPELVQTLDFGMQKLSTVPEEVCGFPNLTKLDLRLNSLTFLPEF  68
             DCKKNA E+L EAKAKPE VQTLD GMQKL+ VPE VC FPNLTKLDLRLNSLT LP+
Sbjct  23   IDCKKNAGEVLEEAKAKPESVQTLDLGMQKLTAVPEGVCAFPNLTKLDLRLNSLTVLPDS  82

Query  69   IGECKRLEQLNLFGNDLTTFPSTFSKLKNLKVLLAGNNDFTILPSELLFLPLIKILYVDR  128
            IGECK LEQLNLFGNDLT+ PS+FSKLKNLKVLLAG+ND T+LPSELLFLP I+ LY+D+
Sbjct  83   IGECKNLEQLNLFGNDLTSIPSSFSKLKNLKVLLAGSNDLTVLPSELLFLPSIRTLYLDQ  142

Query  129  NKLTLTETDVeilaslssleeldlslnsGIKALPFNYEKLVNLINLKRLNIKKTSLKGED  188
            NKL L ETDVEILASL  LEELDL+LNSGIK LP NYEKL +L  LKRLNIKKTSLKGED
Sbjct  143  NKLVLNETDVEILASLPGLEELDLNLNSGIKVLPSNYEKLKSLTRLKRLNIKKTSLKGED  202

Query  189  ADKLQAILPNTKIDY  203
            ADKLQAILPNTKIDY
Sbjct  203  ADKLQAILPNTKIDY  217


>MBW0432169.1 hypothetical protein [Leptospira yasudae]
Length=217

 Score = 277 bits (709),  Expect = 1e-91, Method: Compositional matrix adjust.
 Identities = 158/193 (82%), Positives = 174/193 (90%), Gaps = 0/193 (0%)

Query  11   CKKNAEEILGEAKAKPELVQTLDFGMQKLSTVPEEVCGFPNLTKLDLRLNSLTFLPEFIG  70
            CKKN+ EIL EAKAKPE VQ LD GMQKLS++P+ VC FPNLT+LDLRLNSLT LP+FIG
Sbjct  25   CKKNSAEILEEAKAKPESVQILDLGMQKLSSIPDGVCAFPNLTRLDLRLNSLTALPDFIG  84

Query  71   ECKRLEQLNLFGNDLTTFPSTFSKLKNLKVLLAGNNDFTILPSELLFLPLIKILYVDRNK  130
            +C +LEQLN+FGNDL  FPS+FSKLKNLKVLLAG+ND TILPSELLFLPLIK LYVD+NK
Sbjct  85   DCTKLEQLNVFGNDLNAFPSSFSKLKNLKVLLAGSNDLTILPSELLFLPLIKTLYVDQNK  144

Query  131  LTLTETDVeilaslssleeldlslnsGIKALPFNYEKLVNLINLKRLNIKKTSLKGEDAD  190
            LTLTETDVEILASLS+LEELDL+LNS I ALP NY+KL +L  LKRLNIKKTSLKGEDAD
Sbjct  145  LTLTETDVEILASLSALEELDLNLNSKIAALPSNYQKLKSLTRLKRLNIKKTSLKGEDAD  204

Query  191  KLQAILPNTKIDY  203
            KLQAILPNTKIDY
Sbjct  205  KLQAILPNTKIDY  217


>TGN02502.1 hypothetical protein EHR10_00815 [Leptospira yasudae]
Length=217

 Score = 277 bits (709),  Expect = 2e-91, Method: Compositional matrix adjust.
 Identities = 158/193 (82%), Positives = 174/193 (90%), Gaps = 0/193 (0%)

Query  11   CKKNAEEILGEAKAKPELVQTLDFGMQKLSTVPEEVCGFPNLTKLDLRLNSLTFLPEFIG  70
            CKKN+ EIL EAKAKPE VQ LD GMQKLS++P+ VC FPNLT+LDLRLNSLT LP+FIG
Sbjct  25   CKKNSAEILEEAKAKPESVQILDLGMQKLSSIPDGVCAFPNLTRLDLRLNSLTALPDFIG  84
```

```
Query  71   ECKRLEQLNLFGNDLTTFPSTFSKLKNLKVLLAGNNDFTILPSELLFLPLIKILYVDRNK  130
            +C +LEQLN+FGNDL  FPS+FSKLKNLKVLLAG+ND TILPSELLFLPLIK LYVD+NK
Sbjct  85   DCTKLEQLNVFGNDLNAFPSSFSKLKNLKVLLAGSNDLTILPSELLFLPLIKTLYVDQNK  144

Query  131  LTLTETDVeilaslssleeldlslnsGIKALPFNYEKLVNLINLKRLNIKKTSLKGEDAD  190
            LTLTETDVEILASLS+LEELDL+LNS I ALP NY+KL +L  LKRLNIKKTSLKGEDAD
Sbjct  145  LTLTETDVEILASLSALEELDLNLNSKIAALPSNYQKLKSLTRLKRLNIKKTSLKGEDAD  204

Query  191  KLQAILPNTKIDY  203
            KLQAILPNTKIDY
Sbjct  205  KLQAILPNTKIDY  217


>WP_118964127.1 hypothetical protein [Leptospira yasudae]
 RHX95586.1 hypothetical protein DLM76_00905 [Leptospira yasudae]
Length=217

 Score = 276 bits (707),  Expect = 2e-91, Method: Compositional matrix adjust.
 Identities = 159/193 (82%), Positives = 174/193 (90%), Gaps = 0/193 (0%)

Query  11   CKKNAEEILGEAKAKPELVQTLDFGMQKLSTVPEEVCGFPNLTKLDLRLNSLTFLPEFIG  70
            CKKNA EIL EAKAKPE VQ LD GMQKLS++P+ VC FPNLT+LDLRLNSLT LP+FIG
Sbjct  25   CKKNAAEILEEAKAKPESVQILDLGMQKLSSIPDGVCAFPNLTRLDLRLNSLTTLPDFIG  84

Query  71   ECKRLEQLNLFGNDLTTFPSTFSKLKNLKVLLAGNNDFTILPSELLFLPLIKILYVDRNK  130
            +C +LEQLN+FGNDL  FPS+FSKLKNLKVLLAG+ND TILPSELLFLPLIK LYVD+NK
Sbjct  85   DCTKLEQLNVFGNDLNAFPSSFSKLKNLKVLLAGSNDLTILPSELLFLPLIKTLYVDQNK  144

Query  131  LTLTETDVeilaslssleeldlslnsGIKALPFNYEKLVNLINLKRLNIKKTSLKGEDAD  190
            LTLTETDVEILASLS+LEELDL+LNS I ALP NY+KL +L  LKRLNIKKTSLKGEDAD
Sbjct  145  LTLTETDVEILASLSALEELDLNLNSKITALPSNYQKLKSLTRLKRLNIKKTSLKGEDAD  204

Query  191  KLQAILPNTKIDY  203
            KLQAILPNTKIDY
Sbjct  205  KLQAILPNTKIDY  217


>WP_061249228.1 leucine-rich repeat domain-containing protein [Leptospira alstonii]
Length=217

 Score = 276 bits (707),  Expect = 3e-91, Method: Compositional matrix adjust.
 Identities = 162/195 (83%), Positives = 172/195 (88%), Gaps = 0/195 (0%)

Query  9    FDCKKNAEEILGEAKAKPELVQTLDFGMQKLSTVPEEVCGFPNLTKLDLRLNSLTFLPEF  68
             DCKKNAEEIL EA  KPE V+ LD GMQKLS+VP+ VCGFPNLT LDLRLNSLT LPEF
Sbjct  23   IDCKKNAEEILREANTKPESVRVLDLGMQKLSSVPDGVCGFPNLTNLDLRLNSLTSLPEF  82

Query  69   IGECKRLEQLNLFGNDLTTFPSTFSKLKNLKVLLAGNNDFTILPSELLFLPLIKILYVDR  128
            IGECK LEQLNLFGNDL TFP++ SKLKNLKVLLAGNNDF  LPSELLFLP IK LY+DR
Sbjct  83   IGECKNLEQLNLFGNDLGTFPASISKLKNLKVLLAGNNDFAALPSELLFLPSIKTLYLDR  142

Query  129  NKLTLTETDVeilaslssleeldlslnsGIKALPFNYEKLVNLINLKRLNIKKTSLKGED  188
            NKLTLTETDVEILASLS LEELDL+LNS IKALP NY+KL NL  LK+LNIKKTSLKGED
Sbjct  143  NKLTLTETDVEILASLSGLEELDLNLNSDIKALPSNYKKLKNLTRLKKLNIKKTSLKGED  202
```

```
Query  189  ADKLQAILPNTKIDY  203
            A+KLQAILPNTKIDY
Sbjct  203  AEKLQAILPNTKIDY  217
```

>EMY77189.1 leucine rich repeat protein [Leptospira weilii serovar Ranarum str. ICFT]
Length=241

```
 Score = 277 bits (708),  Expect = 4e-91, Method: Compositional matrix adjust.
 Identities = 160/205 (78%), Positives = 178/205 (87%), Gaps = 2/205 (1%)

Query  1    GFVCFTASF--DCKKNAEEILGEAKAKPELVQTLDFGMQKLSTVPEEVCGFPNLTKLDLR  58
            G++ F + F  DCKK AEEILGEAKA P  V  LD GMQKLS++PE +C FPNLTKLDLR
Sbjct  37   GWLFFASIFWIDCKKTAEEILGEAKASPSSVLLLDLGMQKLSSIPEGICSFPNLTKLDLR  96

Query  59   LNSLTFLPEFIGECKRLEQLNLFGNDLTTFPSTFSKLKNLKVLLAGNNDFTILPSELLFL  118
            LNSLT LPE +GECK LEQLNLFGNDLTTFP++FSKLKNLKVLLAGNND T+LPSELLFL
Sbjct  97   LNSLTSLPESVGECKNLEQLNLFGNDLTTFPASFSKLKNLKVLLAGNNDLTVLPSELLFL  156

Query  119  PLIKILYVDRNKLTLTETDVeilaslssleeldlslnsGIKALPFNYEKLVNLINLKRLN  178
            P IK LY+D+NK+TLTETDVEILASLS LEELDL+LN+GIK LP NY KL +L  LK+LN
Sbjct  157  PRIKTLYLDQNKITLTETDVEILASLSELEELDLNLNTGIKTLPSNYGKLKSLARLKKLN  216

Query  179  IKKTSLKGEDADKLQAILPNTKIDY  203
            IKKTSLKGEDA+KLQAILPNTKIDY
Sbjct  217  IKKTSLKGEDAEKLQAILPNTKIDY  241
```

>WP_238730679.1 hypothetical protein [Leptospira sanjuanensis]
MCG6194236.1 hypothetical protein [Leptospira sanjuanensis]
Length=217

```
 Score = 276 bits (706),  Expect = 4e-91, Method: Compositional matrix adjust.
 Identities = 158/193 (82%), Positives = 173/193 (90%), Gaps = 0/193 (0%)

Query  11   CKKNAEEILGEAKAKPELVQTLDFGMQKLSTVPEEVCGFPNLTKLDLRLNSLTFLPEFIG  70
            CKKNA EIL EAKAKPE VQ LD GMQKLS +PE VC +PNLT+LDLRLNSLT LP+FIG
Sbjct  25   CKKNAAEILEEAKAKPESVQILDLGMQKLSAIPEGVCAYPNLTRLDLRLNSLTTLPDFIG  84

Query  71   ECKRLEQLNLFGNDLTTFPSTFSKLKNLKVLLAGNNDFTILPSELLFLPLIKILYVDRNK  130
            +C +LEQLN+FGNDL+ FPS+FSKLKNLKVLLAG+ND TILPSELLFLPLIK LYVD+NK
Sbjct  85   DCTKLEQLNVFGNDLSGFPSSFSKLKNLKVLLAGSNDLTILPSELLFLPLIKTLYVDQNK  144

Query  131  LTLTETDVeilaslssleeldlslnsGIKALPFNYEKLVNLINLKRLNIKKTSLKGEDAD  190
            LTLTETDVEILASLS LEELDL+LNS I ALP NY+KL +L  LKRLNIKKTSLKGEDA+
Sbjct  145  LTLTETDVEILASLSVLEELDLNLNSKITALPSNYQKLKSLTRLKRLNIKKTSLKGEDAE  204

Query  191  KLQAILPNTKIDY  203
            KLQAILPNTKIDY
Sbjct  205  KLQAILPNTKIDY  217
```

>WP_238747060.1 hypothetical protein [Leptospira sanjuanensis]

```
 MCG6168822.1 hypothetical protein [Leptospira sanjuanensis]
Length=217

 Score = 276 bits (705),  Expect = 6e-91, Method: Compositional matrix adjust.
 Identities = 158/193 (82%), Positives = 173/193 (90%), Gaps = 0/193 (0%)

Query  11   CKKNAEEILGEAKAKPELVQTLDFGMQKLSTVPEEVCGFPNLTKLDLRLNSLTFLPEFIG  70
            CKKNA EIL EAKAKPE VQ LD GMQKLS +PE VC +PNLT+LDLRLNSLT LP+FIG
Sbjct  25   CKKNAAEILEEAKAKPESVQILDLGMQKLSAIPEGVCAYPNLTRLDLRLNSLTTLPDFIG  84

Query  71   ECKRLEQLNLFGNDLTTFPSTFSKLKNLKVLLAGNNDFTILPSELLFLPLIKILYVDRNK  130
            +C +LEQLN+FGNDL+ FPS+FSKLKNLKVLLAG+ND TILPSELLFLPLIK LYVD+NK
Sbjct  85   DCTKLEQLNVFGNDLSGFPSSFSKLKNLKVLLAGSNDLTILPSELLFLPLIKTLYVDQNK  144

Query  131  LTLTETDVeilaslssleeldlslnsGIKALPFNYEKLVNLINLKRLNIKKTSLKGEDAD  190
            LTLTETDVEILASLS LEELDL+LNS I ALP NY+KL +L  LKRLNIKKTSLKGEDA+
Sbjct  145  LTLTETDVEILASLSVLEELDLNLNSKITALPSNYQKLKSLTRLKRLNIKKTSLKGEDAE  204

Query  191  KLQAILPNTKIDY  203
            KLQAILPNTKIDY
Sbjct  205  KLQAILPNTKIDY  217


>WP_135574399.1 hypothetical protein [Leptospira yasudae]
 TGL76867.1 hypothetical protein EHQ77_17875 [Leptospira yasudae]
 TGL79657.1 hypothetical protein EHQ72_08705 [Leptospira yasudae]
 TGL83601.1 hypothetical protein EHQ83_12525 [Leptospira yasudae]
Length=217

 Score = 275 bits (704),  Expect = 9e-91, Method: Compositional matrix adjust.
 Identities = 161/202 (80%), Positives = 176/202 (87%), Gaps = 1/202 (0%)

Query  2    FVCFTASFDCKKNAEEILGEAKAKPELVQTLDFGMQKLSTVPEEVCGFPNLTKLDLRLNS  61
             V F A   CKKNA EIL EAKAKPE VQ LD GMQKLS++P+ VC FPNLT+LDLRLNS
Sbjct  17   LVSFLA-VACKKNAAEILEEAKAKPESVQILDLGMQKLSSIPDGVCAFPNLTRLDLRLNS  75

Query  62   LTFLPEFIGECKRLEQLNLFGNDLTTFPSTFSKLKNLKVLLAGNNDFTILPSELLFLPLI  121
            LT LP+FIG+C +LEQLN+FGNDL  FPS+FSKLKNL VLLAG+ND TILPSELLFLPLI
Sbjct  76   LTTLPDFIGDCTKLEQLNVFGNDLNAFPSSFSKLKNLNVLLAGSNDLTILPSELLFLPLI  135

Query  122  KILYVDRNKLTLTETDVeilaslssleeldlslnsGIKALPFNYEKLVNLINLKRLNIKK  181
            K LYVD+NKLTLTETDVEILASLS+LEELDL+LNS I ALP NY+KL +L  LKRLNIKK
Sbjct  136  KTLYVDQNKLTLTETDVEILASLSALEELDLNLNSKIAALPSNYQKLKSLTRLKRLNIKK  195

Query  182  TSLKGEDADKLQAILPNTKIDY  203
            TSLKGEDADKLQAILPNTKIDY
Sbjct  196  TSLKGEDADKLQAILPNTKIDY  217


>WP_135581985.1 hypothetical protein [Leptospira yasudae]
 TGK27116.1 hypothetical protein EHQ05_09670 [Leptospira yasudae]
 TGM08091.1 hypothetical protein EHQ86_03500 [Leptospira yasudae]
Length=217

 Score = 273 bits (698),  Expect = 7e-90, Method: Compositional matrix adjust.
```

```
 Identities = 157/193 (81%), Positives = 172/193 (89%), Gaps = 0/193 (0%)

Query  11   CKKNAEEILGEAKAKPELVQTLDFGMQKLSTVPEEVCGFPNLTKLDLRLNSLTFLPEFIG  70
            CKKNA EIL EAKAK E VQ LD GMQKLS++P+ VC FPNLT+LDLRLNSLT LP+FIG
Sbjct  25   CKKNAAEILEEAKAKSESVQILDLGMQKLSSIPDGVCAFPNLTRLDLRLNSLTALPDFIG  84

Query  71   ECKRLEQLNLFGNDLTTFPSTFSKLKNLKVLLAGNNDFTILPSELLFLPLIKILYVDRNK  130
            +C +LEQLN+FGNDL  FPS+FSKLKNLKVLLAG+ND TILPSELLFLPLIK LYVD+NK
Sbjct  85   DCTKLEQLNVFGNDLNAFPSSFSKLKNLKVLLAGSNDLTILPSELLFLPLIKTLYVDQNK  144

Query  131  LTLTETDVeilaslssleeldlslnsGIKALPFNYEKLVNLINLKRLNIKKTSLKGEDAD  190
            LTLTETDVEILASL +LEELDL+LNS I ALP NY+KL +L  LKRLNIKKTSLKGEDAD
Sbjct  145  LTLTETDVEILASLPALEELDLNLNSKITALPSNYQKLKSLTRLKRLNIKKTSLKGEDAD  204

Query  191  KLQAILPNTKIDY  203
            KLQAILPNTKIDY
Sbjct  205  KLQAILPNTKIDY  217


>WP_118956310.1 hypothetical protein [Leptospira yasudae]
 RHX79626.1 hypothetical protein DLM77_12155 [Leptospira yasudae]
Length=217

 Score = 271 bits (694),  Expect = 2e-89, Method: Compositional matrix adjust.
 Identities = 156/193 (81%), Positives = 171/193 (89%), Gaps = 0/193 (0%)

Query  11   CKKNAEEILGEAKAKPELVQTLDFGMQKLSTVPEEVCGFPNLTKLDLRLNSLTFLPEFIG  70
            CKKNA EIL EAKAK E VQ LD GMQKLS++P+ VC FPNLT+LDLRLNSLT LP+FIG
Sbjct  25   CKKNAAEILEEAKAKSESVQILDLGMQKLSSIPDGVCAFPNLTRLDLRLNSLTALPDFIG  84

Query  71   ECKRLEQLNLFGNDLTTFPSTFSKLKNLKVLLAGNNDFTILPSELLFLPLIKILYVDRNK  130
            +C +LEQLN+FGNDL  FPS+FSKLKNLKVLLAG+ND TILPSELLFLPLIK LYVD+NK
Sbjct  85   DCTKLEQLNVFGNDLNAFPSSFSKLKNLKVLLAGSNDLTILPSELLFLPLIKTLYVDQNK  144

Query  131  LTLTETDVeilaslssleeldlslnsGIKALPFNYEKLVNLINLKRLNIKKTSLKGEDAD  190
            LTLTE DVEILASL +LEELDL+LNS I ALP NY+KL +L  LKRLNIKKTSLKGEDAD
Sbjct  145  LTLTEADVEILASLPALEELDLNLNSKITALPSNYQKLKSLTRLKRLNIKKTSLKGEDAD  204

Query  191  KLQAILPNTKIDY  203
            KLQAILPNTKIDY
Sbjct  205  KLQAILPNTKIDY  217


>WP_004767299.1 hypothetical protein [Leptospira kirschneri]
 EKR10488.1 leucine rich repeat protein [Leptospira kirschneri serovar Valbuzzi
str. 200702274]
Length=217

 Score = 270 bits (689),  Expect = 2e-88, Method: Compositional matrix adjust.
 Identities = 158/194 (81%), Positives = 170/194 (88%), Gaps = 0/194 (0%)

Query  10   DCKKNAEEILGEAKAKPELVQTLDFGMQKLSTVPEEVCGFPNLTKLDLRLNSLTFLPEFI  69
            DCKKNA EIL EAK K E VQ LD GMQKL+++PE +C FPNLT+LDLRLNSL  LP++I
Sbjct  24   DCKKNAVEILEEAKRKSESVQILDLGMQKLTSIPEGICSFPNLTQLDLRLNSLNSLPDWI  83
```

```
Query  70   GECKRLEQLNLFGNDLTTFPSTFSKLKNLKVLLAGNNDFTILPSELLFLPLIKILYVDRN  129
            G CK LEQ+NLFGNDL T PS+FSKLKNLKVLL GNNDF  LPSELLFLPLIKILY+D+N
Sbjct  84   GTCKNLEQINLFGNDLATIPSSFSKLKNLKVLLVGNNDFAFLPSELLFLPLIKILYLDQN  143

Query  130  KLTLTETDVeilaslssleeldlslnsGIKALPFNYEKLVNLINLKRLNIKKTSLKGEDA  189
            KLTLTETDVEILASLSSLEELDL+LNSGIK LP NY KL NL  LKRLNIKKTSLKGEDA
Sbjct  144  KLTLTETDVEILASLSSLEELDLNLNSGIKMLPSNYNKLKNLNRLKRLNIKKTSLKGEDA  203

Query  190  DKLQAILPNTKIDY  203
            DKLQAILPNTKIDY
Sbjct  204  DKLQAILPNTKIDY  217


>WP_004759233.1 hypothetical protein [Leptospira kirschneri]
 EKP03838.1 leucine rich repeat protein [Leptospira kirschneri str. 2008720114]
 EMK16725.1 leucine rich repeat protein [Leptospira kirschneri serovar Bim
str. PUO 1247]
 EMN03438.1 leucine rich repeat protein [Leptospira kirschneri serovar Bim
str. 1051]
 EMN24755.1 leucine rich repeat protein [Leptospira kirschneri serovar Sokoine
str. RM1]
 EPG48343.1 leucine rich repeat protein [Leptospira kirschneri serovar Cynopteri
str. 3522 CT]
Length=217

 Score = 270 bits (689),  Expect = 2e-88, Method: Compositional matrix adjust.
 Identities = 158/194 (81%), Positives = 170/194 (88%), Gaps = 0/194 (0%)

Query  10   DCKKNAEEILGEAKAKPELVQTLDFGMQKLSTVPEEVCGFPNLTKLDLRLNSLTFLPEFI  69
            DCKKNA EIL EAK K E VQ LD GMQKL+++PE +C FPNLT+LDLRLNSL  LP++I
Sbjct  24   DCKKNAVEILEEAKRKSESVQILDLGMQKLTSIPEGICSFPNLTQLDLRLNSLNSLPDWI  83

Query  70   GECKRLEQLNLFGNDLTTFPSTFSKLKNLKVLLAGNNDFTILPSELLFLPLIKILYVDRN  129
            G CK LEQ+NLFGNDL T PS+FSKLKNLKVLL GNNDF  LPSELLFLPLIKILY+D+N
Sbjct  84   GTCKNLEQINLFGNDLATIPSSFSKLKNLKVLLVGNNDFAFLPSELLFLPLIKILYLDQN  143

Query  130  KLTLTETDVeilaslssleeldlslnsGIKALPFNYEKLVNLINLKRLNIKKTSLKGEDA  189
            KLTLTETDVEILASLSSLEELDL+LNSGIK LP NY KL NL  LKRLNIKKTSLKGEDA
Sbjct  144  KLTLTETDVEILASLSSLEELDLNLNSGIKTLPSNYNKLKNLNRLKRLNIKKTSLKGEDA  203

Query  190  DKLQAILPNTKIDY  203
            DKLQAILPNTKIDY
Sbjct  204  DKLQAILPNTKIDY  217


>WP_004750770.1 MULTISPECIES: hypothetical protein [Leptospira]
 EJO71711.1 leucine rich repeat protein [Leptospira kirschneri serovar Grippotyphosa
str. RM52]
 EKO50626.1 leucine rich repeat protein [Leptospira kirschneri str. 200802841]
 EMK05056.1 leucine rich repeat protein [Leptospira kirschneri str. MMD1493]
 EMK06448.1 leucine rich repeat protein [Leptospira kirschneri]
 EMO80710.1 leucine rich repeat protein [Leptospira kirschneri str. 200801774]
Length=217

 Score = 269 bits (687),  Expect = 3e-88, Method: Compositional matrix adjust.
```

```
 Identities = 157/194 (81%), Positives = 170/194 (88%), Gaps = 0/194 (0%)

Query  10   DCKKNAEEILGEAKAKPELVQTLDFGMQKLSTVPEEVCGFPNLTKLDLRLNSLTFLPEFI  69
            DCKKNA EIL EAK K E VQ LD GMQKL+++PE +C FPNLT+LDLRLNSL  LP++I
Sbjct  24   DCKKNAVEILEEAKRKSESVQILDLGMQKLTSIPEGICSFPNLTQLDLRLNSLNSLPDWI  83

Query  70   GECKRLEQLNLFGNDLTTFPSTFSKLKNLKVLLAGNNDFTILPSELLFLPLIKILYVDRN  129
            G CK LEQ+NLFGNDL T PS+FSKLKNLKVLL GNNDF  LPSELLFLPLIKILY+D+N
Sbjct  84   GTCKNLEQINLFGNDLATIPSSFSKLKNLKVLLVGNNDFAFLPSELLFLPLIKILYLDQN  143

Query  130  KLTLTETDVeilaslssleeldlslnsGIKALPFNYEKLVNLINLKRLNIKKTSLKGEDA  189
            KLTLTETDVEILASLSSLEELDL+LNSGIK LP NY KL NL  LKRLNIKKTSL+GEDA
Sbjct  144  KLTLTETDVEILASLSSLEELDLNLNSGIKTLPSNYNKLKNLNRLKRLNIKKTSLRGEDA  203

Query  190  DKLQAILPNTKIDY  203
            DKLQAILPNTKIDY
Sbjct  204  DKLQAILPNTKIDY  217


>WP_004779378.1 hypothetical protein [Leptospira kirschneri]
 EKO60426.1 leucine rich repeat protein [Leptospira kirschneri str. H2]
 EMK25402.1 leucine rich repeat protein [Leptospira kirschneri serovar Bulgarica
str. Nikolaevo]
Length=217

 Score = 269 bits (687),  Expect = 3e-88, Method: Compositional matrix adjust.
 Identities = 157/194 (81%), Positives = 170/194 (88%), Gaps = 0/194 (0%)

Query  10   DCKKNAEEILGEAKAKPELVQTLDFGMQKLSTVPEEVCGFPNLTKLDLRLNSLTFLPEFI  69
            DCKKNA EI+ EAK K E VQ LD GMQKL+++PE +C FPNLT+LDLRLNSL  LP++I
Sbjct  24   DCKKNAVEIIEEAKRKSESVQILDLGMQKLTSIPEGICSFPNLTQLDLRLNSLNSLPDWI  83

Query  70   GECKRLEQLNLFGNDLTTFPSTFSKLKNLKVLLAGNNDFTILPSELLFLPLIKILYVDRN  129
            G CK LEQ+NLFGNDL T PS+FSKLKNLKVLL GNNDF  LPSELLFLPLIKILY+D+N
Sbjct  84   GTCKNLEQINLFGNDLATIPSSFSKLKNLKVLLVGNNDFAFLPSELLFLPLIKILYLDQN  143

Query  130  KLTLTETDVeilaslssleeldlslnsGIKALPFNYEKLVNLINLKRLNIKKTSLKGEDA  189
            KLTLTETDVEILASLSSLEELDL+LNSGIK LP NY KL NL  LKRLNIKKTSLKGEDA
Sbjct  144  KLTLTETDVEILASLSSLEELDLNLNSGIKTLPSNYNKLKNLNRLKRLNIKKTSLKGEDA  203

Query  190  DKLQAILPNTKIDY  203
            DKLQAILPNTKIDY
Sbjct  204  DKLQAILPNTKIDY  217


>WP_016752856.1 hypothetical protein [Leptospira kirschneri]
Length=217

 Score = 268 bits (686),  Expect = 4e-88, Method: Compositional matrix adjust.
 Identities = 157/194 (81%), Positives = 170/194 (88%), Gaps = 0/194 (0%)

Query  10   DCKKNAEEILGEAKAKPELVQTLDFGMQKLSTVPEEVCGFPNLTKLDLRLNSLTFLPEFI  69
            DCKKNA EI+ EAK K E VQ LD GMQKL+++PE +C FPNLT+LDLRLNSL  LP++I
Sbjct  24   DCKKNAVEIIEEAKRKSESVQILDLGMQKLTSIPEGICSFPNLTQLDLRLNSLNSLPDWI  83
```

```
Query  70   GECKRLEQLNLFGNDLTTFPSTFSKLKNLKVLLAGNNDFTILPSELLFLPLIKILYVDRN  129
            G CK LEQ+NLFGNDL T PS+FSKLKNLKVLL GNNDF  LPSELLFLPLIKILY+D+N
Sbjct  84   GTCKNLEQINLFGNDLATIPSSFSKLKNLKVLLVGNNDFAFLPSELLFLPLIKILYLDQN  143

Query  130  KLTLTETDVeilaslssleeldlslnsGIKALPFNYEKLVNLINLKRLNIKKTSLKGEDA  189
            KLTLTETDVEILASLSSLEELDL+LNSGIK LP NY KL NL  LKRLNIKKTSLKGEDA
Sbjct  144  KLTLTETDVEILASLSSLEELDLNLNSGIKTLPSNYNKLKNLNRLKRLNIKKTSLKGEDA  203

Query  190  DKLQAILPNTKIDY  203
            DKLQAILPNTKIDY
Sbjct  204  DKLQAILPNTKIDY  217


>WP_020778543.1 hypothetical protein [Leptospira kirschneri]
 EMJ94579.1 leucine rich repeat protein [Leptospira kirschneri str. JB]
Length=217

 Score = 268 bits (685),  Expect = 7e-88, Method: Compositional matrix adjust.
 Identities = 157/194 (81%), Positives = 170/194 (88%), Gaps = 0/194 (0%)

Query  10   DCKKNAEEILGEAKAKPELVQTLDFGMQKLSTVPEEVCGFPNLTKLDLRLNSLTFLPEFI  69
            DCKKNA EIL EAK K E VQ LD GMQKL+++PE +C FPNLT+LDLRLNSL  LP++I
Sbjct  24   DCKKNAVEILEEAKRKSESVQILDLGMQKLTSIPEGICSFPNLTQLDLRLNSLNSLPDWI  83

Query  70   GECKRLEQLNLFGNDLTTFPSTFSKLKNLKVLLAGNNDFTILPSELLFLPLIKILYVDRN  129
            G CK LEQ+NLFGNDL T PS+FSKLKNLKVLL GNNDF  LPSELLFLPLIKILY+D+N
Sbjct  84   GTCKNLEQINLFGNDLATIPSSFSKLKNLKVLLVGNNDFAFLPSELLFLPLIKILYLDQN  143

Query  130  KLTLTETDVeilaslssleeldlslnsGIKALPFNYEKLVNLINLKRLNIKKTSLKGEDA  189
            KLTLTETDVEIL+SLSSLEELDL+LNSGIK LP NY KL NL  LKRLNIKKTSLKGEDA
Sbjct  144  KLTLTETDVEILSSLSSLEELDLNLNSGIKTLPSNYNKLKNLNRLKRLNIKKTSLKGEDA  203

Query  190  DKLQAILPNTKIDY  203
            DKLQAILPNTKIDY
Sbjct  204  DKLQAILPNTKIDY  217


>WP_082292870.1 hypothetical protein [Leptospira kirschneri]
 OOV43912.1 hypothetical protein B1J93_06785 [Leptospira kirschneri serovar
Pomona]
Length=217

 Score = 268 bits (684),  Expect = 1e-87, Method: Compositional matrix adjust.
 Identities = 156/194 (80%), Positives = 170/194 (88%), Gaps = 0/194 (0%)

Query  10   DCKKNAEEILGEAKAKPELVQTLDFGMQKLSTVPEEVCGFPNLTKLDLRLNSLTFLPEFI  69
            DCKKNA EI+ EAK K E VQ LD GMQKL+++PE +C FPNLT+LDLRLNSL  LP++I
Sbjct  24   DCKKNAVEIIEEAKRKSESVQILDLGMQKLTSIPEGICSFPNLTQLDLRLNSLNSLPDWI  83

Query  70   GECKRLEQLNLFGNDLTTFPSTFSKLKNLKVLLAGNNDFTILPSELLFLPLIKILYVDRN  129
            G CK LEQ+NLFGNDL T PS+FSKLKNLKVLL GNNDF  LPSELLFLPLIKILY+D+N
Sbjct  84   GTCKNLEQINLFGNDLATIPSSFSKLKNLKVLLVGNNDFAFLPSELLFLPLIKILYLDQN  143

Query  130  KLTLTETDVeilaslssleeldlslnsGIKALPFNYEKLVNLINLKRLNIKKTSLKGEDA  189
            KLTLTETDVEIL+SLSSLEELDL+LNSGIK LP NY KL NL  LKRLNIKKTSLKGEDA
```

```
Sbjct  144  KLTLTETDVEILSSLSSLEELDLNLNSGIKTLPSNYNKLKNLNRLKRLNIKKTSLKGEDA  203

Query  190  DKLQAILPNTKIDY  203
            DKLQAILPNTKIDY
Sbjct  204  DKLQAILPNTKIDY  217


>WP_004768364.1 hypothetical protein [Leptospira kirschneri]
 EMO67317.1 leucine rich repeat protein [Leptospira kirschneri str. 200803703]
Length=217

 Score = 267 bits (682),  Expect = 2e-87, Method: Compositional matrix adjust.
 Identities = 156/194 (80%), Positives = 170/194 (88%), Gaps = 0/194 (0%)

Query  10   DCKKNAEEILGEAKAKPELVQTLDFGMQKLSTVPEEVCGFPNLTKLDLRLNSLTFLPEFI  69
            DCK+NA EI+ EAK K E VQ LD GMQKL+++PE +C FPNLT+LDLRLNSL  LP++I
Sbjct  24   DCKENAVEIIEEAKRKSESVQILDLGMQKLTSIPEGICSFPNLTQLDLRLNSLNSLPDWI  83

Query  70   GECKRLEQLNLFGNDLTTFPSTFSKLKNLKVLLAGNNDFTILPSELLFLPLIKILYVDRN  129
            G CK LEQ+NLFGNDL T PS+FSKLKNLKVLL GNNDF  LPSELLFLPLIKILY+D+N
Sbjct  84   GTCKNLEQINLFGNDLATIPSSFSKLKNLKVLLVGNNDFAFLPSELLFLPLIKILYLDQN  143

Query  130  KLTLTETDVeilaslssleeldlslnsGIKALPFNYEKLVNLINLKRLNIKKTSLKGEDA  189
            KLTLTETDVEILASLSSLEELDL+LNSGIK LP NY KL NL  LKRLNIKKTSLKGEDA
Sbjct  144  KLTLTETDVEILASLSSLEELDLNLNSGIKTLPSNYNKLKNLNRLKRLNIKKTSLKGEDA  203

Query  190  DKLQAILPNTKIDY  203
            DKLQAILPNTKIDY
Sbjct  204  DKLQAILPNTKIDY  217


>WP_004760926.1 hypothetical protein [Leptospira kirschneri]
 EKQ84072.1 leucine rich repeat protein [Leptospira kirschneri serovar Grippotyphosa
str. Moskva]
 OOV50381.1 hypothetical protein B1J94_00195 [Leptospira kirschneri serovar
Grippotyphosa]
Length=217

 Score = 266 bits (681),  Expect = 2e-87, Method: Compositional matrix adjust.
 Identities = 157/194 (81%), Positives = 169/194 (87%), Gaps = 0/194 (0%)

Query  10   DCKKNAEEILGEAKAKPELVQTLDFGMQKLSTVPEEVCGFPNLTKLDLRLNSLTFLPEFI  69
            DCKKNA EIL EAK K E VQ LD GMQKL+++PE +C FPNLT+LDLRLNSL  L ++I
Sbjct  24   DCKKNAVEILEEAKRKSESVQILDLGMQKLTSIPEGICSFPNLTQLDLRLNSLNSLTDWI  83

Query  70   GECKRLEQLNLFGNDLTTFPSTFSKLKNLKVLLAGNNDFTILPSELLFLPLIKILYVDRN  129
            G CK LEQ+NLFGNDL T PS+FSKLKNLKVLL GNNDF  LPSELLFLPLIKILY+D+N
Sbjct  84   GTCKNLEQINLFGNDLATIPSSFSKLKNLKVLLVGNNDFAFLPSELLFLPLIKILYLDQN  143

Query  130  KLTLTETDVeilaslssleeldlslnsGIKALPFNYEKLVNLINLKRLNIKKTSLKGEDA  189
            KLTLTETDVEILASLSSLEELDL+LNSGIK LP NY KL NL  LKRLNIKKTSLKGEDA
Sbjct  144  KLTLTETDVEILASLSSLEELDLNLNSGIKTLPSNYNKLKNLNRLKRLNIKKTSLKGEDA  203

Query  190  DKLQAILPNTKIDY  203
            DKLQAILPNTKIDY
```

```
Sbjct  204  DKLQAILPNTKIDY  217


>WP_069608131.1 hypothetical protein [Leptospira tipperaryensis]
 AOP34913.1 hypothetical protein A0128_14300 [Leptospira tipperaryensis]
Length=217

 Score = 261 bits (667),  Expect = 4e-85, Method: Compositional matrix adjust.
 Identities = 151/193 (78%), Positives = 165/193 (85%), Gaps = 0/193 (0%)

Query  11   CKKNAEEILGEAKAKPELVQTLDFGMQKLSTVPEEVCGFPNLTKLDLRLNSLTFLPEFIG  70
            CKKNAEEIL EAKA P  V  LD GMQKL+ +PE  CGFPNL +LDLRLNSL  LP+ +G
Sbjct  25   CKKNAEEILNEAKANPASVTILDLGMQKLTAIPEGACGFPNLKRLDLRLNSLAALPDSLG  84

Query  71   ECKRLEQLNLFGNDLTTFPSTFSKLKNLKVLLAGNNDFTILPSELLFLPLIKILYVDRNK  130
            ECK +EQLN+FGNDLTTFPS  SKLKNLKVLLAGNND T LPSELLFLP IK +Y+D+NK
Sbjct  85   ECKSVEQLNVFGNDLTTFPSELSKLKNLKVLLAGNNDLTNLPSELLFLPEIKTIYMDQNK  144

Query  131  LTLTETDVeilaslssleeldlslnsGIKALPFNYEKLVNLINLKRLNIKKTSLKGEDAD  190
            LTLTETDV+ILASLS+LEELDL+LNSGIK LP NY KL NL  LKRLNIKKTSLKGEDA+
Sbjct  145  LTLTETDVDILASLSNLEELDLNLNSGIKVLPANYTKLKNLTRLKRLNIKKTSLKGEDAE  204

Query  191  KLQAILPNTKIDY  203
            KLQAILP TKIDY
Sbjct  205  KLQAILPKTKIDY  217


>WP_205279762.1 hypothetical protein [Leptospira ainlahdjerensis]
 MBM9577638.1 hypothetical protein [Leptospira ainlahdjerensis]
Length=217

 Score = 261 bits (667),  Expect = 4e-85, Method: Compositional matrix adjust.
 Identities = 150/193 (78%), Positives = 164/193 (85%), Gaps = 0/193 (0%)

Query  11   CKKNAEEILGEAKAKPELVQTLDFGMQKLSTVPEEVCGFPNLTKLDLRLNSLTFLPEFIG  70
            CKKNAEEIL EAKA P  V  LD GMQKL+ +PE  CGFPNL +LDLRLNSL  LP+ +G
Sbjct  25   CKKNAEEILNEAKANPASVTILDLGMQKLTAIPEGACGFPNLKRLDLRLNSLAALPDSLG  84

Query  71   ECKRLEQLNLFGNDLTTFPSTFSKLKNLKVLLAGNNDFTILPSELLFLPLIKILYVDRNK  130
            ECK +EQLN+FGNDLTTFPS  SKLKNLKVLLAGNND T LPSELLFLP IK +Y+D+NK
Sbjct  85   ECKSVEQLNVFGNDLTTFPSALSKLKNLKVLLAGNNDLTNLPSELLFLPEIKTIYMDQNK  144

Query  131  LTLTETDVeilaslssleeldlslnsGIKALPFNYEKLVNLINLKRLNIKKTSLKGEDAD  190
            LTLTETDV+ILASLS+LEELDL+LN GIK LP NY KL NL  LKRLNIKKTSLKGEDA+
Sbjct  145  LTLTETDVDILASLSNLEELDLNLNVGIKILPANYTKLKNLTRLKRLNIKKTSLKGEDAE  204

Query  191  KLQAILPNTKIDY  203
            KLQAILP TKIDY
Sbjct  205  KLQAILPKTKIDY  217


>WP_232371773.1 hypothetical protein [Leptospira ainazelensis]
Length=199

 Score = 258 bits (658),  Expect = 5e-84, Method: Compositional matrix adjust.
```

```
 Identities = 149/193 (77%), Positives = 166/193 (86%), Gaps = 0/193 (0%)

Query  11   CKKNAEEILGEAKAKPELVQTLDFGMQKLSTVPEEVCGFPNLTKLDLRLNSLTFLPEFIG  70
            CKKN EEIL EA AK + V  LD GMQKL+++P+ VC FPNL +LDLRLNSL  LP+FIG
Sbjct  7    CKKNVEEILNEANAKADSVTILDLGMQKLTSIPDGVCKFPNLKRLDLRLNSLASLPDFIG  66

Query  71   ECKRLEQLNLFGNDLTTFPSTFSKLKNLKVLLAGNNDFTILPSELLFLPLIKILYVDRNK  130
            ECK +EQLN+FGNDLTTFPS  SKLKNLKV LAGNND T LPSELLFLP IK +Y+D+NK
Sbjct  67   ECKSVEQLNVFGNDLTTFPSALSKLKNLKVFLAGNNDLTNLPSELLFLPEIKTIYMDQNK  126

Query  131  LTLTETDVeilaslssleeldlslnsGIKALPFNYEKLVNLINLKRLNIKKTSLKGEDAD  190
            LTLTETDV+ILASLS+LEELDL+LNSGIK+LP NY KL NL  LKRLNIKKTSLKGEDA+
Sbjct  127  LTLTETDVDILASLSNLEELDLNLNSGIKSLPSNYTKLKNLTRLKRLNIKKTSLKGEDAE  186

Query  191  KLQAILPNTKIDY  203
            KLQAILP TKIDY
Sbjct  187  KLQAILPKTKIDY  199


>MBM9502464.1 hypothetical protein [Leptospira ainazelensis]
Length=217

 Score = 258 bits (659),  Expect = 6e-84, Method: Compositional matrix adjust.
 Identities = 149/193 (77%), Positives = 166/193 (86%), Gaps = 0/193 (0%)

Query  11   CKKNAEEILGEAKAKPELVQTLDFGMQKLSTVPEEVCGFPNLTKLDLRLNSLTFLPEFIG  70
            CKKN EEIL EA AK + V  LD GMQKL+++P+ VC FPNL +LDLRLNSL  LP+FIG
Sbjct  25   CKKNVEEILNEANAKADSVTILDLGMQKLTSIPDGVCKFPNLKRLDLRLNSLASLPDFIG  84

Query  71   ECKRLEQLNLFGNDLTTFPSTFSKLKNLKVLLAGNNDFTILPSELLFLPLIKILYVDRNK  130
            ECK +EQLN+FGNDLTTFPS  SKLKNLKV LAGNND T LPSELLFLP IK +Y+D+NK
Sbjct  85   ECKSVEQLNVFGNDLTTFPSALSKLKNLKVFLAGNNDLTNLPSELLFLPEIKTIYMDQNK  144

Query  131  LTLTETDVeilaslssleeldlslnsGIKALPFNYEKLVNLINLKRLNIKKTSLKGEDAD  190
            LTLTETDV+ILASLS+LEELDL+LNSGIK+LP NY KL NL  LKRLNIKKTSLKGEDA+
Sbjct  145  LTLTETDVDILASLSNLEELDLNLNSGIKSLPSNYTKLKNLTRLKRLNIKKTSLKGEDAE  204

Query  191  KLQAILPNTKIDY  203
            KLQAILP TKIDY
Sbjct  205  KLQAILPKTKIDY  217


>WP_004443975.1 hypothetical protein [Leptospira noguchii]
 EMO90500.1 leucine rich repeat protein [Leptospira noguchii str. 2001034031]
Length=217

 Score = 255 bits (652),  Expect = 7e-83, Method: Compositional matrix adjust.
 Identities = 158/202 (78%), Positives = 174/202 (86%), Gaps = 0/202 (0%)

Query  2    FVCFTASFDCKKNAEEILGEAKAKPELVQTLDFGMQKLSTVPEEVCGFPNLTKLDLRLNS  61
            F+      DCKKNA EIL EAK K E VQ LD GMQKL+++PE VC FPNLT+LDLRLNS
Sbjct  16   FLISIFWIDCKKNAIEILEEAKKKSESVQILDLGMQKLTSIPEGVCSFPNLTQLDLRLNS  75

Query  62   LTFLPEFIGECKRLEQLNLFGNDLTTFPSTFSKLKNLKVLLAGNNDFTILPSELLFLPLI  121
            L+FLP++IG CK LEQ+NLFGNDLTT PS+FSKLKNLKVLL GNNDFT LPSELLFLPLI
```

```
Sbjct  76   LSFLPDWIGTCKNLEQVNLFGNDLTTVPSSFSKLKNLKVLLLGNNDFTFLPSELLFLPLI  135

Query  122  KILYVDRNKLTLTETDVeilaslssleeldlslnsGIKALPFNYEKLVNLINLKRLNIKK  181
            K LY+D+NKLTLTETD+EIL+SLS LEELDL+LN GIK LP NY KL NLI+LKRLNIKK
Sbjct  136  KTLYLDQNKLTLTETDIEILSSLSGLEELDLNLNPGIKILPSNYNKLKNLIHLKRLNIKK  195

Query  182  TSLKGEDADKLQAILPNTKIDY  203
            T LKGEDADKLQAILPNTKIDY
Sbjct  196  TLLKGEDADKLQAILPNTKIDY  217


>WP_004449517.1 hypothetical protein [Leptospira noguchii]
 EMI70452.1 leucine rich repeat protein [Leptospira noguchii str. Bonito]
 TQE73030.1 hypothetical protein FF021_12990 [Leptospira noguchii]
 UOG31535.1 hypothetical protein MAL06_05855 [Leptospira noguchii]
 UOG35182.1 hypothetical protein MAL02_05610 [Leptospira noguchii]
 UOG46091.1 hypothetical protein MAL01_05745 [Leptospira noguchii]
Length=217

 Score = 254 bits (650),  Expect = 1e-82, Method: Compositional matrix adjust.
 Identities = 157/194 (81%), Positives = 172/194 (89%), Gaps = 0/194 (0%)

Query  10   DCKKNAEEILGEAKAKPELVQTLDFGMQKLSTVPEEVCGFPNLTKLDLRLNSLTFLPEFI  69
            DCKKNA EIL EAK K E VQ LD GMQKL+++PE VC FPNLT+LDLRLNSL+FLP++I
Sbjct  24   DCKKNAVEILEEAKKKSESVQILDLGMQKLTSIPEGVCSFPNLTQLDLRLNSLSFLPDWI  83

Query  70   GECKRLEQLNLFGNDLTTFPSTFSKLKNLKVLLAGNNDFTILPSELLFLPLIKILYVDRN  129
            G CK LEQ+NLFGNDLTT PS+FSKLKNLKVLL GNNDFT LPSELLFLPLIK LY+D+N
Sbjct  84   GTCKNLEQVNLFGNDLTTVPSSFSKLKNLKVLLLGNNDFTFLPSELLFLPLIKTLYLDQN  143

Query  130  KLTLTETDVeilaslssleeldlslnsGIKALPFNYEKLVNLINLKRLNIKKTSLKGEDA  189
            KLTLTETD+EIL+SLS LEELDL+LNSGIK LP NY KL NL +LKRLNIKKT LKGEDA
Sbjct  144  KLTLTETDIEILSSLSGLEELDLNLNSGIKMLPSNYNKLKNLTHLKRLNIKKTLLKGEDA  203

Query  190  DKLQAILPNTKIDY  203
            DKLQAILPNTKIDY
Sbjct  204  DKLQAILPNTKIDY  217


>WP_004422093.1 hypothetical protein [Leptospira noguchii]
 EKR73675.1 leucine rich repeat protein [Leptospira noguchii str. 2006001870]
 UOG49773.1 hypothetical protein MAL00_05865 [Leptospira noguchii]
Length=217

 Score = 254 bits (650),  Expect = 2e-82, Method: Compositional matrix adjust.
 Identities = 157/194 (81%), Positives = 172/194 (89%), Gaps = 0/194 (0%)

Query  10   DCKKNAEEILGEAKAKPELVQTLDFGMQKLSTVPEEVCGFPNLTKLDLRLNSLTFLPEFI  69
            DCKKNA EIL EAK K E VQ LD GMQKL+++PE VC FPNLT+LDLRLNSL+FLP++I
Sbjct  24   DCKKNAVEILEEAKKKSESVQILDLGMQKLTSIPEGVCSFPNLTQLDLRLNSLSFLPDWI  83

Query  70   GECKRLEQLNLFGNDLTTFPSTFSKLKNLKVLLAGNNDFTILPSELLFLPLIKILYVDRN  129
            G CK LEQ+NLFGNDLTT PS+FSKLKNLKVLL GNNDFT LPSELLFLPLIK LY+D+N
Sbjct  84   GTCKNLEQVNLFGNDLTTVPSSFSKLKNLKVLLLGNNDFTFLPSELLFLPLIKTLYLDQN  143
```

```
Query  130  KLTLTETDVeilaslssleeldlslnsGIKALPFNYEKLVNLINLKRLNIKKTSLKGEDA  189
            KLTLTETD+EIL+SLS LEELDL+LNSGIK LP NY KL NL +LKRLNIKKT LKGEDA
Sbjct  144  KLTLTETDIEILSSLSGLEELDLNLNSGIKILPSNYNKLKNLTHLKRLNIKKTLLKGEDA  203

Query  190  DKLQAILPNTKIDY  203
            DKLQAILPNTKIDY
Sbjct  204  DKLQAILPNTKIDY  217


>WP_004455400.1 hypothetical protein [Leptospira noguchii]
 EMS87372.1 leucine rich repeat protein [Leptospira noguchii str. Hook]
 UOG61555.1 hypothetical protein MAL07_06005 [Leptospira noguchii]
Length=217

 Score = 254 bits (648),  Expect = 2e-82, Method: Compositional matrix adjust.
 Identities = 157/194 (81%), Positives = 172/194 (89%), Gaps = 0/194 (0%)

Query  10   DCKKNAEEILGEAKAKPELVQTLDFGMQKLSTVPEEVCGFPNLTKLDLRLNSLTFLPEFI  69
            DCKKNA EIL EAK K E VQ LD GMQKL+++PE VC FPNLT+LDLRLNSL+FLP++I
Sbjct  24   DCKKNAIEILEEAKKKSESVQILDLGMQKLTSIPEGVCSFPNLTQLDLRLNSLSFLPDWI  83

Query  70   GECKRLEQLNLFGNDLTTFPSTFSKLKNLKVLLAGNNDFTILPSELLFLPLIKILYVDRN  129
            G CK LEQ+NLFGNDLTT PS+FSKLKNLKVLL GNNDFT LPSELLFLPLIK LY+D+N
Sbjct  84   GTCKNLEQVNLFGNDLTTVPSSFSKLKNLKVLLLGNNDFTFLPSELLFLPLIKTLYLDQN  143

Query  130  KLTLTETDVeilaslssleeldlslnsGIKALPFNYEKLVNLINLKRLNIKKTSLKGEDA  189
            KLTLTETD+EIL+SLS LEELDL+LNSGIK LP NY KL NL +LKRLNIKKT LKGEDA
Sbjct  144  KLTLTETDIEILSSLSGLEELDLNLNSGIKILPSNYNKLKNLTHLKRLNIKKTLLKGEDA  203

Query  190  DKLQAILPNTKIDY  203
            DKLQAILPNTKIDY
Sbjct  204  DKLQAILPNTKIDY  217


>WP_243817925.1 hypothetical protein [Leptospira noguchii]
 UOG36928.1 hypothetical protein MAL08_12575 [Leptospira noguchii]
Length=217

 Score = 254 bits (648),  Expect = 3e-82, Method: Compositional matrix adjust.
 Identities = 156/194 (80%), Positives = 171/194 (88%), Gaps = 0/194 (0%)

Query  10   DCKKNAEEILGEAKAKPELVQTLDFGMQKLSTVPEEVCGFPNLTKLDLRLNSLTFLPEFI  69
            DCKKNA EIL EAK K E VQ LD GMQKL+++PE VC FPNLT+LDLRLNSL+FLP++I
Sbjct  24   DCKKNAVEILEEAKKKSESVQILDLGMQKLTSIPEGVCSFPNLTQLDLRLNSLSFLPDWI  83

Query  70   GECKRLEQLNLFGNDLTTFPSTFSKLKNLKVLLAGNNDFTILPSELLFLPLIKILYVDRN  129
            G CK LEQ+NLFGNDLTT PS+FSKLKNLKVLL GNNDFT LPSELLFLPLIK LY+D+N
Sbjct  84   GTCKNLEQVNLFGNDLTTVPSSFSKLKNLKVLLLGNNDFTFLPSELLFLPLIKTLYLDQN  143

Query  130  KLTLTETDVeilaslssleeldlslnsGIKALPFNYEKLVNLINLKRLNIKKTSLKGEDA  189
            KLTLTETD+EIL+SLS LEELDL+LN GIK LP NY KL NL +LKRLNIKKT LKGEDA
Sbjct  144  KLTLTETDIEILSSLSGLEELDLNLNPGIKMLPSNYNKLKNLTHLKRLNIKKTLLKGEDA  203

Query  190  DKLQAILPNTKIDY  203
            DKLQAILPNTKIDY
```

```
Sbjct  204  DKLQAILPNTKIDY  217


>WP_053522720.1 hypothetical protein [Leptospira noguchii]
Length=217

 Score = 254 bits (648),  Expect = 3e-82, Method: Compositional matrix adjust.
 Identities = 156/194 (80%), Positives = 172/194 (89%), Gaps = 0/194 (0%)

Query  10   DCKKNAEEILGEAKAKPELVQTLDFGMQKLSTVPEEVCGFPNLTKLDLRLNSLTFLPEFI  69
            DCKK+A EIL EAK K E VQ LD GMQKL+++PE VC FPNLT+LDLRLNSL+FLP++I
Sbjct  24   DCKKSAVEILEEAKKKSESVQILDLGMQKLTSIPEGVCSFPNLTQLDLRLNSLSFLPDWI  83

Query  70   GECKRLEQLNLFGNDLTTFPSTFSKLKNLKVLLAGNNDFTILPSELLFLPLIKILYVDRN  129
            G CK LEQ+N FGNDLTT PS+FSKLKNLKVLL GNNDFT LPSELLFLPLIKILY+D+N
Sbjct  84   GTCKNLEQVNFFGNDLTTVPSSFSKLKNLKVLLLGNNDFTFLPSELLFLPLIKILYLDQN  143

Query  130  KLTLTETDVeilaslssleeldlslnsGIKALPFNYEKLVNLINLKRLNIKKTSLKGEDA  189
            KLTLTETD+EIL+SLS LEELDL+LNSGIK LP NY KL NL +LKRLNIKKT LKGEDA
Sbjct  144  KLTLTETDIEILSSLSGLEELDLNLNSGIKMLPSNYNKLKNLTHLKRLNIKKTLLKGEDA  203

Query  190  DKLQAILPNTKIDY  203
            DKLQAILPNTKIDY
Sbjct  204  DKLQAILPNTKIDY  217


>WP_004452014.1 hypothetical protein [Leptospira noguchii]
 EMS89273.1 leucine rich repeat protein [Leptospira noguchii str. Cascata]
 UOG57651.1 hypothetical protein MAL03_05830 [Leptospira noguchii]
Length=217

 Score = 253 bits (647),  Expect = 4e-82, Method: Compositional matrix adjust.
 Identities = 157/202 (78%), Positives = 173/202 (86%), Gaps = 0/202 (0%)

Query  2    FVCFTASFDCKKNAEEILGEAKAKPELVQTLDFGMQKLSTVPEEVCGFPNLTKLDLRLNS  61
            F+      DCKKNA EIL EAK K E VQ LD GMQKL+++PE VC FPNLT+LDLRLNS
Sbjct  16   FLISIFWIDCKKNAVEILEEAKKKSESVQILDLGMQKLTSIPEGVCSFPNLTQLDLRLNS  75

Query  62   LTFLPEFIGECKRLEQLNLFGNDLTTFPSTFSKLKNLKVLLAGNNDFTILPSELLFLPLI  121
            L+FLP++IG CK LEQ+NLFGNDLTT PS+FSKLKNLKVLL GNNDFT LPSELLFLPLI
Sbjct  76   LSFLPDWIGTCKNLEQVNLFGNDLTTVPSSFSKLKNLKVLLLGNNDFTFLPSELLFLPLI  135

Query  122  KILYVDRNKLTLTETDVeilaslssleeldlslnsGIKALPFNYEKLVNLINLKRLNIKK  181
            K LY+D+NKLTLTETD+EIL+SLS LEELDL+LN GIK LP NY KL NL +LKRLNIKK
Sbjct  136  KTLYLDQNKLTLTETDIEILSSLSGLEELDLNLNPGIKILPSNYNKLKNLTHLKRLNIKK  195

Query  182  TSLKGEDADKLQAILPNTKIDY  203
            T LKGEDADKLQAILPNTKIDY
Sbjct  196  TLLKGEDADKLQAILPNTKIDY  217


>WP_243827912.1 hypothetical protein [Leptospira noguchii]
 UOG42562.1 hypothetical protein MAL05_05795 [Leptospira noguchii]
Length=217
```

```
 Score = 253 bits (646),  Expect = 5e-82, Method: Compositional matrix adjust.
 Identities = 157/202 (78%), Positives = 173/202 (86%), Gaps = 0/202 (0%)

Query  2    FVCFTASFDCKKNAEEILGEAKAKPELVQTLDFGMQKLSTVPEEVCGFPNLTKLDLRLNS  61
            F+      DCKKNA EIL EAK K E VQ LD GMQKL+++PE VC FPNLT+LDLRLNS
Sbjct  16   FLISIFWIDCKKNAIEILEEAKKKSESVQILDLGMQKLTSIPEGVCSFPNLTQLDLRLNS  75

Query  62   LTFLPEFIGECKRLEQLNLFGNDLTTFPSTFSKLKNLKVLLAGNNDFTILPSELLFLPLI  121
            L+FLP++IG CK LEQ+NLFGNDLTT PS+FSKLKNLKVLL GNNDFT LPSELLFLPLI
Sbjct  76   LSFLPDWIGTCKNLEQVNLFGNDLTTVPSSFSKLKNLKVLLLGNNDFTFLPSELLFLPLI  135

Query  122  KILYVDRNKLTLTETDVeilaslssleeldlslnsGIKALPFNYEKLVNLINLKRLNIKK  181
            K LY+D+NKLTLTETD+EIL+SLS LEELDL+LN GIK LP NY KL NL +LKRLNIKK
Sbjct  136  KTLYLDQNKLTLTETDIEILSSLSGLEELDLNLNPGIKILPSNYNKLKNLTHLKRLNIKK  195

Query  182  TSLKGEDADKLQAILPNTKIDY  203
            T LKGEDADKLQAILPNTKIDY
Sbjct  196  TLLKGEDADKLQAILPNTKIDY  217


>WP_061233070.1 hypothetical protein [Leptospira noguchii]
Length=217

 Score = 253 bits (645),  Expect = 8e-82, Method: Compositional matrix adjust.
 Identities = 157/202 (78%), Positives = 173/202 (86%), Gaps = 0/202 (0%)

Query  2    FVCFTASFDCKKNAEEILGEAKAKPELVQTLDFGMQKLSTVPEEVCGFPNLTKLDLRLNS  61
            F+      DCKK+A EIL EAK K E VQ LD GMQKL+++PE VC FPNLT+LDLRLNS
Sbjct  16   FLISIFWIDCKKSAIEILEEAKRKSESVQILDLGMQKLTSIPEGVCSFPNLTQLDLRLNS  75

Query  62   LTFLPEFIGECKRLEQLNLFGNDLTTFPSTFSKLKNLKVLLAGNNDFTILPSELLFLPLI  121
            L FLP++IG CK LEQ+NLFGNDLTT PS+FSKLKNLKVLL GNNDFT LPSELLFLPLI
Sbjct  76   LNFLPDWIGTCKNLEQVNLFGNDLTTVPSSFSKLKNLKVLLLGNNDFTFLPSELLFLPLI  135

Query  122  KILYVDRNKLTLTETDVeilaslssleeldlslnsGIKALPFNYEKLVNLINLKRLNIKK  181
            KILY+D+NKLTLTETD+EIL+SLS LEELDL+LN GIK LP NY KL NL +LKRLNIKK
Sbjct  136  KILYLDQNKLTLTETDIEILSSLSGLEELDLNLNPGIKILPSNYNKLKNLTHLKRLNIKK  195

Query  182  TSLKGEDADKLQAILPNTKIDY  203
            T LKGEDADKLQAILPNTKIDY
Sbjct  196  TLLKGEDADKLQAILPNTKIDY  217


>WP_017852233.1 hypothetical protein [Leptospira interrogans]
Length=217

 Score = 252 bits (643),  Expect = 1e-81, Method: Compositional matrix adjust.
 Identities = 157/194 (81%), Positives = 171/194 (88%), Gaps = 0/194 (0%)

Query  10   DCKKNAEEILGEAKAKPELVQTLDFGMQKLSTVPEEVCGFPNLTKLDLRLNSLTFLPEFI  69
            DCKKNA EIL EAK K E +Q LD GMQKL+++PE VC FPNLT+LDLRLNSL  LP +I
Sbjct  24   DCKKNAVEILEEAKRKSESIQILDLGMQKLTSIPEGVCSFPNLTQLDLRLNSLNSLPGWI  83

Query  70   GECKRLEQLNLFGNDLTTFPSTFSKLKNLKVLLAGNNDFTILPSELLFLPLIKILYVDRN  129
            G CK LEQ+NLFGNDL T PS+FSKLKNLKVLL GNNDFT LPSELLFLPLIK LY+D+N
```

```
Sbjct  84   GACKNLEQINLFGNDLNTVPSSFSKLKNLKVLLLGNNDFTFLPSELLFLPLIKTLYLDQN  143

Query  130  KLTLTETDVeilaslssleeldlslnsGIKALPFNYEKLVNLINLKRLNIKKTSLKGEDA  189
            KLTLTETDVEILASLSSLEELDL+LNSGIKALP NY KL NL +LKRLNIKKTSLKGEDA
Sbjct  144  KLTLTETDVEILASLSSLEELDLNLNSGIKALPSNYNKLKNLTHLKRLNIKKTSLKGEDA  203

Query  190  DKLQAILPNTKIDY  203
            DKLQA+LPNT+IDY
Sbjct  204  DKLQAVLPNTRIDY  217


>WP_061241709.1 hypothetical protein [Leptospira interrogans]
Length=217

 Score = 251 bits (641),  Expect = 3e-81, Method: Compositional matrix adjust.
 Identities = 157/194 (81%), Positives = 170/194 (88%), Gaps = 0/194 (0%)

Query  10   DCKKNAEEILGEAKAKPELVQTLDFGMQKLSTVPEEVCGFPNLTKLDLRLNSLTFLPEFI  69
            DCKKNA EIL EAK K E +Q LD GMQKL+++PE VC FPNLT+LDLRLNSL  LP +I
Sbjct  24   DCKKNAVEILEEAKRKSESIQILDLGMQKLTSIPEGVCSFPNLTQLDLRLNSLNSLPGWI  83

Query  70   GECKRLEQLNLFGNDLTTFPSTFSKLKNLKVLLAGNNDFTILPSELLFLPLIKILYVDRN  129
            G CK LEQ+NLFGNDL T PS+FSKLKNLKVLL GNNDFT LPSELLFLPLIK LY+D+N
Sbjct  84   GACKNLEQINLFGNDLNTVPSSFSKLKNLKVLLLGNNDFTFLPSELLFLPLIKTLYLDQN  143

Query  130  KLTLTETDVeilaslssleeldlslnsGIKALPFNYEKLVNLINLKRLNIKKTSLKGEDA  189
            KLTLTETDVEILASLSSLEELDL+LNSGIK LP NY KL NL +LKRLNIKKTSLKGEDA
Sbjct  144  KLTLTETDVEILASLSSLEELDLNLNSGIKVLPSNYNKLKNLTHLKRLNIKKTSLKGEDA  203

Query  190  DKLQAILPNTKIDY  203
            DKLQA+LPNTKIDY
Sbjct  204  DKLQAVLPNTKIDY  217


>WP_001010180.1 hypothetical protein [Leptospira interrogans]
 EMJ57593.1 leucine rich repeat protein [Leptospira interrogans serovar Valbuzzi
str. Duyster]
 ENO74053.1 leucine rich repeat protein [Leptospira interrogans serovar Valbuzzi
str. Valbuzzi]
Length=217

 Score = 251 bits (641),  Expect = 3e-81, Method: Compositional matrix adjust.
 Identities = 157/194 (81%), Positives = 171/194 (88%), Gaps = 0/194 (0%)

Query  10   DCKKNAEEILGEAKAKPELVQTLDFGMQKLSTVPEEVCGFPNLTKLDLRLNSLTFLPEFI  69
            DCKKNA EIL EAK K E +Q LD GMQKL+++PE VC FPNLT+LDLRLNSL  LP +I
Sbjct  24   DCKKNAVEILEEAKRKSESIQILDLGMQKLTSIPEGVCSFPNLTQLDLRLNSLNSLPGWI  83

Query  70   GECKRLEQLNLFGNDLTTFPSTFSKLKNLKVLLAGNNDFTILPSELLFLPLIKILYVDRN  129
            G CK LEQ+NLFGNDL T PS+FSKLKNLKVLL GNNDFT LPSELLFLPLIK LY+D+N
Sbjct  84   GACKNLEQINLFGNDLNTVPSSFSKLKNLKVLLLGNNDFTFLPSELLFLPLIKTLYLDQN  143

Query  130  KLTLTETDVeilaslssleeldlslnsGIKALPFNYEKLVNLINLKRLNIKKTSLKGEDA  189
            KLTLTETDVEILASLSSLEELDL+LNSGIK LP NY KL NLI+LKRLNIKKTSLKG+DA
Sbjct  144  KLTLTETDVEILASLSSLEELDLNLNSGIKVLPSNYNKLKNLIHLKRLNIKKTSLKGKDA  203
```

```
Query  190  DKLQAILPNTKIDY  203
            DKLQA+LPNTKIDY
Sbjct  204  DKLQAVLPNTKIDY  217


>WP_001011126.1 MULTISPECIES: hypothetical protein [Leptospira]
 EJP05270.1 leucine rich repeat protein [Leptospira interrogans serovar Bulgarica
str. Mallika]
 EKO05519.1 leucine rich repeat protein [Leptospira interrogans str. C10069]
 EKO13588.1 leucine rich repeat protein [Leptospira kirschneri str. H1]
 UID84470.1 hypothetical protein J9305_05680 [Leptospira interrogans]
 UML79483.1 hypothetical protein FH602_14425 [Leptospira kirschneri]
Length=217

 Score = 251 bits (641),  Expect = 3e-81, Method: Compositional matrix adjust.
 Identities = 157/194 (81%), Positives = 170/194 (88%), Gaps = 0/194 (0%)

Query  10   DCKKNAEEILGEAKAKPELVQTLDFGMQKLSTVPEEVCGFPNLTKLDLRLNSLTFLPEFI  69
            DCKKNA EIL EAK K E +Q LD GMQKL+++PE VC FPNLT+LDLRLNSL  LP +I
Sbjct  24   DCKKNAVEILEEAKRKSESIQILDLGMQKLTSIPEGVCSFPNLTQLDLRLNSLNSLPGWI  83

Query  70   GECKRLEQLNLFGNDLTTFPSTFSKLKNLKVLLAGNNDFTILPSELLFLPLIKILYVDRN  129
            G CK LEQ+NLFGNDL T PS+FSKLKNLKVLL GNNDFT LPSELLFLPLIK LY+D+N
Sbjct  84   GACKNLEQINLFGNDLNTVPSSFSKLKNLKVLLLGNNDFTFLPSELLFLPLIKTLYLDQN  143

Query  130  KLTLTETDVeilaslssleeldlslnsGIKALPFNYEKLVNLINLKRLNIKKTSLKGEDA  189
            KLTLTETDVEILASLSSLEELDL+LNSGIK LP NY KL NL +LKRLNIKKTSLKGEDA
Sbjct  144  KLTLTETDVEILASLSSLEELDLNLNSGIKVLPSNYNKLKNLTHLKRLNIKKTSLKGEDA  203

Query  190  DKLQAILPNTKIDY  203
            DKLQA+LPNTKIDY
Sbjct  204  DKLQAVLPNTKIDY  217


>WP_118981207.1 hypothetical protein [Leptospira stimsonii]
 RHX88693.1 hypothetical protein DLM78_07170 [Leptospira stimsonii]
 TGK22822.1 hypothetical protein EHO98_05955 [Leptospira stimsonii]
 TGM14994.1 hypothetical protein EHQ90_11000 [Leptospira stimsonii]
Length=217

 Score = 251 bits (640),  Expect = 4e-81, Method: Compositional matrix adjust.
 Identities = 145/195 (74%), Positives = 162/195 (83%), Gaps = 0/195 (0%)

Query  9    FDCKKNAEEILGEAKAKPELVQTLDFGMQKLSTVPEEVCGFPNLTKLDLRLNSLTFLPEF  68
             +CKKN EEIL EA A  + +  LD GMQKL+++PE  C FPNL +LDLRLNSL  LPE
Sbjct  23   VECKKNVEEILNEANASADSIAVLDLGMQKLTSIPEGACKFPNLKRLDLRLNSLASLPES  82

Query  69   IGECKRLEQLNLFGNDLTTFPSTFSKLKNLKVLLAGNNDFTILPSELLFLPLIKILYVDR  128
            +GECK +EQLN+FGNDL TFPS  SKLKNLKVLLAGNND   LPSELLFLP IK +Y+D+
Sbjct  83   LGECKSVEQLNVFGNDLKTFPSALSKLKNLKVLLAGNNDLANLPSELLFLPEIKTIYLDQ  142

Query  129  NKLTLTETDVeilaslssleeldlslnsGIKALPFNYEKLVNLINLKRLNIKKTSLKGED  188
            NKL LTETDV+ILASLSSLEELDLSLN+GIK+LP NY KL NL  LKRLNIKKTSLKGED
Sbjct  143  NKLILTETDVDILASLSSLEELDLSLNTGIKSLPANYTKLKNLTRLKRLNIKKTSLKGED  202
```

```
Query  189  ADKLQAILPNTKIDY  203
            A+KLQAILP TKIDY
Sbjct  203  AEKLQAILPKTKIDY  217
```

```
>WP_004427427.1 hypothetical protein [Leptospira noguchii]
 EMN02249.1 leucine rich repeat protein [Leptospira noguchii str. 2007001578]
 EPE81746.1 leucine rich repeat protein [Leptospira noguchii str. 1993005606]
Length=217

 Score = 251 bits (640),  Expect = 4e-81, Method: Compositional matrix adjust.
 Identities = 154/194 (79%), Positives = 171/194 (88%), Gaps = 0/194 (0%)

Query  10   DCKKNAEEILGEAKAKPELVQTLDFGMQKLSTVPEEVCGFPNLTKLDLRLNSLTFLPEFI  69
            DCKK+A EIL EAK K E VQ LD GMQKL+++PE VC FPNLT+LDLRLNSL+FLP++I
Sbjct  24   DCKKSAIEILEEAKRKSESVQILDLGMQKLTSIPEGVCSFPNLTQLDLRLNSLSFLPDWI  83

Query  70   GECKRLEQLNLFGNDLTTFPSTFSKLKNLKVLLAGNNDFTILPSELLFLPLIKILYVDRN  129
            G CK LEQ+NLFGNDLTT PS+FSKLKNLKVLL GNNDFT LPSELLFLPLIK LY+D+N
Sbjct  84   GTCKNLEQVNLFGNDLTTVPSSFSKLKNLKVLLLGNNDFTFLPSELLFLPLIKTLYLDQN  143

Query  130  KLTLTETDVeilaslssleeldlslnsGIKALPFNYEKLVNLINLKRLNIKKTSLKGEDA  189
            KLTLTETD+EIL+SLS LEELDL+LN GIK LP NY KL NL +LKRLNIKKT L+GEDA
Sbjct  144  KLTLTETDIEILSSLSGLEELDLNLNPGIKMLPSNYNKLKNLTHLKRLNIKKTLLRGEDA  203

Query  190  DKLQAILPNTKIDY  203
            DKLQAILPNTKIDY
Sbjct  204  DKLQAILPNTKIDY  217
```

```
>WP_004438578.1 hypothetical protein [Leptospira noguchii]
 EMO40832.1 leucine rich repeat protein [Leptospira noguchii serovar Autumnalis
str. ZUN142]
Length=217

 Score = 251 bits (640),  Expect = 5e-81, Method: Compositional matrix adjust.
 Identities = 156/202 (77%), Positives = 172/202 (85%), Gaps = 0/202 (0%)

Query  2    FVCFTASFDCKKNAEEILGEAKAKPELVQTLDFGMQKLSTVPEEVCGFPNLTKLDLRLNS  61
            F+      DCKKNA EIL EAK K E VQ LD GMQKL+++PE VC FPNLT+LDLRLNS
Sbjct  16   FLISIFWIDCKKNAVEILEEAKKKSESVQILDLGMQKLTSIPEGVCSFPNLTQLDLRLNS  75

Query  62   LTFLPEFIGECKRLEQLNLFGNDLTTFPSTFSKLKNLKVLLAGNNDFTILPSELLFLPLI  121
            L+FLP++IG CK LEQ+NLFGNDLTT PS+FSKLKNLKVLL GNNDFT LPSELLFLPLI
Sbjct  76   LSFLPDWIGTCKNLEQVNLFGNDLTTVPSSFSKLKNLKVLLLGNNDFTFLPSELLFLPLI  135

Query  122  KILYVDRNKLTLTETDVeilaslssleeldlslnsGIKALPFNYEKLVNLINLKRLNIKK  181
            K LY+D+NKLTLTETD+EIL+SLS LEELDL+LN GIK LP NY KL NL +LKRLNIKK
Sbjct  136  KTLYLDQNKLTLTETDIEILSSLSGLEELDLNLNPGIKILPSNYNKLKNLTHLKRLNIKK  195

Query  182  TSLKGEDADKLQAILPNTKIDY  203
            T LKGEDA KLQAILPNTKIDY
Sbjct  196  TLLKGEDAGKLQAILPNTKIDY  217
```

```
>EMN50877.1 leucine rich repeat protein [Leptospira interrogans str. L1207]
Length=217

 Score = 250 bits (639),  Expect = 6e-81, Method: Compositional matrix adjust.
 Identities = 156/194 (80%), Positives = 170/194 (88%), Gaps = 0/194 (0%)

Query  10   DCKKNAEEILGEAKAKPELVQTLDFGMQKLSTVPEEVCGFPNLTKLDLRLNSLTFLPEFI  69
            DCKKNA EIL EAK K E +Q LD GMQKL+++PE VC FPNLT+LDLRLNSL  LP +I
Sbjct  24   DCKKNAVEILEEAKRKSESIQILDLGMQKLTSIPEGVCSFPNLTQLDLRLNSLNSLPGWI  83

Query  70   GECKRLEQLNLFGNDLTTFPSTFSKLKNLKVLLAGNNDFTILPSELLFLPLIKILYVDRN  129
            G CK LEQ+NLFGNDL T PS+FSKLKNLKVLL GNNDFT LPSELLFLPLIK LY+D+N
Sbjct  84   GACKNLEQINLFGNDLNTVPSSFSKLKNLKVLLLGNNDFTFLPSELLFLPLIKTLYLDQN  143

Query  130  KLTLTETDVeilaslssleeldlslnsGIKALPFNYEKLVNLINLKRLNIKKTSLKGEDA  189
            KLTLTETDVEILASLSSLEELDL+LNSGIK LP NY KL NL +LKRLNIKKTSLKGEDA
Sbjct  144  KLTLTETDVEILASLSSLEELDLNLNSGIKVLPSNYNKLKNLTHLKRLNIKKTSLKGEDA  203

Query  190  DKLQAILPNTKIDY  203
            DKLQA+LPNT+IDY
Sbjct  204  DKLQAVLPNTRIDY  217


>WP_001010181.1 MULTISPECIES: hypothetical protein [Leptospira]
 KAA1269663.1 hypothetical protein C5473_18355 [Leptospira interrogans serovar
Weerasinghe]
 ASV07105.1 hypothetical protein B2G47_15925 [Leptospira interrogans serovar
Canicola]
 EJO80485.1 leucine rich repeat protein [Leptospira interrogans serovar Pomona
str. Kennewicki LC82-25]
 EJP16928.1 leucine rich repeat protein [Leptospira interrogans str. FPW2026]
 EKN96857.1 leucine rich repeat protein [Leptospira interrogans serovar Pomona
str. Pomona]
Length=217

 Score = 250 bits (638),  Expect = 7e-81, Method: Compositional matrix adjust.
 Identities = 156/194 (80%), Positives = 170/194 (88%), Gaps = 0/194 (0%)

Query  10   DCKKNAEEILGEAKAKPELVQTLDFGMQKLSTVPEEVCGFPNLTKLDLRLNSLTFLPEFI  69
            DCKKNA EIL EAK K E +Q LD GMQKL+++PE VC FPNLT+LDLRLNSL  LP +I
Sbjct  24   DCKKNAVEILEEAKRKSESIQILDLGMQKLTSIPEGVCSFPNLTQLDLRLNSLNSLPGWI  83

Query  70   GECKRLEQLNLFGNDLTTFPSTFSKLKNLKVLLAGNNDFTILPSELLFLPLIKILYVDRN  129
            G CK LEQ+NLFGNDL T PS+FSKLKNLKVLL GNNDFT LPSELLFLPLIK LY+D+N
Sbjct  84   GACKNLEQINLFGNDLNTVPSSFSKLKNLKVLLLGNNDFTFLPSELLFLPLIKTLYLDQN  143

Query  130  KLTLTETDVeilaslssleeldlslnsGIKALPFNYEKLVNLINLKRLNIKKTSLKGEDA  189
            KLTLTETDVEILASLSSLEELDL+LNSGIK LP NY KL NL +LKRLNIKKTSLKGEDA
Sbjct  144  KLTLTETDVEILASLSSLEELDLNLNSGIKVLPSNYNKLKNLTHLKRLNIKKTSLKGEDA  203

Query  190  DKLQAILPNTKIDY  203
            DKLQA+LPNT+IDY
Sbjct  204  DKLQAVLPNTRIDY  217
```

```
>WP_001010177.1 hypothetical protein [Leptospira interrogans]
 KAA1287472.1 hypothetical protein C4X99_23350 [Leptospira interrogans serovar
Geyaweera]
 MCR8646250.1 hypothetical protein [Leptospira interrogans serovar Bataviae]
 OAM85608.1 hypothetical protein A1343_17415 [Leptospira interrogans serovar
Bataviae]
 QCO36829.1 hypothetical protein E4412_06120 [Leptospira interrogans]
 QOI37956.1 hypothetical protein Lepto1548_06450 [Leptospira interrogans
serovar Bataviae]
Length=217

 Score = 250 bits (638),  Expect = 8e-81, Method: Compositional matrix adjust.
 Identities = 156/194 (80%), Positives = 170/194 (88%), Gaps = 0/194 (0%)

Query  10   DCKKNAEEILGEAKAKPELVQTLDFGMQKLSTVPEEVCGFPNLTKLDLRLNSLTFLPEFI  69
            DCKKNA EIL EAK K E +Q LD GMQKL+++PE VC FPNLT+LDLRLNSL  LP +I
Sbjct  24   DCKKNAVEILEEAKRKSESIQILDLGMQKLTSIPEGVCSFPNLTQLDLRLNSLNSLPGWI  83

Query  70   GECKRLEQLNLFGNDLTTFPSTFSKLKNLKVLLAGNNDFTILPSELLFLPLIKILYVDRN  129
            G CK LEQ+NLFGNDL T PS+FSKLKNLKVLL GNNDFT LPSELLFLPLIK LY+D+N
Sbjct  84   GACKNLEQINLFGNDLNTVPSSFSKLKNLKVLLLGNNDFTFLPSELLFLPLIKTLYLDQN  143

Query  130  KLTLTETDVeilaslssleeldlslnsGIKALPFNYEKLVNLINLKRLNIKKTSLKGEDA  189
            KLTLTETDVEILASLSSLEELDL+LNSGIK LP NY KL NL +LKRLNIKKTSLKGEDA
Sbjct  144  KLTLTETDVEILASLSSLEELDLNLNSGIKVLPSNYNKLKNLTHLKRLNIKKTSLKGEDA  203

Query  190  DKLQAILPNTKIDY  203
            DKLQA+LPNT+IDY
Sbjct  204  DKLQAVLPNTRIDY  217


>WP_002177142.1 hypothetical protein [Leptospira noguchii]
 EMO54849.1 leucine rich repeat protein [Leptospira noguchii]
 EQA71632.1 leucine rich repeat protein [Leptospira noguchii serovar Panama
str. CZ214]
 MCH1912101.1 hypothetical protein [Leptospira noguchii]
 MCH1915762.1 hypothetical protein [Leptospira noguchii]
 UOG63215.1 hypothetical protein MAL04_12845 [Leptospira noguchii]
Length=217

 Score = 250 bits (638),  Expect = 1e-80, Method: Compositional matrix adjust.
 Identities = 155/194 (80%), Positives = 169/194 (87%), Gaps = 0/194 (0%)

Query  10   DCKKNAEEILGEAKAKPELVQTLDFGMQKLSTVPEEVCGFPNLTKLDLRLNSLTFLPEFI  69
            DCKK+A EIL EAK K E VQ LD GMQKL+++PE VC FPNLT+LDLRLNSL FLP++I
Sbjct  24   DCKKSAIEILEEAKRKSESVQILDLGMQKLTSIPEGVCSFPNLTQLDLRLNSLNFLPDWI  83

Query  70   GECKRLEQLNLFGNDLTTFPSTFSKLKNLKVLLAGNNDFTILPSELLFLPLIKILYVDRN  129
            G CK LEQ+NLFGNDLTT PS+FSKLKNLKVLL GNNDFT LPSELLFLPLIK LY D+N
Sbjct  84   GTCKNLEQVNLFGNDLTTVPSSFSKLKNLKVLLLGNNDFTFLPSELLFLPLIKTLYFDQN  143

Query  130  KLTLTETDVeilaslssleeldlslnsGIKALPFNYEKLVNLINLKRLNIKKTSLKGEDA  189
            KLTLTETD+EIL+SLS LEELDL+LN GIK LP NY KL NL +LKRLNIKKT LKGEDA
Sbjct  144  KLTLTETDIEILSSLSGLEELDLNLNPGIKILPSNYNKLKNLTHLKRLNIKKTLLKGEDA  203
```

```
Query  190  DKLQAILPNTKIDY  203
            DKLQAILPNTKIDY
Sbjct  204  DKLQAILPNTKIDY  217


>WP_100785841.1 hypothetical protein [Leptospira adleri]
 PJZ53367.1 hypothetical protein CH380_10810 [Leptospira adleri]
 PJZ64012.1 hypothetical protein CH376_00300 [Leptospira adleri]
Length=218

 Score = 249 bits (637),  Expect = 1e-80, Method: Compositional matrix adjust.
 Identities = 144/193 (75%), Positives = 162/193 (84%), Gaps = 0/193 (0%)

Query  11   CKKNAEEILGEAKAKPELVQTLDFGMQKLSTVPEEVCGFPNLTKLDLRLNSLTFLPEFIG  70
            CKKN EEIL EA A  + +  LD GMQKL+++PE VC FPNL +LDLRLNSL  LP+ +G
Sbjct  26   CKKNTEEILNEANANADSIAVLDLGMQKLTSIPEGVCKFPNLKRLDLRLNSLASLPDSLG  85

Query  71   ECKRLEQLNLFGNDLTTFPSTFSKLKNLKVLLAGNNDFTILPSELLFLPLIKILYVDRNK  130
            +CK +EQLN+FGNDL TFPS  SKLKNLKVLLAGNND T LPSELLFLP IK +Y+D+NK
Sbjct  86   DCKSVEQLNVFGNDLKTFPSALSKLKNLKVLLAGNNDLTNLPSELLFLPEIKTIYMDQNK  145

Query  131  LTLTETDVeilaslssleeldlslnsGIKALPFNYEKLVNLINLKRLNIKKTSLKGEDAD  190
            LTLTE DV+ILASLS+LEELDL+LNSGIK+LP NY KL NL  LKRLNIKKTSLKGEDAD
Sbjct  146  LTLTEADVDILASLSNLEELDLNLNSGIKSLPTNYAKLKNLTRLKRLNIKKTSLKGEDAD  205

Query  191  KLQAILPNTKIDY  203
            KLQ ILP TKIDY
Sbjct  206  KLQTILPKTKIDY  218


>WP_135691680.1 hypothetical protein [Leptospira adleri]
 TGM52779.1 hypothetical protein EHQ97_12710 [Leptospira adleri]
Length=218

 Score = 249 bits (635),  Expect = 2e-80, Method: Compositional matrix adjust.
 Identities = 144/193 (75%), Positives = 162/193 (84%), Gaps = 0/193 (0%)

Query  11   CKKNAEEILGEAKAKPELVQTLDFGMQKLSTVPEEVCGFPNLTKLDLRLNSLTFLPEFIG  70
            CKKN EEIL EA A  + +  LD GMQKL+++PE VC FPNL +LDLRLNSL  LP+ +G
Sbjct  26   CKKNTEEILNEANANADSIAVLDLGMQKLTSIPEGVCKFPNLKRLDLRLNSLASLPDSLG  85

Query  71   ECKRLEQLNLFGNDLTTFPSTFSKLKNLKVLLAGNNDFTILPSELLFLPLIKILYVDRNK  130
            +CK +EQLN+FGNDL TFPS  SKLKNLKVLLAGNND T LPSELLFLP IK +Y+D+NK
Sbjct  86   DCKSVEQLNVFGNDLKTFPSALSKLKNLKVLLAGNNDLTNLPSELLFLPEIKTIYMDQNK  145

Query  131  LTLTETDVeilaslssleeldlslnsGIKALPFNYEKLVNLINLKRLNIKKTSLKGEDAD  190
            LTLTETDV+ILASLS+LEELDL+LNSGIK+LP NY KL NL  LKR NIKKTSLKGEDAD
Sbjct  146  LTLTETDVDILASLSNLEELDLNLNSGIKSLPSNYAKLKNLTRLKRFNIKKTSLKGEDAD  205

Query  191  KLQAILPNTKIDY  203
            KLQ ILP TKIDY
Sbjct  206  KLQTILPKTKIDY  218
```

```
>PJZ91020.1 hypothetical protein CH379_21015 [Leptospira ellisii]
 PKA03647.1 hypothetical protein CH375_15745 [Leptospira ellisii]
Length=217

 Score = 248 bits (633),  Expect = 4e-80, Method: Compositional matrix adjust.
 Identities = 143/200 (72%), Positives = 164/200 (82%), Gaps = 0/200 (0%)

Query  4    CFTASFDCKKNAEEILGEAKAKPELVQTLDFGMQKLSTVPEEVCGFPNLTKLDLRLNSLT  63
            C  +  DCKKNAEEIL EA+ K E +  LD GMQKLS++PE +C FPNL +LDLRLNSL+
Sbjct  18   CMLSLSDCKKNAEEILTEAEKKTETITVLDLGMQKLSSLPEGICSFPNLQRLDLRLNSLS  77

Query  64   FLPEFIGECKRLEQLNLFGNDLTTFPSTFSKLKNLKVLLAGNNDFTILPSELLFLPLIKI  123
             L E IG+C  LEQ+NLFGNDL+T PSTFSKLK L+VLL G+NDF  LPSELLFLP I+
Sbjct  78   TLAETIGDCGNLEQINLFGNDLSTLPSTFSKLKRLRVLLMGSNDFKTLPSELLFLPEIRT  137

Query  124  LYVDRNKLTLTETDVeilaslssleeldlslnsGIKALPFNYEKLVNLINLKRLNIKKTS  183
            +Y D+NKL+L ETDVEILASL+ LEELDL+LNS IK LP NY KL NL  LKRLNIKKTS
Sbjct  138  IYADQNKLSLAETDVEILASLTGLEELDLNLNSNIKTLPPNYTKLKNLTRLKRLNIKKTS  197

Query  184  LKGEDADKLQAILPNTKIDY  203
            LKGEDA+KLQAILP+TKIDY
Sbjct  198  LKGEDAEKLQAILPSTKIDY  217


>WP_002188945.1 hypothetical protein [Leptospira interrogans]
 ALE38687.1 leucine-rich-repeat protein [Leptospira interrogans serovar Hardjo
str. Norma]
 ALN99945.1 hypothetical protein LIH_06205 [Leptospira interrogans serovar
Hardjo-prajitno]
 EKO95373.1 leucine rich repeat protein [Leptospira interrogans str. Brem
329]
 MCD1166362.1 hypothetical protein [Leptospira interrogans]
 MCH1885836.1 hypothetical protein [Leptospira interrogans]
Length=217

 Score = 248 bits (633),  Expect = 5e-80, Method: Compositional matrix adjust.
 Identities = 155/194 (80%), Positives = 169/194 (87%), Gaps = 0/194 (0%)

Query  10   DCKKNAEEILGEAKAKPELVQTLDFGMQKLSTVPEEVCGFPNLTKLDLRLNSLTFLPEFI  69
            DCKKNA EIL EAK K E +Q LD GMQKL+++PE VC FPNLT+LDLRLNSL  LP +I
Sbjct  24   DCKKNAVEILEEAKRKSESIQILDLGMQKLTSIPEGVCSFPNLTQLDLRLNSLNSLPGWI  83

Query  70   GECKRLEQLNLFGNDLTTFPSTFSKLKNLKVLLAGNNDFTILPSELLFLPLIKILYVDRN  129
            G CK LEQ+NLFGNDL   PS+FSKLKNLKVLL GNNDFT LPSELLFLPLIK LY+D+N
Sbjct  84   GACKNLEQINLFGNDLNMVPSSFSKLKNLKVLLLGNNDFTFLPSELLFLPLIKTLYLDQN  143

Query  130  KLTLTETDVeilaslssleeldlslnsGIKALPFNYEKLVNLINLKRLNIKKTSLKGEDA  189
            KLTLTETDVEILASLSSLEELDL+LNSGIK LP NY KL NL +LKRLNIKKTSLKGEDA
Sbjct  144  KLTLTETDVEILASLSSLEELDLNLNSGIKVLPSNYNKLKNLTHLKRLNIKKTSLKGEDA  203

Query  190  DKLQAILPNTKIDY  203
            DKLQA+LPNT+IDY
Sbjct  204  DKLQAVLPNTRIDY  217
```

```
>WP_118970865.1 hypothetical protein [Leptospira stimsonii]
 RHX84273.1 hypothetical protein DLM75_23045 [Leptospira stimsonii]
Length=217

 Score = 248 bits (632),  Expect = 6e-80, Method: Compositional matrix adjust.
 Identities = 145/193 (75%), Positives = 160/193 (83%), Gaps = 0/193 (0%)

Query  11   CKKNAEEILGEAKAKPELVQTLDFGMQKLSTVPEEVCGFPNLTKLDLRLNSLTFLPEFIG  70
            CKKN EEIL EA A  + +  LD GMQKL+++PE  C FPNL +LDLRLNSL  LPE  G
Sbjct  25   CKKNVEEILNEANASADSIAVLDLGMQKLTSIPEGACKFPNLKRLDLRLNSLASLPESFG  84

Query  71   ECKRLEQLNLFGNDLTTFPSTFSKLKNLKVLLAGNNDFTILPSELLFLPLIKILYVDRNK  130
            ECK +EQLN+FGNDL TFPS  SKLKNLKVLLAGNND   LPSELLFLP IK +Y+D+NK
Sbjct  85   ECKSVEQLNVFGNDLKTFPSALSKLKNLKVLLAGNNDLANLPSELLFLPEIKTIYLDQNK  144

Query  131  LTLTETDVeilaslssleeldlslnsGIKALPFNYEKLVNLINLKRLNIKKTSLKGEDAD  190
            L LTETDV+ILASLSSLEELDLSLN+GIK+LP NY KL NL  LKRLNIKKTSLKGEDA+
Sbjct  145  LILTETDVDILASLSSLEELDLSLNTGIKSLPANYTKLKNLTLLKRLNIKKTSLKGEDAE  204

Query  191  KLQAILPNTKIDY  203
            KLQAILP TKIDY
Sbjct  205  KLQAILPKTKIDY  217


>WP_243399589.1 hypothetical protein [Leptospira ellisii]
Length=199

 Score = 246 bits (627),  Expect = 2e-79, Method: Compositional matrix adjust.
 Identities = 142/194 (73%), Positives = 162/194 (84%), Gaps = 0/194 (0%)

Query  10   DCKKNAEEILGEAKAKPELVQTLDFGMQKLSTVPEEVCGFPNLTKLDLRLNSLTFLPEFI  69
            DCKKNAEEIL EA+ K E +  LD GMQKLS++PE +C FPNL +LDLRLNSL+ L E I
Sbjct  6    DCKKNAEEILTEAEKKTETITVLDLGMQKLSSLPEGICSFPNLQRLDLRLNSLSTLAETI  65

Query  70   GECKRLEQLNLFGNDLTTFPSTFSKLKNLKVLLAGNNDFTILPSELLFLPLIKILYVDRN  129
            G+C  LEQ+NLFGNDL+T PSTFSKLK L+VLL G+NDF  LPSELLFLP I+ +Y D+N
Sbjct  66   GDCGNLEQINLFGNDLSTLPSTFSKLKRLRVLLMGSNDFKTLPSELLFLPEIRTIYADQN  125

Query  130  KLTLTETDVeilaslssleeldlslnsGIKALPFNYEKLVNLINLKRLNIKKTSLKGEDA  189
            KL+L ETDVEILASL+ LEELDL+LNS IK LP NY KL NL  LKRLNIKKTSLKGEDA
Sbjct  126  KLSLAETDVEILASLTGLEELDLNLNSNIKTLPPNYTKLKNLTRLKRLNIKKTSLKGEDA  185

Query  190  DKLQAILPNTKIDY  203
            +KLQAILP+TKIDY
Sbjct  186  EKLQAILPSTKIDY  199


>WP_135589926.1 leucine-rich repeat domain-containing protein [Leptospira gomenensis]
 TGK28958.1 leucine-rich repeat domain-containing protein [Leptospira gomenensis]
 TGK35419.1 leucine-rich repeat domain-containing protein [Leptospira gomenensis]
 TGK40717.1 leucine-rich repeat domain-containing protein [Leptospira gomenensis]
 TGK68439.1 leucine-rich repeat domain-containing protein [Leptospira gomenensis]
Length=217

 Score = 244 bits (622),  Expect = 2e-78, Method: Compositional matrix adjust.
```

```
 Identities = 136/193 (70%), Positives = 160/193 (83%), Gaps = 0/193 (0%)

Query  11   CKKNAEEILGEAKAKPELVQTLDFGMQKLSTVPEEVCGFPNLTKLDLRLNSLTFLPEFIG  70
            CKKNAEEIL +A+A PE V  LD GMQKL++ P  +C FPNL++LDLRLNSL+ L E IG
Sbjct  25   CKKNAEEILKDAEASPETVTVLDLGMQKLTSTPNGICSFPNLSRLDLRLNSLSSLAETIG  84

Query  71   ECKRLEQLNLFGNDLTTFPSTFSKLKNLKVLLAGNNDFTILPSELLFLPLIKILYVDRNK  130
            +CK LEQ+NLFGNDL+ FPS+FSKLK L+V+L G+NDF   PSE LFLP I+ +Y D+N+
Sbjct  85   DCKNLEQINLFGNDLSAFPSSFSKLKRLRVILMGSNDFKTFPSEFLFLPEIRTIYADQNQ  144

Query  131  LTLTETDVeilaslssleeldlslnsGIKALPFNYEKLVNLINLKRLNIKKTSLKGEDAD  190
            L+LTETDVEILASL+ LEELDL+LN  IK+LP NY KL NL  LKRLNIKKTSL+GEDAD
Sbjct  145  LSLTETDVEILASLTGLEELDLNLNRNIKSLPSNYTKLKNLTRLKRLNIKKTSLRGEDAD  204

Query  191  KLQAILPNTKIDY  203
            KLQAILP TKIDY
Sbjct  205  KLQAILPKTKIDY  217


>WP_250180949.1 hypothetical protein [Leptospira interrogans]
 MCL8309742.1 hypothetical protein [Leptospira interrogans]
Length=217

 Score = 232 bits (591),  Expect = 1e-73, Method: Compositional matrix adjust.
 Identities = 156/194 (80%), Positives = 170/194 (88%), Gaps = 0/194 (0%)

Query  10   DCKKNAEEILGEAKAKPELVQTLDFGMQKLSTVPEEVCGFPNLTKLDLRLNSLTFLPEFI  69
            DCKKNA EIL EAK K E +Q LD GMQKL+++PE VC FPNLT+LDLRLNSL  LP +I
Sbjct  24   DCKKNAVEILEEAKRKSESIQILDLGMQKLTSIPEGVCSFPNLTQLDLRLNSLNSLPGWI  83

Query  70   GECKRLEQLNLFGNDLTTFPSTFSKLKNLKVLLAGNNDFTILPSELLFLPLIKILYVDRN  129
            G CK LEQ+NLFGNDL T PS+FSKLKNLKVLL GNNDFT LPSELLFLPL+K LY+D+N
Sbjct  84   GACKNLEQINLFGNDLNTVPSSFSKLKNLKVLLLGNNDFTFLPSELLFLPLLKTLYLDQN  143

Query  130  KLTLTETDVeilaslssleeldlslnsGIKALPFNYEKLVNLINLKRLNIKKTSLKGEDA  189
            KLTLTETDVEILASLSSLEELDL+LNSGIK LP NY KL NL +LKRLNIKKTSLKGEDA
Sbjct  144  KLTLTETDVEILASLSSLEELDLNLNSGIKVLPSNYNKLKNLTHLKRLNIKKTSLKGEDA  203

Query  190  DKLQAILPNTKIDY  203
            DKLQA+LPNTKIDY
Sbjct  204  DKLQAVLPNTKIDY  217


>WP_061272825.1 hypothetical protein [Leptospira interrogans]
Length=217

 Score = 231 bits (590),  Expect = 2e-73, Method: Compositional matrix adjust.
 Identities = 156/194 (80%), Positives = 170/194 (88%), Gaps = 0/194 (0%)

Query  10   DCKKNAEEILGEAKAKPELVQTLDFGMQKLSTVPEEVCGFPNLTKLDLRLNSLTFLPEFI  69
            DCKKNA EIL EAK K E +Q LD GMQKL+++PE VC FPNLT+LDLRLNSL  LP +I
Sbjct  24   DCKKNAVEILEEAKRKSESIQILDLGMQKLTSIPEGVCSFPNLTQLDLRLNSLNSLPGWI  83

Query  70   GECKRLEQLNLFGNDLTTFPSTFSKLKNLKVLLAGNNDFTILPSELLFLPLIKILYVDRN  129
            G CK LEQ+NLFGNDL T PS+FSKLKNLKVLL GNNDFT LPSELLFLPL+K LY+D+N
```

```
Sbjct  84   GACKNLEQINLFGNDLNTVPSSFSKLKNLKVLLLGNNDFTFLPSELLFLPLLKTLYLDQN  143

Query  130  KLTLTETDVeilaslssleeldlslnsGIKALPFNYEKLVNLINLKRLNIKKTSLKGEDA  189
            KLTLTETDVEILASLSSLEELDL+LNSGIK LP NY KL NL +LKRLNIKKTSLKGEDA
Sbjct  144  KLTLTETDVEILASLSSLEELDLNLNSGIKVLPSNYNKLKNLTHLKRLNIKKTSLKGEDA  203

Query  190  DKLQAILPNTKIDY  203
            DKLQA+LPNTKIDY
Sbjct  204  DKLQAVLPNTKIDY  217


>WP_002127204.1 hypothetical protein [Leptospira interrogans]
 EMN78462.1 leucine rich repeat protein [Leptospira interrogans serovar Grippotyphosa
str. UI 12764]
 EMO95466.1 leucine rich repeat protein [Leptospira interrogans str. UI 13372]
Length=217

 Score = 231 bits (590),  Expect = 2e-73, Method: Compositional matrix adjust.
 Identities = 156/194 (80%), Positives = 170/194 (88%), Gaps = 0/194 (0%)

Query  10   DCKKNAEEILGEAKAKPELVQTLDFGMQKLSTVPEEVCGFPNLTKLDLRLNSLTFLPEFI  69
            DCKKNA EIL EAK K E +Q LD GMQKL+++PE VC FPNLT+LDLRLNSL  LP +I
Sbjct  24   DCKKNAVEILEEAKRKSESIQILDLGMQKLTSIPEGVCSFPNLTQLDLRLNSLNSLPGWI  83

Query  70   GECKRLEQLNLFGNDLTTFPSTFSKLKNLKVLLAGNNDFTILPSELLFLPLIKILYVDRN  129
            G CK LEQ+NLFGNDL T PS+FSKLKNLKVLL GNNDFT LPSELLFLPL+K LY+D+N
Sbjct  84   GACKNLEQINLFGNDLNTVPSSFSKLKNLKVLLLGNNDFTFLPSELLFLPLLKTLYLDQN  143

Query  130  KLTLTETDVeilaslssleeldlslnsGIKALPFNYEKLVNLINLKRLNIKKTSLKGEDA  189
            KLTLTETDVEILASLSSLEELDL+LNSGIK LP NY KL NL +LKRLNIKKTSLKGEDA
Sbjct  144  KLTLTETDVEILASLSSLEELDLNLNSGIKVLPSNYNKLKNLTHLKRLNIKKTSLKGEDA  203

Query  190  DKLQAILPNTKIDY  203
            DKLQA+LPNTKIDY
Sbjct  204  DKLQAVLPNTKIDY  217


>WP_061286495.1 hypothetical protein [Leptospira interrogans]
Length=217

 Score = 231 bits (589),  Expect = 2e-73, Method: Compositional matrix adjust.
 Identities = 154/194 (79%), Positives = 170/194 (88%), Gaps = 0/194 (0%)

Query  10   DCKKNAEEILGEAKAKPELVQTLDFGMQKLSTVPEEVCGFPNLTKLDLRLNSLTFLPEFI  69
            DCKKNA EIL EAK K E +Q LD GMQKL+++PE VC FPNLT+LDLRLNSL  LP +I
Sbjct  24   DCKKNAVEILEEAKRKSESIQILDLGMQKLTSIPEGVCSFPNLTQLDLRLNSLNSLPGWI  83

Query  70   GECKRLEQLNLFGNDLTTFPSTFSKLKNLKVLLAGNNDFTILPSELLFLPLIKILYVDRN  129
            G CK LEQ+NLFGNDL T PS+FSKLKNLKVLL GNNDFT LPSELLFLPL+K LY+D+N
Sbjct  84   GACKNLEQINLFGNDLNTVPSSFSKLKNLKVLLLGNNDFTFLPSELLFLPLLKTLYLDQN  143

Query  130  KLTLTETDVeilaslssleeldlslnsGIKALPFNYEKLVNLINLKRLNIKKTSLKGEDA  189
            KLTLTETDVEI+ASLSSLEELDL+LNSGIK LP NY KL NL +LKRLNIKKTSLKGEDA
Sbjct  144  KLTLTETDVEIIASLSSLEELDLNLNSGIKVLPSNYNKLKNLTHLKRLNIKKTSLKGEDA  203
```

```
Query  190  DKLQAILPNTKIDY  203
            DKLQA+LPNT+IDY
Sbjct  204  DKLQAVLPNTRIDY  217


>WP_001010185.1 hypothetical protein [Leptospira interrogans]
 EMN29390.1 leucine rich repeat protein [Leptospira interrogans serovar Pyrogenes
str. L0374]
 EMP06413.1 leucine rich repeat protein [Leptospira interrogans serovar Pyrogenes
str. 200701872]
 EMN63541.1 leucine rich repeat protein [Leptospira interrogans serovar Pyrogenes
str. R168]
 QCO33794.1 hypothetical protein E4414_12465 [Leptospira interrogans]
 QOI35068.1 hypothetical protein LeptoLang_13150 [Leptospira interrogans
serovar Icterohaemorrhagiae]
Length=217

 Score = 231 bits (589),  Expect = 3e-73, Method: Compositional matrix adjust.
 Identities = 155/194 (80%), Positives = 170/194 (88%), Gaps = 0/194 (0%)

Query  10   DCKKNAEEILGEAKAKPELVQTLDFGMQKLSTVPEEVCGFPNLTKLDLRLNSLTFLPEFI  69
            DCKKNA EIL EAK K E +Q LD GMQKL+++PE VC FPNLT+LDLRLNSL  LP +I
Sbjct  24   DCKKNAVEILEEAKRKSESIQILDLGMQKLTSIPEGVCSFPNLTQLDLRLNSLNSLPGWI  83

Query  70   GECKRLEQLNLFGNDLTTFPSTFSKLKNLKVLLAGNNDFTILPSELLFLPLIKILYVDRN  129
            G CK LEQ+NLFGNDL T PS+FSKLKNLKVLL GNNDFT LPSELLFLPL+K LY+D+N
Sbjct  84   GACKNLEQINLFGNDLNTVPSSFSKLKNLKVLLLGNNDFTFLPSELLFLPLLKTLYLDQN  143

Query  130  KLTLTETDVeilaslssleeldlslnsGIKALPFNYEKLVNLINLKRLNIKKTSLKGEDA  189
            KLTLTETDVEILASLSSLEELDL+LNSGIK LP NY KL NL +LKRLNIKKTSLKGEDA
Sbjct  144  KLTLTETDVEILASLSSLEELDLNLNSGIKVLPSNYNKLKNLTHLKRLNIKKTSLKGEDA  203

Query  190  DKLQAILPNTKIDY  203
            DKLQA+LPNT+IDY
Sbjct  204  DKLQAVLPNTRIDY  217


>WP_001010184.1 MULTISPECIES: hypothetical protein [Leptospira]
 EMF71629.1 leucine rich repeat protein [Leptospira interrogans serovar Canicola
str. LT1962]
 EMG20762.1 leucine rich repeat protein [Leptospira interrogans serovar Copenhageni
str. LT2050]
 EMO02625.1 leucine rich repeat protein [Leptospira interrogans serovar
Icterohaemorrhagiae
str. Verdun HP]
 OCA00011.1 Leucine rich repeat protein [Leptospira interrogans serovar
Copenhageni/Icterohaemorrhagiae]
 AAN48523.1 leucine-rich-repeat protein [Leptospira interrogans serovar Lai
str. 56601]
Length=217

 Score = 231 bits (588),  Expect = 3e-73, Method: Compositional matrix adjust.
 Identities = 155/194 (80%), Positives = 170/194 (88%), Gaps = 0/194 (0%)

Query  10   DCKKNAEEILGEAKAKPELVQTLDFGMQKLSTVPEEVCGFPNLTKLDLRLNSLTFLPEFI  69
```

```
             DCKKNA EIL EAK K E +Q LD GMQKL+++PE VC FPNLT+LDLRLNSL  LP +I
Sbjct  24    DCKKNAVEILEEAKRKSESIQILDLGMQKLTSIPEGVCSFPNLTQLDLRLNSLNSLPGWI  83

Query  70    GECKRLEQLNLFGNDLTTFPSTFSKLKNLKVLLAGNNDFTILPSELLFLPLIKILYVDRN  129
             G CK LEQ+NLFGNDL T PS+FSKLKNLKVLL GNNDFT LPSELLFLPL+K LY+D+N
Sbjct  84    GACKNLEQINLFGNDLNTVPSSFSKLKNLKVLLLGNNDFTFLPSELLFLPLLKTLYLDQN  143

Query  130   KLTLTETDVeilaslssleeldlslnsGIKALPFNYEKLVNLINLKRLNIKKTSLKGEDA  189
             KLTLTETDVEILASLSSLEELDL+LNSGIK LP NY KL NL +LKRLNIKKTSLKGEDA
Sbjct  144   KLTLTETDVEILASLSSLEELDLNLNSGIKVLPSNYNKLKNLTHLKRLNIKKTSLKGEDA  203

Query  190   DKLQAILPNTKIDY  203
             DKLQA+LPNT+IDY
Sbjct  204   DKLQAVLPNTRIDY  217


>WP_001010182.1 hypothetical protein [Leptospira interrogans]
 EMY06628.1 leucine rich repeat protein [Leptospira interrogans str. 2002000626]
 EMY23346.1 leucine rich repeat protein [Leptospira interrogans serovar Australis
str. 200703203]
 EKN86156.1 leucine rich repeat protein [Leptospira interrogans str. 2002000624]
 EKQ39337.1 leucine rich repeat protein [Leptospira interrogans str. 2002000621]
 EKQ45586.1 leucine rich repeat protein [Leptospira interrogans str. 2002000623]
Length=217

 Score = 231 bits (588),  Expect = 3e-73, Method: Compositional matrix adjust.
 Identities = 154/194 (79%), Positives = 170/194 (88%), Gaps = 0/194 (0%)

Query  10    DCKKNAEEILGEAKAKPELVQTLDFGMQKLSTVPEEVCGFPNLTKLDLRLNSLTFLPEFI  69
             DCKKNA EIL EAK K E +Q LD GMQKL+++PE VC FPNLT+LDLRLNSL  LP +I
Sbjct  24    DCKKNAVEILEEAKRKSESIQILDLGMQKLTSIPEGVCSFPNLTQLDLRLNSLNSLPGWI  83

Query  70    GECKRLEQLNLFGNDLTTFPSTFSKLKNLKVLLAGNNDFTILPSELLFLPLIKILYVDRN  129
             G CK LEQ+NLFGNDL T PS+FSKLKNLKVLL GNNDFT LPSELLFLPL+K LY+D+N
Sbjct  84    GACKNLEQINLFGNDLNTVPSSFSKLKNLKVLLLGNNDFTFLPSELLFLPLLKTLYLDQN  143

Query  130   KLTLTETDVeilaslssleeldlslnsGIKALPFNYEKLVNLINLKRLNIKKTSLKGEDA  189
             KLTLTETDVEI+ASLSSLEELDL+LNSGIK LP NY KL NL +LKRLNIKKTSLKGEDA
Sbjct  144   KLTLTETDVEIIASLSSLEELDLNLNSGIKVLPSNYNKLKNLTHLKRLNIKKTSLKGEDA  203

Query  190   DKLQAILPNTKIDY  203
             DKLQA+LPNT+IDY
Sbjct  204   DKLQAVLPNTRIDY  217


>WP_001010178.1 hypothetical protein [Leptospira interrogans]
 EMF44105.1 leucine rich repeat protein [Leptospira interrogans serovar Lora
str. TE 1992]
 EMG10292.1 leucine rich repeat protein [Leptospira interrogans serovar Grippotyphosa
str. LT2186]
 EMN70889.1 leucine rich repeat protein [Leptospira interrogans serovar Bataviae
str. UI 08561]
 AJR13823.1 hypothetical protein LIL_11221 [Leptospira interrogans serovar
Linhai str. 56609]
 AKH76669.1 leucine rich repeat protein [Leptospira interrogans serovar Bratislava]
```

```
Length=217

 Score = 231 bits (588),  Expect = 3e-73, Method: Compositional matrix adjust.
 Identities = 155/194 (80%), Positives = 170/194 (88%), Gaps = 0/194 (0%)

Query  10   DCKKNAEEILGEAKAKPELVQTLDFGMQKLSTVPEEVCGFPNLTKLDLRLNSLTFLPEFI  69
            DCKKNA EIL EAK K E +Q LD GMQKL+++PE VC FPNLT+LDLRLNSL  LP +I
Sbjct  24   DCKKNAVEILEEAKRKSESIQILDLGMQKLTSIPEGVCSFPNLTQLDLRLNSLNSLPGWI  83

Query  70   GECKRLEQLNLFGNDLTTFPSTFSKLKNLKVLLAGNNDFTILPSELLFLPLIKILYVDRN  129
            G CK LEQ+NLFGNDL T PS+FSKLKNLKVLL GNNDFT LPSELLFLPL+K LY+D+N
Sbjct  84   GACKNLEQINLFGNDLNTVPSSFSKLKNLKVLLLGNNDFTFLPSELLFLPLLKTLYLDQN  143

Query  130  KLTLTETDVeilaslssleeldlslnsGIKALPFNYEKLVNLINLKRLNIKKTSLKGEDA  189
            KLTLTETDVEILASLSSLEELDL+LNSGIK LP NY KL NL +LKRLNIKKTSLKGEDA
Sbjct  144  KLTLTETDVEILASLSSLEELDLNLNSGIKVLPSNYNKLKNLTHLKRLNIKKTSLKGEDA  203

Query  190  DKLQAILPNTKIDY  203
            DKLQA+LPNT+IDY
Sbjct  204  DKLQAVLPNTRIDY  217


>WP_001010183.1 hypothetical protein [Leptospira interrogans]
 AKP26578.1 leucine rich repeat protein [Leptospira interrogans serovar Manilae]
 AKP30357.1 leucine rich repeat protein [Leptospira interrogans serovar Manilae]
 EYU63041.1 hypothetical protein CI00_16890 [Leptospira interrogans serovar
Manilae]
 SOR62694.1 Leucine rich repeat protein [Leptospira interrogans serovar Manilae]
Length=217

 Score = 231 bits (588),  Expect = 4e-73, Method: Compositional matrix adjust.
 Identities = 155/194 (80%), Positives = 169/194 (87%), Gaps = 0/194 (0%)

Query  10   DCKKNAEEILGEAKAKPELVQTLDFGMQKLSTVPEEVCGFPNLTKLDLRLNSLTFLPEFI  69
            DCKKNA EIL EAK K E +Q LD GMQKL+++PE VC FPNLT+LDLRLNSL  LP +I
Sbjct  24   DCKKNAVEILEEAKRKSESIQILDLGMQKLTSIPEGVCSFPNLTQLDLRLNSLNSLPGWI  83

Query  70   GECKRLEQLNLFGNDLTTFPSTFSKLKNLKVLLAGNNDFTILPSELLFLPLIKILYVDRN  129
            G CK LEQ+NLFGNDL T PS+FSKLKNLKVLL GNNDFT LPSELLFLPL+K LY+D+N
Sbjct  84   GACKNLEQINLFGNDLNTVPSSFSKLKNLKVLLLGNNDFTFLPSELLFLPLLKTLYLDQN  143

Query  130  KLTLTETDVeilaslssleeldlslnsGIKALPFNYEKLVNLINLKRLNIKKTSLKGEDA  189
            KLTLTETDVEILASL SLEELDL+LNSGIK LP NY KL NL +LKRLNIKKTSLKGEDA
Sbjct  144  KLTLTETDVEILASLFSLEELDLNLNSGIKVLPSNYNKLKNLTHLKRLNIKKTSLKGEDA  203

Query  190  DKLQAILPNTKIDY  203
            DKLQA+LPNTKIDY
Sbjct  204  DKLQAVLPNTKIDY  217


>WP_192505591.1 hypothetical protein [Leptospira interrogans]
 QOI42007.1 hypothetical protein Lepto782_06840 [Leptospira interrogans serovar
Canicola]
Length=217
```

```
 Score = 229 bits (584),  Expect = 1e-72, Method: Compositional matrix adjust.
 Identities = 154/194 (79%), Positives = 169/194 (87%), Gaps = 0/194 (0%)

Query  10   DCKKNAEEILGEAKAKPELVQTLDFGMQKLSTVPEEVCGFPNLTKLDLRLNSLTFLPEFI  69
            DCKKNA EIL EAK K E +Q LD GMQKL+++PE VC FPNLT+LDLRLNSL  LP +I
Sbjct  24   DCKKNAVEILEEAKRKSESIQILDLGMQKLTSIPEGVCSFPNLTQLDLRLNSLNSLPGWI  83

Query  70   GECKRLEQLNLFGNDLTTFPSTFSKLKNLKVLLAGNNDFTILPSELLFLPLIKILYVDRN  129
            G CK LEQ+NLFGNDL T PS+FSKLKNLKVLL GNNDFT LPSELLFLPL+K LY+D+N
Sbjct  84   GACKNLEQINLFGNDLNTVPSSFSKLKNLKVLLLGNNDFTFLPSELLFLPLLKTLYLDQN  143

Query  130  KLTLTETDVeilaslssleeldlslnsGIKALPFNYEKLVNLINLKRLNIKKTSLKGEDA  189
            K TLTETDVEILASLSSLEELDL+LNSGIK LP NY KL NL +LKRLNIKKTSLKGEDA
Sbjct  144  KFTLTETDVEILASLSSLEELDLNLNSGIKVLPSNYNKLKNLTHLKRLNIKKTSLKGEDA  203

Query  190  DKLQAILPNTKIDY  203
            DKLQA+LPNT+IDY
Sbjct  204  DKLQAVLPNTRIDY  217


>EMM97254.1 leucine rich repeat protein [Leptospira interrogans serovar Zanoni 
str. LT2156]
Length=188

 Score = 218 bits (555),  Expect = 1e-68, Method: Compositional matrix adjust.
 Identities = 147/187 (79%), Positives = 164/187 (88%), Gaps = 0/187 (0%)

Query  17   EILGEAKAKPELVQTLDFGMQKLSTVPEEVCGFPNLTKLDLRLNSLTFLPEFIGECKRLE  76
            EIL +AK K E +Q LD GMQKL+++PE VC FPNLT+LDLRLNSL  LP +IG CK LE
Sbjct  2    EILEKAKRKSESIQILDLGMQKLTSIPEGVCSFPNLTQLDLRLNSLNSLPGWIGACKNLE  61

Query  77   QLNLFGNDLTTFPSTFSKLKNLKVLLAGNNDFTILPSELLFLPLIKILYVDRNKLTLTET  136
            Q+NLFGNDL T PS+FSKLKNLKVLL GNNDFT LPSELLFLPL+K LY+D+NKLTLTET
Sbjct  62   QINLFGNDLNTVPSSFSKLKNLKVLLLGNNDFTFLPSELLFLPLLKTLYLDQNKLTLTET  121

Query  137  DVeilaslssleeldlslnsGIKALPFNYEKLVNLINLKRLNIKKTSLKGEDADKLQAIL  196
            DVEI+ASLSSLEELDL+LNSGIK LP NY KL NL +LKRLNIKKTSLKGEDADKLQA+L
Sbjct  122  DVEIIASLSSLEELDLNLNSGIKVLPSNYNKLKNLTHLKRLNIKKTSLKGEDADKLQAVL  181

Query  197  PNTKIDY  203
            PNT+IDY
Sbjct  182  PNTRIDY  188


>MBW9228790.1 hypothetical protein [Leptospira interrogans]
Length=186

 Score = 216 bits (551),  Expect = 5e-68, Method: Compositional matrix adjust.
 Identities = 147/186 (79%), Positives = 163/186 (88%), Gaps = 0/186 (0%)

Query  18   ILGEAKAKPELVQTLDFGMQKLSTVPEEVCGFPNLTKLDLRLNSLTFLPEFIGECKRLEQ  77
            +L EAK K E +Q LD GMQKL+++PE VC FPNLT+LDLRLNSL  LP +IG CK LEQ
Sbjct  1    MLEEAKRKSESIQILDLGMQKLTSIPEGVCSFPNLTQLDLRLNSLNSLPGWIGACKNLEQ  60

Query  78   LNLFGNDLTTFPSTFSKLKNLKVLLAGNNDFTILPSELLFLPLIKILYVDRNKLTLTETD  137
```

```
                +NLFGNDL T PS+FSKLKNLKVLL GNNDFT LPSELLFLPL+K LY+D+NKLTLTETD
Sbjct  61     INLFGNDLNTVPSSFSKLKNLKVLLLGNNDFTFLPSELLFLPLLKTLYLDQNKLTLTETD  120

Query  138  VeilaslssleeldlslnsGIKALPFNYEKLVNLINLKRLNIKKTSLKGEDADKLQAILP  197
                VEILASLSSLEELDL+LNSGIK LP NY KL NL +LKRLNIKKTSLKGEDADKLQA+LP
Sbjct  121  VEILASLSSLEELDLNLNSGIKVLPSNYNKLKNLTHLKRLNIKKTSLKGEDADKLQAVLP  180

Query  198  NTKIDY  203
                NT+IDY
Sbjct  181  NTRIDY  186
```

Multiple sequence alignment of the protein rhKU_Sej_LRR_2271 (Genbank: AFV46188.1) and the amino sequences of Leptospira that has the same serovars with rabbit hyperimmune sera in this study using Clustal Omega (https://www.ebi.ac.uk/Tools/msa/clustalo/: access on 11 November 2022)

```
OCA00011.1        --------------MN---------F-LSLKKIFKLYFFLWVSFFGIDCKKNAVEILEEA 36
EMO02625.1        --------------MN---------F-LSLKKIFKLYFFLWVSFFGIDCKKNAVEILEEA 36
EMF71629.1        --------------MN---------F-LSLKKIFKLYFFLWVSFFGIDCKKNAVEILEEA 36
QOI35068.1        --------------MN---------F-LSLKKIFKLYFFLWVSFFVIDCKKNAVEILEEA 36
EMN63541.1        --------------MN---------F-LSLKKIFKLYFFLWVSFFVIDCKKNAVEILEEA 36
EMP06413.1        --------------MN---------F-LSLKKIFKLYFFLWVSFFVIDCKKNAVEILEEA 36
EMN29390.1        --------------MN---------F-LSLKKIFKLYFFLWVSFFVIDCKKNAVEILEEA 36
EMN70889.1        --------------MN---------F-LSLKKIFKIYFFLWVSFFGIDCKKNAVEILEEA 36
EMN78462.1        --------------MN---------F-LSLKKIFKIYFFLWVSFFGIDCKKNAVEILEEA 36
AKH76669.1        --------------MN---------F-LSLKKIFKIYFFLWVSFFGIDCKKNAVEILEEA 36
EMG10292.1        --------------MN---------F-LSLKKIFKIYFFLWVSFFGIDCKKNAVEILEEA 36
EMY23346.1        --------------MN---------F-LSLKKIFKLYFFLWVSFFGIDCKKNAVEILEEA 36
QOI42007.1        --------------MN---------F-LSLKKIFKLYFFLWVSFFGIDCKKNAVEILEEA 36
ASV07105.1        --------------MN---------F-LSLKKIFKLYFFLWVSFFGIDCKKNAVEILEEA 36
EJO80485.1        --------------MN---------F-LSLKKIFKLYFFLWVSFFGIDCKKNAVEILEEA 36
EKN96857.1        --------------MN---------F-LSLKKIFKLYFFLWVSFFGIDCKKNAVEILEEA 36
MCR8646250.1      --------------MN---------F-LSLKKIFKIYFFLWVSFFGIDCKKNAVEILEEA 36
OAM85608.1        --------------MN---------F-LSLKKIFKIYFFLWVSFFGIDCKKNAVEILEEA 36
QOI37956.1        --------------MN---------F-LSLKKIFKIYFFLWVSFFGIDCKKNAVEILEEA 36
OOV43912.1        --------------MN---------F-VFLKKIFRIYFFFWISIFGIDCKKNAVEIIEEA 36
```

```
EKQ84072.1        --------------MN---------F-LFLKKIFRIYFFFWISIFGIDCKKNAVEILEEA 36
EPG48343.1        --------------MN---------F-LFLKKIFRIYFFFWISIFGIDCKKNAVEILEEA 36
EJO71711.1        --------------MN---------F-LFLKKIFRIYFFFWISIFGIDCKKNAVEILEEA 36
EMO40832.1        --------------MN---------S-LSLKKIFRIYFFFLISIFWIDCKKNAVEILEEA 36
EQA71632.1        --------------MN---------S-LSLKKIFRIYFFFLISIFWIDCKKSAIEILEEA 36
EMY77189.1        MQVKSMILVGTLEKFAFLILFIPKTFQLVGKVMIAIGWLFFASIFWIDCKKTAEEILGEA 60
EMJ90581.1        --------------MN---------F-PSLKKFVTIGSLFLAPIFWIDCKKNAEEILREA 36
EQA82096.1        --------------MN---------F-PSLKKFVTIGSLFLAPIFWIDCKKNAEEILREA 36
EKT85788.2        -------MIEGVERMN---------F-PLLKRFLTIGFICFTASLEIDCKKNTEEILEEA 43
EMN21750.1        --------------MN---------F-PLLKRFLTIGFICFTASLEIDCKKNTEEILEEA 36
EQA64549.1        -------MREGVKRMN---------F-PLLKEFLAIGFICFTAS--FDCKKNAEEILGEA 41
AFV46185.1        ------------------------------------GFLCFTAS--FDCKKNAEEILGEA 22
PTM44326.1        -------MREGAKRMN---------F-PLLKEFLVIGFVCFTAS--FDCKKNAEEILGEA 41
AFV46188.1        ------------------------------------GFVCFTAS--FDCKKNAEEILGEA 22
ALO26640.1        -------MREGAKRMN---------F-PLLKEFLVIGFVCFTAS--FDCKKNAEEILGEA 41
AFV46187.1        ------------------------------------GFVCFTAS--FDCKKNAEEILGEA 22
AFV46186.1        ------------------------------------GFVCFTAS--FDCKKNAEEILGEA 22
AXX17321.1        --------------MN---------F-PLLKEFLVIGFVCFTAS--FDCKKNAEEILGEA 34
EMO62774.1        --------------MN---------F-PLLKEFLVIGFVCFTAS--FNCKKNAEEILGEA 34
ABJ76745.1        --------------MN---------F-PLLKEFLVIGFVCFTAS--FNCKKNAEEILGEA 34
                                                        .       ::***.: **: **
```

| | | |
|---|---|---|
| OCA00011.1 | KRKSESIQILDLGMQKLTSIPEGVCSFPNLTQLDLRLNSLNSLPGWIGACKNLEQINLFG | 96 |
| EMO02625.1 | KRKSESIQILDLGMQKLTSIPEGVCSFPNLTQLDLRLNSLNSLPGWIGACKNLEQINLFG | 96 |
| EMF71629.1 | KRKSESIQILDLGMQKLTSIPEGVCSFPNLTQLDLRLNSLNSLPGWIGACKNLEQINLFG | 96 |
| QOI35068.1 | KRKSESIQILDLGMQKLTSIPEGVCSFPNLTQLDLRLNSLNSLPGWIGACKNLEQINLFG | 96 |
| EMN63541.1 | KRKSESIQILDLGMQKLTSIPEGVCSFPNLTQLDLRLNSLNSLPGWIGACKNLEQINLFG | 96 |
| EMP06413.1 | KRKSESIQILDLGMQKLTSIPEGVCSFPNLTQLDLRLNSLNSLPGWIGACKNLEQINLFG | 96 |
| EMN29390.1 | KRKSESIQILDLGMQKLTSIPEGVCSFPNLTQLDLRLNSLNSLPGWIGACKNLEQINLFG | 96 |
| EMN70889.1 | KRKSESIQILDLGMQKLTSIPEGVCSFPNLTQLDLRLNSLNSLPGWIGACKNLEQINLFG | 96 |
| EMN78462.1 | KRKSESIQILDLGMQKLTSIPEGVCSFPNLTQLDLRLNSLNSLPGWIGACKNLEQINLFG | 96 |
| AKH76669.1 | KRKSESIQILDLGMQKLTSIPEGVCSFPNLTQLDLRLNSLNSLPGWIGACKNLEQINLFG | 96 |
| EMG10292.1 | KRKSESIQILDLGMQKLTSIPEGVCSFPNLTQLDLRLNSLNSLPGWIGACKNLEQINLFG | 96 |
| EMY23346.1 | KRKSESIQILDLGMQKLTSIPEGVCSFPNLTQLDLRLNSLNSLPGWIGACKNLEQINLFG | 96 |
| QOI42007.1 | KRKSESIQILDLGMQKLTSIPEGVCSFPNLTQLDLRLNSLNSLPGWIGACKNLEQINLFG | 96 |
| ASV07105.1 | KRKSESIQILDLGMQKLTSIPEGVCSFPNLTQLDLRLNSLNSLPGWIGACKNLEQINLFG | 96 |
| EJO80485.1 | KRKSESIQILDLGMQKLTSIPEGVCSFPNLTQLDLRLNSLNSLPGWIGACKNLEQINLFG | 96 |
| EKN96857.1 | KRKSESIQILDLGMQKLTSIPEGVCSFPNLTQLDLRLNSLNSLPGWIGACKNLEQINLFG | 96 |
| MCR8646250.1 | KRKSESIQILDLGMQKLTSIPEGVCSFPNLTQLDLRLNSLNSLPGWIGACKNLEQINLFG | 96 |
| OAM85608.1 | KRKSESIQILDLGMQKLTSIPEGVCSFPNLTQLDLRLNSLNSLPGWIGACKNLEQINLFG | 96 |
| QOI37956.1 | KRKSESIQILDLGMQKLTSIPEGVCSFPNLTQLDLRLNSLNSLPGWIGACKNLEQINLFG | 96 |
| OOV43912.1 | KRKSESVQILDLGMQKLTSIPEGICSFPNLTQLDLRLNSLNSLPDWIGTCKNLEQINLFG | 96 |
| EKQ84072.1 | KRKSESVQILDLGMQKLTSIPEGICSFPNLTQLDLRLNSLNSLTDWIGTCKNLEQINLFG | 96 |
| EPG48343.1 | KRKSESVQILDLGMQKLTSIPEGICSFPNLTQLDLRLNSLNSLPDWIGTCKNLEQINLFG | 96 |
| EJO71711.1 | KRKSESVQILDLGMQKLTSIPEGICSFPNLTQLDLRLNSLNSLPDWIGTCKNLEQINLFG | 96 |

```
EMO40832.1    KKKSESVQILDLGMQKLTSIPEGVCSFPNLTQLDLRLNSLSFLPDWIGTCKNLEQVNLFG 96
EQA71632.1    KRKSESVQILDLGMQKLTSIPEGVCSFPNLTQLDLRLNSLNFLPDWIGTCKNLEQVNLFG 96
EMY77189.1    KASPSSVLLLDLGMQKLSSIPEGICSFPNLTKLDLRLNSLTSLPESVGECKNLEQLNLFG 120
EMJ90581.1    NTKPESVRVLDLGMQKLSSVPDGVCGFPNLTNLDLRLNSLTSLPEFIGECKNLEQLNLFG 96
EQA82096.1    NTKPESVRVLDLGMQKLSSVPDGVCGFPNLTNLDLRLNSLTSLPEFIGECKNLEQLNLFG 96
EKT85788.2    KARPESVQTLDFGMRKLSAVPEGVCGFPNLIKLDLRLNSLTSLPEFIGECKSLEQLNLFG 103
EMN21750.1    KARPESVQTLDFGMRKLSAVPEGVCGFPNLIKLDLRLNSLTSLPEFIGECKSLEQLNLFG 96
EQA64549.1    KAKPELVQTLDFGMQKLSTVPEGVCGFPNLTKLDLRLNSLSSLPDEIGDCKNLEQLNLFG 101
AFV46185.1    KAKPELVQTLDFGMQKLSTVPEEVCGFPNLTKLDLRLNSLTFLPEFIGECKRLEQLNLFG 82
PTM44326.1    KAKPELVQTLDFGMQKLSTVPEEVCGFPNLTKLDLRLNSLTFLPEFIGECKRLEQLNLFG 101
AFV46188.1    KAKPELVQTLDFGMQKLSTVPEEVCGFPNLTKLDLRLNSLTFLPEFIGECKRLEQLNLFG 82
ALO26640.1    KAKPELVQTLDFGMQKLSTVPEEVCGFPNLTKLDLRLNSLTFLPEFIGECKRLEQLNLFG 101
AFV46187.1    KAKPELVQTLDFGMQKLSTVPEEVCGFPNLTKLDLRLNSLTFLPEFIGECKRLEQLNLFG 82
AFV46186.1    KAKPELVQTLDFGMQKLSTVPEEVCGFPNLTKLDLRLNSLTFLPEFIGECKRLEQLNLFG 82
AXX17321.1    KAKPELVQTLDFGMQKLSTVPEEVCGFPNLTKLDLRLNSLTFLPEFIGECKRLEQLNLFG 94
EMO62774.1    KAKPELVQTLDFGMQKLSTVPEGVCGFPNLTKLDLRLNSLTFLPEFIGECKRLEQLNLFG 94
ABJ76745.1    KAKPELVQTLDFGMQKLSTVPEGVCGFPNLTKLDLRLNSLTFLPEFIGECKRLEQLNLFG 94
              :   . :  **:**:**:::*: :*.**** :********. *   :* ** ***:****
```

| | | |
|---|---|---|
| OCA00011.1 | NDLNTVPSSFSKLKNLKVLLLGNNDFTFLPSELLFLPLLKTLYLDQNKLTLTETDVEILA | 156 |
| EMO02625.1 | NDLNTVPSSFSKLKNLKVLLLGNNDFTFLPSELLFLPLLKTLYLDQNKLTLTETDVEILA | 156 |
| EMF71629.1 | NDLNTVPSSFSKLKNLKVLLLGNNDFTFLPSELLFLPLLKTLYLDQNKLTLTETDVEILA | 156 |
| QOI35068.1 | NDLNTVPSSFSKLKNLKVLLLGNNDFTFLPSELLFLPLLKTLYLDQNKLTLTETDVEILA | 156 |
| EMN63541.1 | NDLNTVPSSFSKLKNLKVLLLGNNDFTFLPSELLFLPLLKTLYLDQNKLTLTETDVEILA | 156 |
| EMP06413.1 | NDLNTVPSSFSKLKNLKVLLLGNNDFTFLPSELLFLPLLKTLYLDQNKLTLTETDVEILA | 156 |
| EMN29390.1 | NDLNTVPSSFSKLKNLKVLLLGNNDFTFLPSELLFLPLLKTLYLDQNKLTLTETDVEILA | 156 |
| EMN70889.1 | NDLNTVPSSFSKLKNLKVLLLGNNDFTFLPSELLFLPLLKTLYLDQNKLTLTETDVEILA | 156 |
| EMN78462.1 | NDLNTVPSSFSKLKNLKVLLLGNNDFTFLPSELLFLPLLKTLYLDQNKLTLTETDVEILA | 156 |
| AKH76669.1 | NDLNTVPSSFSKLKNLKVLLLGNNDFTFLPSELLFLPLLKTLYLDQNKLTLTETDVEILA | 156 |
| EMG10292.1 | NDLNTVPSSFSKLKNLKVLLLGNNDFTFLPSELLFLPLLKTLYLDQNKLTLTETDVEILA | 156 |
| EMY23346.1 | NDLNTVPSSFSKLKNLKVLLLGNNDFTFLPSELLFLPLLKTLYLDQNKLTLTETDVEIIA | 156 |
| QOI42007.1 | NDLNTVPSSFSKLKNLKVLLLGNNDFTFLPSELLFLPLLKTLYLDQNKFTLTETDVEILA | 156 |
| ASV07105.1 | NDLNTVPSSFSKLKNLKVLLLGNNDFTFLPSELLFLPLIKTLYLDQNKLTLTETDVEILA | 156 |
| EJO80485.1 | NDLNTVPSSFSKLKNLKVLLLGNNDFTFLPSELLFLPLIKTLYLDQNKLTLTETDVEILA | 156 |
| EKN96857.1 | NDLNTVPSSFSKLKNLKVLLLGNNDFTFLPSELLFLPLIKTLYLDQNKLTLTETDVEILA | 156 |
| MCR8646250.1 | NDLNTVPSSFSKLKNLKVLLLGNNDFTFLPSELLFLPLIKTLYLDQNKLTLTETDVEILA | 156 |
| OAM85608.1 | NDLNTVPSSFSKLKNLKVLLLGNNDFTFLPSELLFLPLIKTLYLDQNKLTLTETDVEILA | 156 |
| QOI37956.1 | NDLNTVPSSFSKLKNLKVLLLGNNDFTFLPSELLFLPLIKTLYLDQNKLTLTETDVEILA | 156 |
| OOV43912.1 | NDLATIPSSFSKLKNLKVLLVGNNDFAFLPSELLFLPLIKILYLDQNKLTLTETDVEILS | 156 |
| EKQ84072.1 | NDLATIPSSFSKLKNLKVLLVGNNDFAFLPSELLFLPLIKILYLDQNKLTLTETDVEILA | 156 |
| EPG48343.1 | NDLATIPSSFSKLKNLKVLLVGNNDFAFLPSELLFLPLIKILYLDQNKLTLTETDVEILA | 156 |
| EJO71711.1 | NDLATIPSSFSKLKNLKVLLVGNNDFAFLPSELLFLPLIKILYLDQNKLTLTETDVEILA | 156 |

```
EMO40832.1        NDLTTVPSSFSKLKNLKVLLLGNNDFTFLPSELLFLPLIKTLYLDQNKLTLTETDIEILS 156
EQA71632.1        NDLTTVPSSFSKLKNLKVLLLGNNDFTFLPSELLFLPLIKTLYFDQNKLTLTETDIEILS 156
EMY77189.1        NDLTTFPASFSKLKNLKVLLAGNNDLTVLPSELLFLPRIKTLYLDQNKITLTETDVEILA 180
EMJ90581.1        NDLGTFPASISKLKNLKVLLAGNNDFAALPSELLFLPSIKTLYLDRNKLTLTETDVEILA 156
EQA82096.1        NDLGTFPASISKLKNLKVLLAGNNDFAALPSELLFLPSIKTLYLDRNKLTLTETDVEILA 156
EKT85788.2        NDLTTLPSTFSKLKNLKVLLAGSNDFTVLPSELLFLPSIKTLYVDRNRLTLTETDVEILA 163
EMN21750.1        NDLTTLPSTFSKLKNLKVLLAGSNDFTVLPSELLFLPSIKTLYVDRNRLTLTETDVEILA 156
EQA64549.1        NDLTTFPAALSKLKKLRILLAGNNDLKFLPSELLFLPEIKTIYMDQNKLTLTETDVEILA 161
AFV46185.1        NDLTTFPSTFSKLKNLKVLLAGNNDFTILPSELLFLPLIKILYVDRNKLTLTETDVEILA 142
PTM44326.1        NDLTTFPSTFSKLKNLKVLLAGNNDFTILPSELLFLPLIKILYVDRNKLTLTETDVEILA 161
AFV46188.1        NDLTTFPSTFSKLKNLKVLLAGNNDFTILPSELLFLPLIKILYVDRNKLTLTETDVEILA 142
ALO26640.1        NDLTTFPSTFSKLKNLKVLLAGNNDFTILPSELLFLPLIKILYVDRNKLTLTETDVEILA 161
AFV46187.1        NDLTTFPSTFSKLKNLKVLLAGNNDFTILPSELLFLPLIKILYVDRNKLTLTETDVEILA 142
AFV46186.1        NDLTTFPSTFSKLKNLKVLLAGNNDFTILPSELLFLPLIKILYVDRNKLTLTETDVEILA 142
AXX17321.1        NDLTTFPSTFSKLKNLKVLLAGNNDFTILPSELLFLPLIKILYVDRNKLTLTETDVEILA 154
EMO62774.1        NDLTTFPSTFSKLKNLKVLLAGNNDFTILPSELLFLPLIKILYVDRNKLTLTETDVEILA 154
ABJ76745.1        NDLTTFPSTFSKLKNLKVLLAGNNDFTILPSELLFLPLIKILYVDRNKLILTETDVEILA 154
                  *** *.*:::****:*::** *.**:  ********* :* :*.*:*:: *****:**::
```

| | | |
|---|---|---|
| OCA00011.1 | SLSSLEELDLNLNSGIKVLPSNYNKLKNLTHLKRLNIKKTSLKGEDADKLQAVLPNTRID | 216 |
| EMO02625.1 | SLSSLEELDLNLNSGIKVLPSNYNKLKNLTHLKRLNIKKTSLKGEDADKLQAVLPNTRID | 216 |
| EMF71629.1 | SLSSLEELDLNLNSGIKVLPSNYNKLKNLTHLKRLNIKKTSLKGEDADKLQAVLPNTRID | 216 |
| QOI35068.1 | SLSSLEELDLNLNSGIKVLPSNYNKLKNLTHLKRLNIKKTSLKGEDADKLQAVLPNTRID | 216 |
| EMN63541.1 | SLSSLEELDLNLNSGIKVLPSNYNKLKNLTHLKRLNIKKTSLKGEDADKLQAVLPNTRID | 216 |
| EMP06413.1 | SLSSLEELDLNLNSGIKVLPSNYNKLKNLTHLKRLNIKKTSLKGEDADKLQAVLPNTRID | 216 |
| EMN29390.1 | SLSSLEELDLNLNSGIKVLPSNYNKLKNLTHLKRLNIKKTSLKGEDADKLQAVLPNTRID | 216 |
| EMN70889.1 | SLSSLEELDLNLNSGIKVLPSNYNKLKNLTHLKRLNIKKTSLKGEDADKLQAVLPNTRID | 216 |
| EMN78462.1 | SLSSLEELDLNLNSGIKVLPSNYNKLKNLTHLKRLNIKKTSLKGEDADKLQAVLPNTKID | 216 |
| AKH76669.1 | SLSSLEELDLNLNSGIKVLPSNYNKLKNLTHLKRLNIKKTSLKGEDADKLQAVLPNTRID | 216 |
| EMG10292.1 | SLSSLEELDLNLNSGIKVLPSNYNKLKNLTHLKRLNIKKTSLKGEDADKLQAVLPNTRID | 216 |
| EMY23346.1 | SLSSLEELDLNLNSGIKVLPSNYNKLKNLTHLKRLNIKKTSLKGEDADKLQAVLPNTRID | 216 |
| QOI42007.1 | SLSSLEELDLNLNSGIKVLPSNYNKLKNLTHLKRLNIKKTSLKGEDADKLQAVLPNTRID | 216 |
| ASV07105.1 | SLSSLEELDLNLNSGIKVLPSNYNKLKNLTHLKRLNIKKTSLKGEDADKLQAVLPNTRID | 216 |
| EJO80485.1 | SLSSLEELDLNLNSGIKVLPSNYNKLKNLTHLKRLNIKKTSLKGEDADKLQAVLPNTRID | 216 |
| EKN96857.1 | SLSSLEELDLNLNSGIKVLPSNYNKLKNLTHLKRLNIKKTSLKGEDADKLQAVLPNTRID | 216 |
| MCR8646250.1 | SLSSLEELDLNLNSGIKVLPSNYNKLKNLTHLKRLNIKKTSLKGEDADKLQAVLPNTRID | 216 |
| OAM85608.1 | SLSSLEELDLNLNSGIKVLPSNYNKLKNLTHLKRLNIKKTSLKGEDADKLQAVLPNTRID | 216 |
| QOI37956.1 | SLSSLEELDLNLNSGIKVLPSNYNKLKNLTHLKRLNIKKTSLKGEDADKLQAVLPNTRID | 216 |
| OOV43912.1 | SLSSLEELDLNLNSGIKTLPSNYNKLKNLNRLKRLNIKKTSLKGEDADKLQAILPNTKID | 216 |
| EKQ84072.1 | SLSSLEELDLNLNSGIKTLPSNYNKLKNLNRLKRLNIKKTSLKGEDADKLQAILPNTKID | 216 |
| EPG48343.1 | SLSSLEELDLNLNSGIKTLPSNYNKLKNLNRLKRLNIKKTSLKGEDADKLQAILPNTKID | 216 |
| EJO71711.1 | SLSSLEELDLNLNSGIKTLPSNYNKLKNLNRLKRLNIKKTSLRGEDADKLQAILPNTKID | 216 |

```
EMO40832.1        SLSGLEELDLNLNPGIKILPSNYNKLKNLTHLKRLNIKKTLLKGEDAGKLQAILPNTKID 216
EQA71632.1        SLSGLEELDLNLNPGIKILPSNYNKLKNLTHLKRLNIKKTLLKGEDADKLQAILPNTKID 216
EMY77189.1        SLSELEELDLNLNTGIKTLPSNYGKLKSLARLKKLNIKKTSLKGEDAEKLQAILPNTKID 240
EMJ90581.1        SLSGLEELDLNLNSDIKALPSNYKKLKNLTRLKRLNIKKTSLKGEDAEKLQAILPNTKID 216
EQA82096.1        SLSGLEELDLNLNSDIKALPSNYKKLKNLTRLKRLNIKKTSLKGEDAEKLQAILPNTKID 216
EKT85788.2        SLSSLEELDLNQNSGIGTLPFNYEKLRNLTGLKRLNIKKTSLKGEDAGKLQAILPNAKID 223
EMN21750.1        SLSSLEELDLNQNSGIGTLPFNYEKLRNLTGLKRLNIKKTSLKGEDAGKLQAILPNAKID 216
EQA64549.1        SLSSLEELDLNLNLGIKTLPFNYEKLGNLTNLKRLNIKKTSLKGEDADKLQAILPNTKID 221
AFV46185.1        SLSSLEELDLSLNSGIKALPFNYEKLVNLINLKRLNIKKTSLKGEDADKLQAILRNTKID 202
PTM44326.1        SLSSLEELDLSLNSGIKALPFNYEKLVNLINLKRLNIKKTSLKGEDADKLQAILPNTKID 221
AFV46188.1        SLSSLEELDLSLNSGIKALPFNYEKLVNLINLKRLNIKKTSLKGEDADKLQAILPNTKID 202
ALO26640.1        SLSSLEELDLSLNSGIKALPFNYEKLVNLINLKRLNIKKTSLKGEDADKLQAILPNTKID 221
AFV46187.1        SLSSLEELDLSLNSGIKALPFNYEKLVNLINLKRLNIKKTSLKGEDADKLQAILPNTKID 202
AFV46186.1        SLSSLEELDLSLNSGIKALPFNYEKLVNLINLKRLNIKKTSLKGEDADKLQAILPNTKID 202
AXX17321.1        SLSSLEELDLSLNSGIKALPFNYEKLVNLINLKRLNIKKTSLKGEDADKLQAILPNTKID 214
EMO62774.1        SLSSLEELDLSLNSGIKALPFNYEKLVNLINLKRLNIKKTSLKGEDADKLQAILPNTKID 214
ABJ76745.1        SLSSLEELDLSLNSGIKALPFNYEKLVNLINLKRLNIKKTSLKGEDADKLQAILPNTKID 214
                  *** ******. * .*  ** ** ** .*  **:****** *:**** ****:* *::**
```

| | |
|---|---|
| OCA00011.1 | Y 217 |
| EMO02625.1 | Y 217 |
| EMF71629.1 | Y 217 |
| QOI35068.1 | Y 217 |
| EMN63541.1 | Y 217 |
| EMP06413.1 | Y 217 |
| EMN29390.1 | Y 217 |
| EMN70889.1 | Y 217 |
| EMN78462.1 | Y 217 |
| AKH76669.1 | Y 217 |
| EMG10292.1 | Y 217 |
| EMY23346.1 | Y 217 |
| QOI42007.1 | Y 217 |
| ASV07105.1 | Y 217 |
| EJO80485.1 | Y 217 |
| EKN96857.1 | Y 217 |
| MCR8646250.1 | Y 217 |
| OAM85608.1 | Y 217 |
| QOI37956.1 | Y 217 |
| OOV43912.1 | Y 217 |
| EKQ84072.1 | Y 217 |
| EPG48343.1 | Y 217 |
| EJO71711.1 | Y 217 |

| | |
|---|---|
| EMO40832.1 | Y 217 |
| EQA71632.1 | Y 217 |
| EMY77189.1 | Y 241 |
| EMJ90581.1 | Y 217 |
| EQA82096.1 | Y 217 |
| EKT85788.2 | Y 224 |
| EMN21750.1 | Y 217 |
| EQA64549.1 | Y 222 |
| AFV46185.1 | Y 203 |
| PTM44326.1 | Y 222 |
| AFV46188.1 | Y 203 |
| ALO26640.1 | Y 222 |
| AFV46187.1 | Y 203 |
| AFV46186.1 | Y 203 |
| AXX17321.1 | Y 215 |
| EMO62774.1 | Y 215 |
| ABJ76745.1 | Y 215 |
| | * |

| Accession | Accession from 100 Hit | Description | Max Score | Total Score | Query Cover | E value | Per. Ident |
|---|---|---|---|---|---|---|---|
| ALO26640.1 | ALO26640.1 | leucine rich repeat protein [Leptospira borgpetersenii serovar Ballum] | 359 | 359 | 100% | 8E-124 | 100.00% |
| PTM44326.1 | ALO26640.1 | leucine rich repeat (LRR) protein [Leptospira borgpetersenii serovar Javanica] | 359 | 359 | 100% | 8E-124 | 100.00% |
| AFV46187.1 | AFV46186.1 | leucine-rich repeat protein, partial [Leptospira borgpetersenii serovar Mini] | 358 | 358 | 100% | 6.00E-124 | 100.00% |
| AFV46186.1 | AFV46186.1 | leucine-rich repeat protein, partial [Leptospira borgpetersenii serovar Ballum] | 358 | 358 | 100% | 6E-124 | 100.00% |
| AFV46188.1 | AFV46186.1 | leucine-rich repeat protein, partial [Leptospira borgpetersenii serovar Sejroe] | 358 | 358 | 100% | 6E-124 | 100.00% |
| AXX17321.1 | WP_002731979.1 | hypothetical protein C4Q31_14790 [Leptospira borgpetersenii serovar Ceylonica] | 358 | 358 | 100% | 1E-123 | 100.00% |
| EMO62774.1 | EMO62774.1 | leucine rich repeat protein [Leptospira borgpetersenii serovar Pomona str. 200901868] | 354 | 354 | 100% | 7.00E-122 | 99.01% |
| AFV46185.1 | AFV46185.1 | leucine-rich repeat protein, partial [Leptospira borgpetersenii serovar Javanica] | 354 | 354 | 100% | 3E-122 | 99.01% |
| ABJ76745.1 | WP_011669694.1 | Leucine-rich-repeat protein [Leptospira borgpetersenii serovar Hardjo-bovis str. JB197] | 352 | 352 | 100% | 4E-121 | 98.52% |
| EQA64549.1 | EQA64549.1 | leucine rich repeat protein [Leptospira alexanderi serovar Manhao 3 str. L 60] | 312 | 312 | 100% | 2E-105 | 86.70% |
| EKT85788.2 | EKT85788.2 | hypothetical protein LSS_15741 [Leptospira santarosai serovar Shermani str. LT 821] | 305 | 305 | 100% | 1E-102 | 85.85% |
| EMN21750.1 | WP_004471631.1 | leucine rich repeat protein [Leptospira santarosai serovar Arenal str. MAVJ 401] | 305 | 305 | 100% | 2E-102 | 85.85% |
| EMJ90581.1 | WP_020775386.1 | leucine rich repeat protein [Leptospira alstonii serovar Sichuan str. 79601] | 278 | 278 | 96% | 6E-92 | 83.59% |
| EQA82096.1 | WP_020775386.1 | leucine rich repeat protein [Leptospira alstonii serovar Pingchang str. 80-412] | 278 | 278 | 96% | 6E-92 | 83.59% |
| EMY77189.1 | EMY77189.1 | leucine rich repeat protein [Leptospira weilii serovar Ranarum str. ICFT] | 277 | 277 | 100% | 4E-91 | 78.05% |
| EPG48343.1 | WP_004759233.1 | leucine rich repeat protein [Leptospira kirschneri serovar Cynopteri str. 3522 CT] | 270 | 270 | 95% | 2E-88 | 81.44% |
| EJO71711.1 | WP_004750770.1 | leucine rich repeat protein [Leptospira kirschneri serovar Grippotyphosa str. RM52] | 269 | 269 | 95% | 3E-88 | 80.93% |
| OOV43912.1 | WP_082292870.1 | hypothetical protein B1J93_06785 [Leptospira kirschneri serovar Pomona] | 268 | 268 | 95% | 1.00E-87 | 80.41% |
| EKQ84072.1 | WP_004760926.1 | leucine rich repeat protein [Leptospira kirschneri serovar Grippotyphosa str. Moskva] | 266 | 266 | 95% | 2E-87 | 80.93% |
| EMO40832.1 | WP_004438578.1 | leucine rich repeat protein [Leptospira noguchii serovar Autumnalis str. ZUN142] | 251 | 251 | 99% | 5E-81 | 77.23% |
| ASV07105.1 | WP_001010181.1 | hypothetical protein B2G47_15925 [Leptospira interrogans serovar Canicola] | 250 | 250 | 95% | 7.00E-81 | 80.41% |
| EJO80485.1 | WP_001010181.1 | leucine rich repeat protein [Leptospira interrogans serovar Pomona str. Kennewicki LC82-25] | 250 | 250 | 95% | 7.00E-81 | 80.41% |
| EKN96857.1 | WP_001010181.1 | leucine rich repeat protein [Leptospira interrogans serovar Pomona str. Pomona] | 250 | 250 | 95% | 7.00E-81 | 80.41% |
| MCR8646250.1 | WP_001010177.1 | hypothetical protein [Leptospira interrogans serovar Bataviae] | 250 | 250 | 95% | 8.00E-81 | 80.41% |
| OAM85608.1 | WP_001010177.1 | hypothetical protein A1343_17415 [Leptospira interrogans serovar Bataviae] | 250 | 250 | 95% | 8.00E-81 | 80.41% |
| QOI37956.1 | WP_001010177.1 | hypothetical protein Lepto1548_06450 [Leptospira interrogans serovar Bataviae] | 250 | 250 | 95% | 8.00E-81 | 80.41% |
| EQA71632.1 | WP_002177142.1 | leucine rich repeat protein [Leptospira noguchii serovar Panama str. CZ214] | 250 | 250 | 95% | 1E-80 | 79.90% |
| QOI35068.1 | WP_001010185.1 | hypothetical protein LeptoLang_13150 [Leptospira interrogans serovar Icterohaemorrhagiae] | 231 | 231 | 95% | 3.00E-73 | 79.90% |
| EMF71629.1 | WP_001010184.1 | leucine rich repeat protein [Leptospira interrogans serovar Canicola str. LT1962] | 231 | 231 | 95% | 3.00E-73 | 79.90% |
| EMO02625.1 | WP_001010184.1 | leucine rich repeat protein [Leptospira interrogans serovar Icterohaemorrhagiae str. Verdun [illegible] | 231 | 231 | 95% | 3.00E-73 | 79.90% |
| OCA00011.1 | WP_001010184.1 | Leucine rich repeat protein [Leptospira interrogans serovar Copenhageni/Icterohaemorrhagi[illegible] | 231 | 231 | 95% | 3.00E-73 | 79.90% |
| EMY23346.1 | WP_001010182.1 | leucine rich repeat protein [Leptospira interrogans serovar Australis str. 200703203] | 231 | 231 | 95% | 3.00E-73 | 79.38% |
| EMN70889.1 | WP_001010178.1 | leucine rich repeat protein [Leptospira interrogans serovar Bataviae str. UI 08561] | 231 | 231 | 95% | 3.00E-73 | 79.90% |
| EMN78462.1 | WP_002127204.1 | leucine rich repeat protein [Leptospira interrogans serovar Grippotyphosa str. UI 12764] | 231 | 231 | 95% | 2E-73 | 80.41% |
| EMN29390.1 | WP_001010185.1 | leucine rich repeat protein [Leptospira interrogans serovar Pyrogenes str. L0374] | 231 | 231 | 95% | 3E-73 | 79.90% |
| EMP06413.1 | WP_001010185.1 | leucine rich repeat protein [Leptospira interrogans serovar Pyrogenes str. 200701872] | 231 | 231 | 95% | 3E-73 | 79.90% |
| EMN63541.1 | WP_001010185.1 | leucine rich repeat protein [Leptospira interrogans serovar Pyrogenes str. R168] | 231 | 231 | 95% | 3E-73 | 79.90% |
| EMG10292.1 | WP_001010178.1 | leucine rich repeat protein [Leptospira interrogans serovar Grippotyphosa str. LT2186] | 231 | 231 | 95% | 3E-73 | 79.90% |
| AKH76669.1 | WP_001010178.1 | leucine rich repeat protein [Leptospira interrogans serovar Bratislava] | 231 | 231 | 95% | 3E-73 | 79.90% |
| QOI42007.1 | WP_192505591.1 | hypothetical protein Lepto782_06840 [Leptospira interrogans serovar Canicola] | 229 | 229 | 95% | 1.00E-72 | 79.38% |
